# Toward recovery-oriented perinatal healthcare: A participatory qualitative exploration of persons with lived experience and health providers’ views and experiences

**DOI:** 10.1192/j.eurpsy.2023.2464

**Published:** 2023-10-20

**Authors:** Marine Dubreucq, Mathilde Thiollier, Sarah Tebeka, Pierre Fourneret, Marion Leboyer, Sylvie Viaux-Savelon, Catherine Massoubre, Corinne Dupont, Julien Dubreucq

**Affiliations:** 1 Centre Referent de Rehabilitation Psychosociale, GCSMS REHACOOR 42, Saint-Étienne, France; 2 INSERM U1290, Research on Healthcare Performance (RESHAPE), University Lyon 1, Lyon, France; 3 Maman Blues Patient Representatives Association, Saint-Etienne, France; 4 Université de Paris, INSERM UMR1266, Institute of Psychiatry and Neurosciences, Team 1, Paris, France; 5 Department of Psychiatry, AP-HP, Louis Mourier Hospital, Colombes, France; 6 Department of Psychopathology of Child and Adolescent Development, Hospices Civils de Lyon, Lyon, France; 7 Marc Jeannerod Institute of Cognitive Sciences UMR 5229, CNRS & Claude Bernard University, Lyon, France; 8 Fondation Fondamental, Créteil, France; 9 Université Paris Est Créteil, INSERM U955, IMRB, Translational NeuroPsychiatry, Créteil, France; 10 AP-HP, Hôpitaux Universitaires Henri Mondor, Département Médico-Universitaire de Psychiatrie et d’Addictologie (DMU IMPACT), Fédération Hospitalo-Universitaire de Médecine de Précision en Psychiatrie (FHU ADAPT), Créteil, France; 11 Hôpital de la Croix-Rousse, Hospices Civils de Lyon, Lyon, France; 12 University Hospital of Saint-Étienne & EA 7423, Troubles du Comportement Alimentaire, Addictions et Poids Extrêmes (TAPE), Université Jean Monnet, Saint-Etienne, France; 13 University Claude Bernard Lyon 1, Research on Healthcare Performance (RESHAPE) INSERM U1290 & AURORE Perinatal Network, Hospices Civils de Lyon, Croix-Rousse Hospital, Lyon, France; 14 Department of Child and Adolescent Psychiatry, University Hospital of Saint-Étienne, Saint-Etienne, France

**Keywords:** coproduction with persons with lived experience, healthcare improvement, perinatal mental health disorders, personal recovery, stigma

## Abstract

**Background:**

Perinatal mental health disorders (PMHD) remain often undetected, undiagnosed, and untreated with variable access to perinatal mental health care (PMHC). To guide the design of optimal PMHC (i.e., coproduced with persons with lived experience [PLEs]), this qualitative participatory study explored the experiences, views, and expectations of PLEs, obstetric providers (OP), childcare health providers (CHPs), and mental health providers (MHPs) on PMHC and the care of perinatal depression.

**Methods:**

We conducted nine focus groups and 24 individual interviews between December 2020 and May 2022 for a total number of 84 participants (24 PLEs; 30 OPs; 11 CHPs; and 19 MHPs). The PLEs group included women with serious mental illness (SMI) or autistic women who had contact with perinatal health services. We recruited PLEs through social media and a center for psychiatric rehabilitation, and health providers (HPs) through perinatal health networks. We used the inductive six-step process by Braun and Clarke for the thematic analysis.

**Results:**

We found some degree of difference in the identified priorities between PLEs (e.g., personal recovery, person-centered care) and HPs (e.g., common culture, communication between providers, and risk management). Personal recovery in PMHD corresponded to the CHIME framework, that is, connectedness, hope, identity, meaning, and empowerment. Recovery-supporting relations and peer support contributed to personal recovery. Other factors included changes in the socio-cultural conception of the peripartum, challenging stigma (e.g., integrating PMH into standard perinatal healthcare), and service integration.

**Discussion:**

This analysis generated novel insights into how to improve PMHC for all users including those with SMI or autism.

## Introduction

Perinatal mental health disorders (PMHD; i.e., anxiety, nonpsychotic depressive episode, manic or psychotic episodes, post-traumatic stress disorder, and adjustment disorder occurring during pregnancy and the first year after childbirth) affect up to one in five women in high-income countries and are often associated with poor parental and child outcomes [1]. PMHD are in particular frequent in women with serious mental illness (SMI) (i.e., schizophrenia, bipolar disorder, and major depression) and autistic women [1, 2]. Despite an estimate cost of £8.1 billion per year in the United Kingdom (UK), PMHD remain predominantly unrecognized, undiagnosed, and untreated [1].

Guidelines and action plans from the WHO and many countries support prevention, early detection of PMHD, and improved access to specialist perinatal mental health services (SPMHS) [1]. Despite efforts to address gaps in perinatal mental health care (PMHC) in some countries (e.g., £365 million invested between 2016 and 2021 in England) the access to these services remain often heterogeneous (e.g., ¼ of the women in the UK had no access to SPMHS in 2018 [3]). In France, suicide is the leading cause of maternal mortality during the first year of life, responsible of approximately one death per month between 2013 and 2015 [4]. Of these deaths, 91% could be considered as avoidable because of nonoptimal care (i.e., lack of detection, referral, or treatment). Aligning with this, only 27.4% of the pregnant women reporting psychological distress in the French ELFE study had access to SPMHS [5] and large areas of France remain without access to outpatient SPMHS or mother-baby units.

In a recent systematic review, Webb et al. [3] identified barriers and facilitators to the implementation of SPMHC, influential at different levels across the care pathway. While a number of qualitative studies described the barriers to accessing SPMHS or to the implementation of SPMHC, there remain some limitations to the current body of evidence. First, most studies were conducted in the United States or the UK and did not compared the perspective of different stakeholders (e.g., solely mothers with PMHD or health providers [HPs]; mixed samples between mothers and obstetric providers [OPs]; [3, 6]). Second, while optimal service provision refers to person-centered care coproduced with persons with lived experience (PLEs; [3]), most studies did not involve researchers with lived experience nor used a participatory design. Third, research on personal recovery in PMHD remains scarce [7, 8]. Fourth, while men often experience depression during the peripartum (8–10% of the fathers; [9]), their place in the PMHC is mainly envisioned from the perspective of their partner, for example (lack of) partner support [3, 6]. Fifth, most studies excluded women with SMI or autistic mothers and did not cover preconception care [1, 2].

To improve PMHC in France, the first 1000 days national commission issued recommendations that will be completed by guidelines from the National Health Authority. To guide the design of an innovative optimal service provision, this qualitative participatory study explored the experiences, views, ideas, and expectations of (i) PLEs of PMHD, SMI, or autism, (ii) OPs, (iii) childcare health providers (CHPs), and (iv) mental health providers (MHPs) on how to improve PMHC and in particular the care of perinatal depression. France is a relevant setting to examine this question because of its long-established interest in perinatal mental health (PMH) and perinatal psychiatry [10]. We conducted nine focus groups and 24 individual interviews for a total number of 84 participants (24 PLEs, 30 OPs, 11 CHPs, and 19 MHPs).

## Methods

### Evidence before this study

We searched PubMed, Google Scholar, and Embase for articles published in English and in French between January 1, 2000, and May 22, 2023, using the search terms “improvement,” “perinatal (peripartum, antenatal, postnatal),” “mental health care,” “participatory (collaborative, coproduction),” and “recovery” (recovery-oriented, person-centered, treatment preferences). We screened the reference list of five systematic reviews on topics related to the improvement of PMHC. The search yielded 75 articles applicable to our study objective (Supplementary Table 1).

### Study design and participants

The present study used a qualitative participatory research design, combining focus groups (for providers) and in-depth individual interviews (for PLEs) conducted between December 2020 and May 2022. The consolidated criteria for reporting qualitative research (COREQ; [11]) were used to design the study protocol and report results. HPs were recruited through three perinatal health networks in Auvergne Rhône-Alpes (ELENA for Saint-Étienne, AURORE for Lyon and Auvergne Perinatal Health Network (RSPA) for Clermont-Ferrand) and a group of experts in PMH in Ile de France. An advert was distributed through the social media of Maman Blues, an association of persons with maternal distress, to recruit PLE of PMHD. Women with SMI and autistic women were recruited through a center for psychiatric rehabilitation from the REHABase network (Grenoble). Eligible participants in the PLEs group were adults (age > 18) with lived experience of PMHD (self-identified) and adults with a confirmed diagnosis of SMI (schizophrenia, bipolar disorder, or major depression; DSM-5; [12]) or autism spectrum disorder (DSM-5, [12]), who had a lived experience of PMHD or had contact with perinatal health services (i.e., through prior pregnancies or preconception care). Eligible participants in the HPs’ group were OPs (midwives and obstetricians), CHPs (pediatricians, general practitioners, pediatric nurses, childcare assistants), and MHPs (child and adolescents (C&A) psychiatrists, adult psychiatrists, psychologists, mental health nurses, social workers). The relevant Ethical Review Board (CPP-Ile de France I) approved the appraisal protocol on March 10, 2020, and all participants gave informed consent.

### Procedure

Researchers’ own position, views, and opinions can influence the research process [13]. We thus used a participatory research design (i.e., coproduction by academic researchers and a coresearcher with lived experience as equal partners; [14]) and adopted a reflexive position from the inception of the project to ensure that researchers’ feminist convictions did not dominate the study design or data collection and analysis. Nine focus groups and 24 individual interviews were conducted for a total number of 84 participants. To capture the complexity of the topic and to facilitate participants’ expression on sensitive information (i.e., their personal experiences, views, feelings, and attitudes; [15]), we conducted in-depth individual interviews for PLEs and separate focus groups for HPs according to their type of practice (i.e., OPs; CHPs; MHPs). Given the pandemic context, most individual interviews and focus groups were conducted online using secured video-conferencing solutions.

Focus groups are group discussions where the moderator uses a semi-structured group interview to address specific issues and to ensure that the discussion remains on the subject of interest. Apart from this artificial structure, efforts were made to create a group environment as close as possible to a naturally occurring social interaction. Participants were asked the same set of questions in the individual interviews and in focus groups (semi-structured interview in Supplementary Table 2). Participants were asked to discuss their personal experiences, views, feelings, and attitudes toward PMHC and in particular toward the care of perinatal depression (PPD) (e.g., the challenges they faced or anticipated and the resources they identified). They were encouraged to formulate their ideas on how to improve PMHC. We purposely put a focus on PPD for this study because this condition gained awareness in the general public and in HPs over the recent years (e.g., specific citation in the national “first 1000 days of life” commission report). General information was recorded for PLEs (age, gender, education, marital status, number of children, psychiatric diagnosis) and HPs (age, gender, profession, type of practice, for example, hospital or private practice, duration of professional experience and confidence when caring for patients with PPD). After participants consented to participate and agreed to the recording of the session, discussions lasted around 2 hours. Individual interviews and focus groups were conducted by at least two members of the research team, video and tape recorded and fully transcribed. In order to ensure the participation of all participants, the second moderator regularly invited those who did not spontaneously contribute to share their experience, thoughts and feelings about the topics covered. The first author checked the final transcription against the recordings.

### Data analysis

For the thematic analysis, we used an inductive, rather than theoretical, approach to qualitatively analyze the data (i.e., “bottom-up” identification of themes). More specifically, we followed the six-step process by Braun and Clarke [16]: researchers familiarized themselves with the data as a whole, generated initial codes, searched for themes, reviewed themes, named each defined theme, and produced the final report. Themes were refined by reexamining the coherence of data codes within each theme and the validity of each theme in relation with the whole dataset. Coder debriefings occurred throughout the analysis to review the identified themes and reach an agreement on coding discrepancies. To allow a deeper and broader understanding of the topic and reduce the risk of interpretation biases, we used methodological triangulation (i.e., individual interviews and focus groups), investigator triangulation (i.e., independent coding by two researchers with different backgrounds, a specialist midwife and a perinatal psychiatrist and review of all codes by a second perinatal psychiatrist and a coresearcher with lived experience of PMHD) and data triangulation (i.e., comparison of the perspective of various stakeholders – PLE and diverse HPs – on a same topic). Participants did not give their feedback on the results. We obtained code saturation and meaning saturation at the end of the study, that is, the point in the research process where no new information is discovered in data analysis and when no further dimensions, nuances, insights of issues can be found [17]. Given the inclusion of nonparents in the PLEs group could have influenced the interpretation of the results, we conducted an additional analysis after removing these participants to search for potential differences in the identified themes.

## Results

Nine focus groups and 24 individual interviews were conducted (*n* = 84 participants). The PLE group was composed of 4 women and 1 man with lived experience of PMHD, 9 women with SMI, and 10 autistic women. Of the PLE group, 62.5% had contact with perinatal health services through prior pregnancies (54.2% had children of any age). In the SMI and autism subgroups, three mothers also reported a lived experience of PMHD (37.5%). The provider group was composed of 30 OPs (27 midwives, 3 obstetricians), 11 CHPs (4 pediatricians, 3 general practitioners, 3 pediatric nurses, 1 childcare assistant), and 19 MHPs (3 C&A psychiatrists, 4 adult psychiatrists, 8 psychologists, 3 MH nurses and 1 social worker). Sample characteristics are presented in Table 1.

**Table 1.** Sample characteristics

	Obstetric providers (*n* = 30)	Childcare health providers (*n* = 11)	Mental health providers (*n* = 19)	Persons with lived experience (*n* = 24)
Mean age (years)				
Mean (SD)	45.1 (9.96)	47.7 (12.67)	41.68 (7.94)	33.23 (4.41)
Range	32–64	28–74	30–60	22–40
Gender				
Female	30 (100)	10 (90.9%)	17 (89.5%)	23 (95.8%)
Male	0 (0%)	1 (9.1%)	2 (10.5%)	1 (4.2%)
Profession				
Midwives	27 (90%)			
Obstetricians	3 (10%)			
Pediatricians		4 (36.3%)		
General practitioners		3 (27.3%)		
Pediatric nurses		3(27.3%)		
Childcare assistants		1 (9.1%)		
C&A psychiatrists			3 (15.8%)	
Adult psychiatrist			4 (21%)	
Psychologists			8 (42.1%)	
Mental health nurses			3 (15.8%)	
Social workers			1 (5.3%)	
Type of practice				
Hospital	17 (56.6%)	3 (27.3%)	14 (73.7%)	
Private practice	6 (20%)	5 (45.4%)	5 (26.3%)	
Mixed	2 (6.7%)	0 (0%)	0 (0%)	
Territorial	5 (16.7%)	3 (27.3%)	0 (0%)	
Median duration of professional experience				
Median (SD)	11.5 (9.06)	18.85 (10.47)	5.83 (3.62)	
Range	0.08–37	2.5–40	1–15	
Contact with women with peripartum depression (within the 3 last months)				
Yes	9 (30%)	5 (45.4%)	19 (100%)	
No	21 (70%)	6 (54.6%)	0 (0%)	
Confidence when caring for women with peripartum depression^a^				
Mean (SD)	2.83 (1.02)	1.17 (1.36)	3.88 (0.96)	
Range	1–5	1–5	1–5	
Education level (years)				
Mean (SD)	17.50 (0.28)	19.27 (1.00)	18.74 (0.79)	15.65 (2.48)
Range	17–22	13–22	15–25	11–22
Diagnosis^b^				
Peripartum depression				8 (33.3%)
Schizophrenia spectrum disorder				3 (12.5%)
Bipolar disorders				5 (20.8%)
Borderline personality disorder				2 (8.3%)
Autism spectrum disorder				10 (41.7%)
Lived experience of PMHD (for parents with SMI and autistic parents only; *n* = 8)				
Yes				3 (37.5%)
No				5 (62.5%)
Marital status				
In a couple				18 (75%)
Single				6 (25%)
Contact with perinatal health services through priori pregnancies				
Yes				15 (62.5%)
No				9 (37.5%)
Parenthood status				
Nonparent				11 (45.8%)
Parent				13 (54.2%)

a From 1 (Not comfortable at all) to 5 (very comfortable).

b Four participants had two cooccurring conditions (one woman with bipolar disorder and borderline personality disorder; three autistic mothers with peripartum depression).

**Figure 1.**
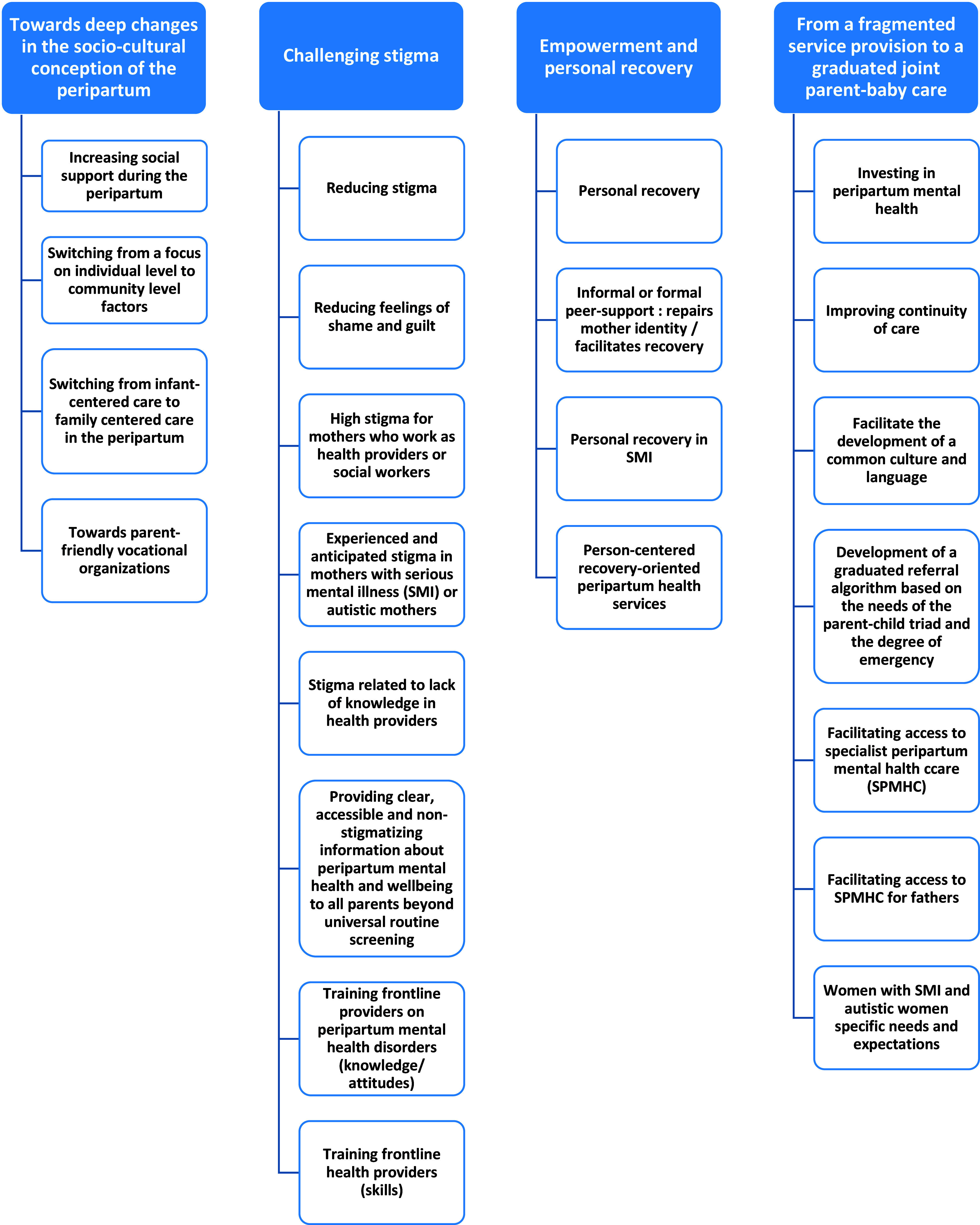
Thematic tree.

**Figure 2.**
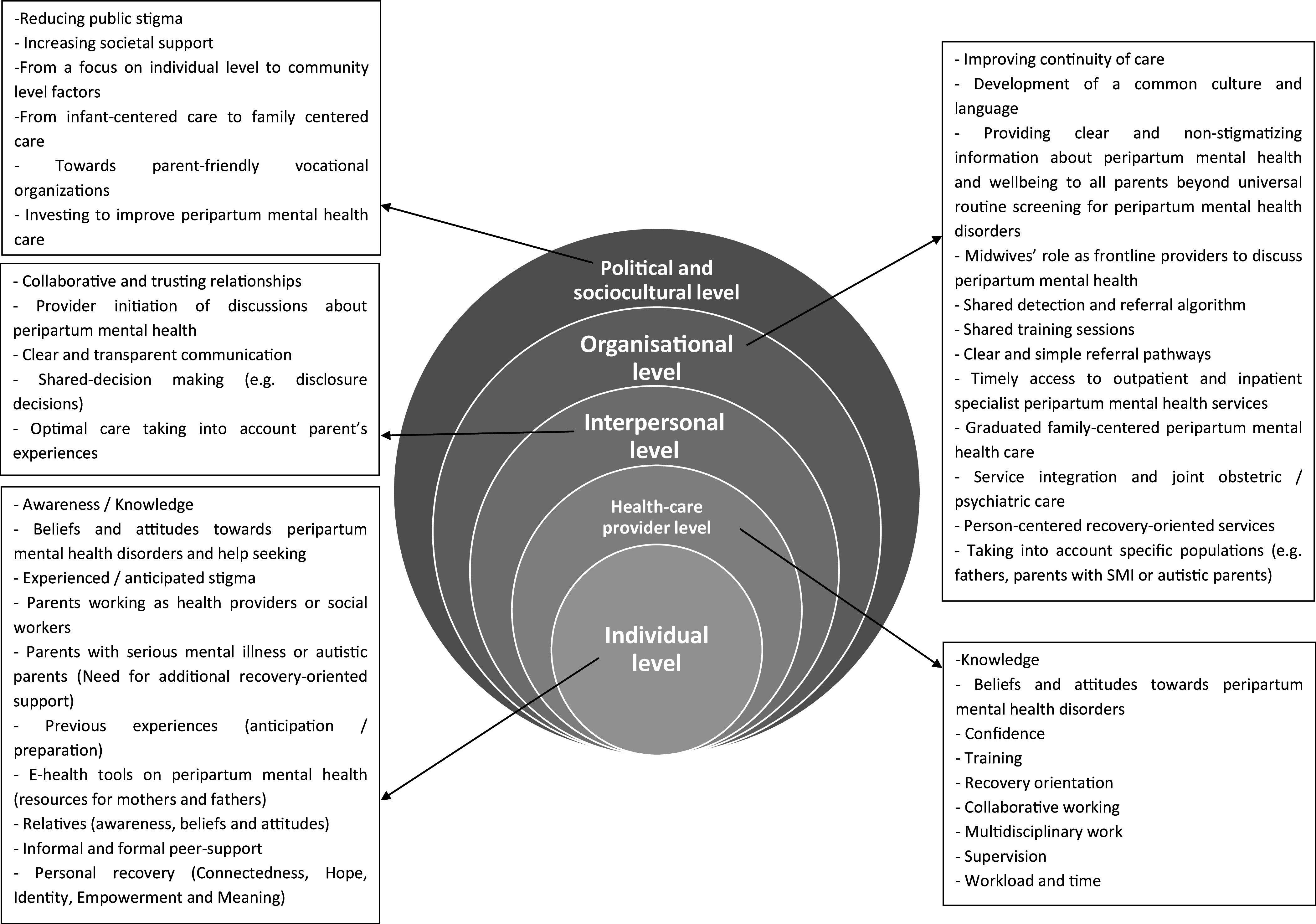
System-level representation of results.

The thematic analysis generated four super ordinate themes: (i) toward deep changes in the socio-cultural conception of the peripartum; (ii) challenging stigma; (iii) empowerment and personal recovery; and (iv) from a fragmented service provision to a graduated joint parent-baby care. We found no differences in the identified themes after removing nonparents in the PLEs group from the analyses except for subthemes related to preconception care (identified by an asterisk in Table 3 and Supplementary Tables 5 and 6). The results of the qualitative analysis are presented in Figures 1 and 2, Tables 2 and 3, and Supplementary Tables 3–6 (quotations supporting the themes and subthemes). The list of abbreviations is presented in Supplementary Table 7.

**Table 2.** Quotation supporting the themes “Toward deep changes in the socio-cultural conception of the peripartum” and “challenging stigma”

Toward deep changes in the socio-cultural conception of the peripartum
Increasing social support during the peripartum
*Mother 4*: “It would need a longer follow-up by midwives during the postpartum. […] In our society, you’re much more isolated, the mum is sometimes all alone all day long, that’s all, that’s it. […] that’s the worst, I think, to stay alone. Because if you’re supported, […] well, it would change a lot of things […] You would less worry on things that occur on a loop like that” *C&A Psychiatrist 3*: “Last weekend, she had all her family at home […] She had the impression to be the only one to care about what her 5-month little girl was living […] She spent the whole weekend being worried about her. And her husband was there, […] but he did not share at all the same concern, the same worry. At the end she felt totally left alone, abandoned”
Switching from a focus on individual level to community level factors
*Pediatrician 3*: “Maybe we could change how we look at the peripartum. Why that’s women who bear this” *Midwife 14*: “That’s something cultural in France. Political women who come back working after one week… So women believe that’s the real life”
Switching from infant-centered care to family centered care in the peripartum
*Midwife 25*: “Childcare nurses do a great job but, still, they’re focused on the baby. […] That’s not always easy to withdraw a little from the baby to take more interest in the mum. And, I think that us the midwives, we’re more on the mother’s side, and the job isn’t the same […] these depressions can stay unnoticed if the babies are going well” *Autistic woman 5*: “During pregnancy monitoring, I’d like that the mother would not be considered only as an uterus. Meaning child’s health, and also maternal health that would be taken into account” *Mother 3*: “That childcare nurse comes […], weights the babies. The babies are well, and I tell myself ‘well, once again they focused on them.’ […] And she asks, ‘how are you feeling?,’ I answer, ‘well, he doesn’t sleep, they are always in pain.’ And she says, ‘no, how you’re feeling?’ and actually, I have the impression that it’s been ages that no one asked me that question”
Toward parent-friendly vocational organizations
*Psychiatrist 2*: “A specific part on basically work environments, and what company leaders should know about perinatal psychiatry and consequently what vigilance they should apply in their company, etc” *Psychiatrist 3*: “Being parent and working, that’s not incompatible at all, but actually that needs an adaptation. Necessarily. There is inevitably an adaptation just so that you manage to not get burnout or overloaded” *Psychiatrist 4*: “Finally, the conclusion of all that, that’s it should almost be adaptable to each patient, even to each couple. For instance like in Scandinavian countries where they can share the parental leave, as they prefer, easily” *Psychiatrist 2*: “That’s not institutionalized. While that’s you who bears this” *Midwife 23*: “From one hand, maternity leave is very short and for some women it’s too long […] And paternal leave was also very short”
**Challenging stigma**
Reducing stigma
*Mother 1*: “To appear as someone weak. And I never liked that, and for me that was a weakness […] that was not my temperament” *Midwife 19*: “To not feel stigmatized when we want to orient them towards psychological support. That’s still classic to get the response that they’re not crazy” *Mother 1*: “People associate a lot depression with pills. […] So, well, reducing stigma, yes. […] People think a lot about psychiatry. And this word, ‘depression,’ that means hospital, that means hospitalization, and I think that’s scary” *Mother 4*: “I observe that it has already improved over 7 years. Anyway, it has evolved. And I see it, a lot of things happen. […] The speech is freed, even…”
Reducing feelings of shame and guilt
*Mother 1*: “Well, completely [the feeling of shame]! […] What’s funny, however, when speaking again about this, well, I realize that many people hadn’t noticed. […] I believed it could be seen through me and in fact not at all!”
High stigma for mothers who work as health providers or social workers
*Mother 1*: “I who am a nurse, I could see with my colleagues, if we care for depressive patients, there many who don’t understand it. And who tell, ‘no, but did you see, she doesn’t move during all day,’ or, ‘she doesn’t work, she could motivate herself.’ I who experienced it, now, well in fact that’s something hard. There is something stronger than not going out from your bed” *Mother 2*: “Yes, I think, of shame and then the fear of being judged by… what that could produce. I’m a social worker, do you realize what that can produce?”
Experienced and anticipated stigma in mothers with serious mental illness or autistic mothers
*Woman SMI 9*: “Well, I fear being labeled mother who is potentially dangerous for her child. I fear to be told, ‘she is potentially dangerous’” *Autistic mother 8*: “It will depend on the practitioner. If the fact of knowing I’m autistic, it renders less… they take it less seriously when I tell them there is that thing that isn’t ok, or that I tell symptoms and that’s not taken into account”
Stigma related to lack of knowledge in health providers
*Midwife 17*: “For me, suicidal risk is either immediately, but the big decompensation, meaning postpartum psychosis, the big thing, either it takes more time, a few months, a few years, and as a private practice midwife, I’ll have tendency to lose them on the follow-up level” *Psychologist 3*: “I don’t know if you can say postpartum depression in a psychotic patient who has a baby”
Providing clear, accessible and nonstigmatizing information about peripartum mental health and wellbeing to all parents beyond universal routine screening
*C&A psychiatrist 3*: “General public information too” *GP1*: “Maybe testimonies related to feelings, saying: ‘I am afraid of hurting my baby’? Well, some questions or ideas that you may have when you’re not well. ‘I’m tired of my baby,’ ‘I don’t like him,’ ‘I’m afraid not to like him,’ ‘I feel sad all the time, I just cry.’ […] the impression that you’re not alone, because in these moments you feel […] completely out of place. […] dads too” *Psychologist 6*: “I think there is still awareness to conduct […] of these mums too, through childbirth classes. Anyway, midwives talk now more about this. […] to make some pedagogy or training, to be able to say to mums, well, to make difference between baby blues and postpartum depression. […] there is all that prevention work that is interesting” *Midwife 26*: “During the postpartum care, […] I also tell them the signs” *Midwife 19*: “Our psychologist intervenes twice a week on half days and sees a large number of mothers in a systematic way. Meaning that we don’t tell the woman, ‘I find you a bit tired, would you like that to see the psychologist?,’ the psychologist just goes see her. Well, not at all intrusively, but rather systematically […] And very, very often that leads to […] real psychological interviews. While initially these mums wouldn’t necessarily saw her if there wasn’t that systematic contact” *Psychologist 2*: “It removes the taboo and something that is both mental and physical care […] I find that the way they’re seen by midwives too, that’s… […] if indeed they’re at ease like when talk about contraception, if they discuss mental disorders the same way, that’s accessible and that’s not taboo. That doesn’t bring uneasiness to them. But I think that for that they have to be at ease” *Mother 2*: “You can take the EPDS [Edinburgh Postpartum Depression Scale] on the 1000 days app, I think that’s a very good thing. […] I think that would have opened up some questions for me. It would have already posed something, it would have made what I was experiencing exist. I think that would have made sense” *Psychiatrist 2*: “Some realize the screening using EPDS during postpartum care, but the midwife who has to administer the questionnaire, she’ll not drop the scale in the patient’s room and leave, she has to contextualize things a bit, so to evoke that the point is to detect depression, so, what’s depression, what is its frequency, what are its symptoms, etc”
Training frontline providers on peripartum mental health disorders (knowledge/attitudes)
*Childcare nurse 2*: “Training is very important, we still have a lot to learn and see” *Psychiatrist 2*: “When a patient is pregnant, people who are untrained will tend to say, ‘oh, let’s stop or at least reduce psychotropic drugs in late pregnancy, that will be better for the baby’s arrival,’ whereas, in fact, data rather supports the opposite: there is a volume increase, doses should be increased at this period rather than reduced” *Pediatrician 2*: “There is a maternal depression scale made by the French Association for Ambulatory Pediatrics […] that is well constructed, that I never use. But anyway, I know that scales exist” *Mother 4*: “I went to my doctor at that moment who was desperate too, who didn’t know about mother-baby units, who tried to contact a local hospital. […] She managed to tell me, ‘I leave you a message. That’s not a psychologist that you need, that’s a psychiatrist, maybe your treatment should be adjusted,’ well the poor was a bit panicking. She left a message to a psychiatrist who called me back the day after”
Training frontline health providers (skills)
*Childcare nurse 1*: “That’s often complicated for us to make the mums adhere with the follow-up, or anyway to put in place some support […] that’s sometimes difficult for them to hear because they already feel very guilty about the care they can provide to their baby” *GP 1*: “That’s in the subtleness of not turning them on us, to avoid breaking the relationship too. And sometimes when we contact the psychiatry, that’s much more complicated” *Midwife 17*: “I have more difficulties in postpartum than in antenatal. […] in postpartum I really have this, ‘no, that’s not severe, that will pass’” *Midwife 24*: “That’s not so much that we don’t want to ask it or that we’re afraid to ask that question [suicide ideations], well, for me, that’s my romantic side, a bit naïve, that’s no [laughs]. Unless, […] the woman express it clearly and from that moment, we’ll put all in stage for the care” *Midwife 13*: “To be confronted to the patient’s distress, I think that puts us in a difficult position” *Pediatrician 1*: “There is that gap where you’re told, ‘we’d like care at discharge from maternity because this mum will need some support and not to be separated from her child,’ and suddenly, something terrible happens while we did not understand anything. Which renders for instance the concerning information very difficult in some situations because you tell yourself, ‘what will I start?”

**Table 3.** Quotation supporting the themes “Empowerment and personal recovery” and “From a fragmented service provision to a graduated joint parent-baby care”

Empowerment and personal recovery
Personal recovery
*Mother 2*: “During all that time, I had much grown up. I often say that having lived all that, I feel I went deep into myself, but I came much higher than I would ever have if I hadn’t lived all that” *Father 1*: “That was in particular [EM, the president of Maman Blues] who told me, ‘don’t worry, it will take weeks, months but there is always an end.’ And that’s small words that do good. […] even if that will not happen right now, you know there will be the end of the tunnel. […] She outlined the small steps that you don’t notice. […] Even in the first weeks, there is still some positive moments” *Mother 3*: “Actually, the fact of doing a series of video interviews gave me more strength each time because I was telling myself, ‘maybe that will not be this job, but that’s it I’m getting back on top, I’m coming back in the game’ [laughs], that was that in my head and, ‘I’ll manage to do it and you’ll understand what I’m capable of, I’m not just the mum of twins, I’m not just a wife, not just a daughter, a sister, I’m also myself. [silence] And I’m not just a victim’” *Mother 1*: “I don’t necessarily like playing mother […]. Well, I knew however what I didn’t like. And I was not ashamed to say it anymore [smiles]. I know I have the right to don’t like playing mother. Period” *Mother 2*: “Very quickly I really felt better in general… in my relationship with my son. […] But in a few weeks that story that he preferred his father was over. He only said, ‘mum, mum, mum.’ That really unblocked something. I felt much more competent. I had the impression to reinvest myself. That something that completely collapsed was rebuilding differently. And I found myself again” *Mother 3*: “And I tell myself, ‘if I generate something positive and if tell my story, that will make things grow and that’s how it will help other people too.’ And the association Maman Blues, they’re on Instagram and all Fridays they offer a mum’s testimony, anonymous or not, on ten slides. And in fact, I did it a couple of weeks ago. And the fact of reading it and viewing the comments… the true really nice comments below, that gives strength”
Informal or formal peer-support: repairs parent identity/facilitates recovery
*Mother 2*: “That’s mentoring that first saves women. […] I see on the forum how that can help women to repair themselves to just know they’re not the only ones going through this. And too, not the example, but knowing that other women went through that, well, that many found a way out and who are now helping others. That shows that there is an end to the tunnel, that it’s not definitive” *Mother 3*: “I tell myself, ‘actually, they won’t judge me’. And so I tell my story. […] And I feel not alone and I tell myself, ‘damn, there is an opening door, I have to take it’. And I hear many mums helping me. There is even one who offers to come help me with the bottles, etc., although she has two very young children. And I tell myself, ‘that’s it, you put the foot in a network … and that’s them who will bring you solutions in fact. Currently, that’s not health providers’”
Personal recovery in serious mental illness (SMI)
*Mother SMI 7*: “Well, when you’ve got an illness, in particular, a mental disorder, […] in the positive indicators, there is also, let’s say, the desire to have a normal life. So being able to have projects: getting married, having children, well… Starting a family” *Woman SMI 4*: “I rely a little on myself too. Because I think I asked myself enough the right question and now I feel ready. So I count on me too”^a^
Person-centered recovery-oriented peripartum health services
*Mother 3*: “And this job is to give back to the woman her place in the childbirth. Because that’s the woman who is at the core of the activity. Health providers are for me necessary electrons that gravitated around her, but the core is the mother” *Midwife 12*: “Just sitting down at the bed of a woman and simply listen what she has to say and free the speech, it allows settling down things a little. […] Also about bad experiences of childbirth, […] we sometimes aren’t on an equal footing: we experienced it well because we have a baby who is doing well, but this woman had a very bad experience of her childbirth. And to listen them, you realize that that’s already a lot” *Mother 2*: “I think that change comes from patients once they’re able to take hold of their experience and to transcend it” *Autistic woman 3*: “And maybe at the time of childbirth, that your wishes, let’s say, could be respected as much as possible […] with variations if there is an emergency or whatever and depending on the course of childbirth, but that there would be a guideline with the wishes that should be respected as much as possible” *Obstetrician 2*: “We try to take care of the patients globally, as well in terms of medical care, psychological care, lived experience and I find all that very interesting” *Mother 4*: “The thing is to know how to communicate without being scary. But I still think that us, the women, we’re entitled to the truth, to know, to be in the knowledge, and that when it happen to us, see, I better handled the 2nd time than the 1st” *Mother 2*: “I had a strong desire of a 2nd child and a 2nd childbirth too. I really desired to live things differently. […] So my pregnancy started very well. Let’s say that I was preparing my childbirth like you prepare a siege. […] Actually, I told myself that maybe I couldn’t do much on how that will happen in terms of context, but what I could change was how I would experience it”
**Switching from a fragmented service provision to a graduated joint parent-baby care**
Investing in peripartum mental health
*Psychiatrist 2*: “Peripartum care should not take place in adult psychiatry as well as in child and adolescent psychiatry. Really we should have dedicated places at maternities for perinatal psychiatry. And we see that the services offering that type of care are more effective”
Improving continuity of care
*Pediatrician 4*: “We don’t see those babies, but that’s true that our level, regarding hospitalized babies, I think there are really things that should be improved in the connection obstetrics/pediatrics” *Midwife 23*: “There is that Taskforce project […] the PMI [maternal and child protection] joined it. And in fact the goal […] was to create a care pathway for women who might already be identified but who could also be referred to us after a first consultation because the midwife would have detected warning signs […] there would be a midwife identified for the follow-up of psychologically vulnerable people during pregnancy” *Midwife 3*: “Psychologists and psychiatrists having pregnant patients shouldn’t hesitate to come to us. […] I have many psychiatrists calling me, saying, ‘Mrs. Whatshername is pregnant, I refer her to you.’ And that’s true that this trust is agreeable, and that works both ways in fact” *Psychologist 5*: “Patients should have access to available places of care, available working time […] be received without too much delay, being received at a pace that suits them”
Facilitate the development of a common culture and language
*Midwife 8*: “I think that what is important is that we could all speak the same language, so effectively assessments grids” *Pediatrician 2*: “There is probably a lack of some kind of common training” *GP1*: “the childcare nurse during the home visit […] the link with the general practitioner (GP). […] it requires knowing each other, saying things to each other, knowing each other’s place” *Midwife 5*: “In Valence there is a culture about this. There was a very involved psychiatrist, well, who left the area, but there was really joint work with the maternity team” *Midwife 21*: “Whereas suicide occurs finally weeks, months, or even in the first 1000 days, well, in the next 2 years, and when you are a hospital midwife, you will never be aware of it. And so that’s invisible for us. […] but that we could have some kind of feedback on whether there were perinatal deaths in the first two years. I don’t know how that could be possible, but to be aware”
Development of a graduated referral algorithm based on the needs of the parent–child triad and the degree of emergency
*C&A psychiatrist 1*: “The adaptability of care depending on patients” *GP1*: “When you get to a certain level of depression when the mum is very, very, very unwell, the idea is to separate her from her baby too. […] And to have no mother-baby unit to hospitalize, to work on the bonding despite everything. […] that’s something that always remains painful” *Obstetrician 2*: “Joint consultations with a psychiatrist and an obstetrician” *Midwife 11*: “Woman having psychiatric treatments, opiate substitute treatments and indeed we work very, very, very, well with the USAP [early support in perinatal care]”
Facilitating access to specialist peripartum mental health care (SPMHC)
*C&A psychiatrist 3*: “That needs some thinking, a little revolution of the systems. Some are a bit limited, too delimited, so that needs that we think about reorganizing care in a broader territorial fashion” *GP2*: “Having something reactive, and having the possibility that these mums could… when they know they would be able to see someone rapidly, that calms them down much” *Mother 2*: “And so I found out that there was a lot of resources in our area, including in our city, […] that the providers don’t know, except those who are at the core of the target. But that’s not necessarily them that the women will meet” *Psychiatrist 2*: “That should be how to give access to care and to whom to turn, that’s a fairly central element of that” *GP3*: “What do we do for referral after? […]That’s the problem. The more tools you have, the more you detect but after there is nothing behind. […] that’s currently the problem of my job” *Mother 3*: “a night when I could not fell asleep, I’m lying in bed with my phone and I type, ‘desperate mum, wishing to be dead.’ And I come to ‘Maman Blues Association.’ […] And I open the link and read and tell myself, ‘in fact, there are people to help me, I’ll hurry to contact them,’ and I send a message, it was like 2 a.m., saying that I had twins, I’m going really bad, that I need help and I don’t know towards whom turning myself. […] And there, they [Maman Blues] tell me, ‘listen, close by your town there is a CMP [medico-psychological center],’ and I don’t even know what it is, ‘and there is a parent-baby unit. You have to come see them’” *Midwife 12*: “Maybe support groups. Because being able to discuss with other mums who’re living the same thing and have difficulties too, that avoids stigma. And to say, well, I’m not alone. […] I don’t have to be ashamed, I don’t have to feel guilt”
Facilitating access to SPMHC for fathers
*Psychologist 8*: “We recently had a father who was hospitalized at full time. That was actually a first. […] her partner died one month after childbirth. […] there was all an orientation worked with a maternal center that was ready to receive that father and his baby. The answer of the Department on the funding was, ‘that father has just to return to work. He has better confide his baby, to place him and to return to work. That’s not his place to be with his baby.’ […] That was really surprising” *Father 1*: “Something that could work well, well that worked for me, […] that’s indeed testimonies. Or people who could be listening […] but anyway testimonies, maybe before, to realize that that does exist, that there are some people experiencing it, that we won’t all experience it in the same way, uh… I think that’s never hidden, that fathers can be affected, but that would be nice if they were more represented. […] That’s very often associated with the mum, and that would be nice hear some testimonies from fathers too. So testimonies for sure”
Women with SMI and autistic women specific needs and expectations
*Woman SMI 9*: “I mean, how can I balance my life? For example, take a typical day, how can I take care of myself while taking care of my child?” *Woman SMI 2*: “And maybe to have a follow-up also maybe more important than a person who doesn’t have bipolar disorder. […] that’s reassuring” *Woman SMI 1*: “A document that could allow thinking and analyzing my worries and my fears and on which I could then write what I would like to put in place. […] if we find ourselves in that situation, what would we do, which actions would we put in place”^a^ *Mother SMI 5*: “My fear was mainly about the medication. But I was lucky enough to have a very good psychiatrist who asked […] a psychiatrist trained in peripartum to have a treatment compatible with pregnancy and to have the less withdrawal syndrome as possible after childbirth”

a Theme identified only by nonparents.

### Toward deep changes in the socio-cultural conception of the peripartum

Changing socio-cultural conceptions of the peripartum to improve PMH was a theme running through all interviews or focus groups. Peripartum was described as a major life event with community-level implications (e.g., impact on the mother’s sense of personal identity, lifestyle, and social roles but also on the partner, the relatives and more broadly work environments). However, many participants felt that the community considered the peripartum mainly as a personal experience. While they acknowledged positive evolutions over the recent years, participants called for what they called a “cultural revolution” to improve PMH, that is, switching from a focus on individual level to community-level factors (e.g., switching from infant-centered care to family-centered care and adapting work environments to the needs of young parents).

### Challenging stigma

Challenging stigma was another theme running through all interviews or focus groups. Public stigma (e.g., depression as weakness of character and inability to provide adequate childcare), feelings of shame about seeking help and anticipated stigma from relatives and HPs (e.g., fear of not being taken seriously) were major barriers to detection and timely access to PMHC. Mothers working as HPs or social workers anticipated additional stigma related to the intersection of PMHD and their vocational status. Women with SMI and autistic women reported experienced stigma from HPs during maternity care and anticipated to be discriminated against in case of disclosure. Additional barriers were described for fathers with PMHD (e.g., feelings of shame and limited access to perinatal support services such as “maternal centers”). Suicide ideations or SMI were associated with stigmatizing beliefs in HPs, for example, perceived dangerousness for others. OPs reported to fear of being intrusive when opening discussions about PMH with parents without identified risk factors.

Challenging stigma meant to improve knowledge, beliefs, and attitudes toward PMHD and SMI in the general public and in HPs. PLEs and HPs recommended providing clear, accessible, and nonstigmatizing information about PMH and wellbeing to all parents during the perinatal period. Similarly, universal routine screening was seen as a way to reduce stigma and to facilitate provider initiation of discussions about PMH with all parents. Participants outlined the need for an adequate training of HPs on PMH and wellbeing – and not merely on PMHD.

### Empowerment and personal recovery

Parents with PMHD described personal recovery as a nonlinear self-broadening process aimed at living a meaningful life, a definition that corresponds to the CHIME framework proposed in SMI (connectedness, hope and optimism, identity, meaning, and empowerment; [18]). While HPs did not mention personal recovery or recovery-oriented practices, some OPs acknowledged the need for a shift from a medical conception of perinatal healthcare to a consumer-informed biopsychosocial perspective that considers the expertise of mothers. This included shared decision-making, for example, about disclosure of sensitive information to other HPs. Informal and formal peer support by PLEs played a central role in personal recovery for all participants. In addition to common themes identified by both parents and nonparents (e.g., being parent is a motivating factor for personal recovery), nonparents with SMI considered personal recovery as a facilitator in the decision-making process about starting a family.

### From a fragmented service provision to a graduated joint parent-baby care

To improve the organization of perinatal healthcare, HPs formulated several recommendations influential at different levels across the care pathway: society, organizational, interpersonal, provider, and individual. Society-level factors included investments to improve access to SPMHS providing optimal family-centered care (e.g., perinatal psychiatric services, distinct from adult and child and adolescent psychiatry that offer graduated joint parent-baby care).

Organizational factors included improving the continuity of care and developing a common culture and language between non-MHPs and MHPs. Participants – in particular midwives – formulated the following ideas: (i) shared training sessions on PMH; (ii) regular supervision and feedbacks from MHPs to non-MHPs on clinical situations; (iii) e-health tools for a clear, secured, reactive, and transparent exchange of information between HPs and for an up-to-date directory of the local resources; (iv) universal routine screening and the implementation of a shared detection and referral algorithm graduated according to the parents-baby triad’s needs and the degree of emergency; (v) clear referral pathways; and (vi) access to reactive outpatient and inpatient SPMHS. In addition to mother-baby units, integrated inpatient or outpatient perinatal health services with OPs and MHPs were described as a useful resource for optimal SPMHC.

Provider-level and interpersonal factors included training non-MHPs and nonspecialized MHP on PMH to improve their sense of confidence when caring for parents with PMHD. Other factors included clear and transparent communication between parents and HPs and between non-MHPs and MHPs. Among parent-level factors, informal peer support facilitated the access to PMHC. Other factors included raising awareness on PMHD in parents and relatives, support groups, e-health tools on PMH and additional support for mothers with SMI and autistic mothers, for example, attending to a dual set of needs or optimal service provision including preconception care.

## Discussion

Improving PMHC is a complex intervention that requires integrating the perspective of PLE, OPs, CHPs, and mental health providers [3]. To our knowledge, this qualitative study is one of the first including all these populations using a participatory research design.

### Inter-personal level and individual level factors

We found many interactions but also some degree of difference in the identified priorities between PLE (e.g., personal recovery and person-centered care) and HPs (e.g., common culture, improving inter-provider communication and risk management). This aligns with the emerging literature on personal recovery in PMHD that focused on the perspective of mothers [7, 8, 19]. Competing priorities between PLEs and HPs are also observed in the literature about recovery in SMI [20] and concerned in particular women with SMI, for example, anticipated challenges during the peripartum in PLEs versus risk management in HPs. This aligns with recent qualitative research comparing the experience of (future) parents with SMI and MHPs [21].

PLEs and some OPs acknowledged the need for a shift from a medical conception of perinatal healthcare to a person-centered perspective considering the expertise of parents. Beyond improving parental PMH [3, 22], horizontal and collaborative relationships, self-determination, empowerment, social connectedness, and peer support facilitated personal recovery in parents with PMHD. Howard et al. [23] showed improved mothers’ satisfaction after admission to a mother-baby unit compared with generic inpatient care, a finding that could be related to the provision of noncoercive specialist care. This suggests that recovery-oriented practices (i.e., strength-based person-centered approach supporting hope, empowerment, and goal-striving; [24]) could be relevant in perinatal health services and SPMHS.

While public stigma and anticipated stigma had negative effects on PMH, help–seeking, and engagement into the care pathway for all parents [3, 25], we found that this was particularly the case of four populations, that is, men with PMHD, non-MHPs or social workers with PMHD, women with SMI and autistic women (e.g., fear of being labeled as “dangerous,” “fragile,” or “bad mothers”; [2, 21]). This concurs with research on fathers with PMHD [26] and HPs with nonperinatal depression [27].

### Healthcare provider level factors

Aligning with Noonan et al. [28], stigma in HPs included the fear of being intrusive when discussing PMH with women without identified risk factors and the desire to protect them from “being labeled” by a referral to SPMHS. While non-MHPs reported more negative attitudes in case of suicide ideations or history of SMI, this aligning with previous research [29], several HPs expressed concerns about disclosing sensitive information with other HPs, this leading them to support shared decision-making (SDM) in this context. This concurs with the positive effects of SDM in treatment options on women’ experiences of PMHC [3] and decisions about disclosure in SMI [25]. Training sessions should target the interaction between knowledge and beliefs and attitudes (i.e., stigma [25]) and include content about interviewing skills, distress management, parents with SMI and autistic parents [1, 2].

### Political and socio-cultural level factors

Extending the results of previous research that focused on organizational, interpersonal, HP, or individual levels [3], we found that society-level factors, for example, switching from infant-centered care to family-centered care and promoting parent-friendly vocational organizations, are determinant to improve PMHC. This aligns with the benefits of paternity leave uptake on paternal PMH [30] and Wilkinson [31] proposition of a research agenda to examine the interaction of maternal PMH and employment. Consistently with other high-income countries (e.g., the UK; [3]), participants called for an adequately funded national public policy to address the gaps in PMHC (e.g., access to reactive perinatal psychiatric services [10]).

### Organization-level factors

Participants identified factors influential at the organizational level (e.g., clear referral pathways) that concur with Webb et al. [3]. Participants called for a universal promotion of PMH for all parents extending beyond universal screening, this aligning with studies reporting that parental PMH should be integrated into standard perinatal health care [22, 32]. Other recommendations included offering more resources for fathers with PMH and additional recovery-oriented care to mothers with SMI or autistic mothers, this aligning with recent qualitative research [21, 26]. Patient representative associations were determinant at different levels across the care pathway (e.g., raising awareness; facilitating access and referral to SPMHC; facilitating personal recovery).

### Limitations

There are limitations. First, our sample was self-selecting (i.e., persons interested in improving PMHC) and cannot be considered as representative of the experience of all stakeholders involved in PMHC. However, the large size (*n* = 84), the diversity of the sample (i.e., realization in five distinct locations, the inclusion of various PLEs [including women with SMI and autistic women], and inclusion of HPs with diverse backgrounds and practices working in urban, semi-urban and rural areas) and the use of three triangulation methods (i.e., investigator, methodological and data triangulation) are considerable strengths. Similarly, the proportion of women was high in all groups (95% in the HP group and 95.8% in the PLEs group) and most HPs worked in public hospitals (56.7%), thereby reducing the generalizability of our findings to men and private practice providers. Of 24 PLE of PMHD or SMI, ¼ worked as HPs or social workers. Given the experience of this at-risk population remains under-investigated, this could be a strength. Second, given nonparents may have limited lived experience of perinatal healthcare apart from preconception care, the high proportion of nonparents (45.8%) included in the PLEs group could be a limitation. However, we found no difference in the identified themes after removing these participants from the qualitative analysis – except for subthemes related to preconception care (e.g., personal recovery as a facilitator in decision-making about starting a family and joint crisis plans). Third, many individual interviews or focus groups were conducted online because of the pandemic context, which could have affected the quality of data collection. However, in-person and online focus groups yielded comparable themes and online discussions facilitated sharing of in-depth personal stories and discussion of sensitive topics in a recent study [33]. Fourth, researchers’ own position, views, and opinions can influence the research process [13]. Adopting a participatory research design and a reflexive position from the inception of the project may have addressed this limitation. Fifth, we did not involve managers from public hospitals or local/regional public healthcare agencies or politicians in this study, which is a limitation given the role of these stakeholders in decision-making about healthcare.

Overall, this analysis generated novel insights into how to improve PMHC for all users including those with SMI or autism. These include a community focus on PMH, family-centered care, a better integration of mental health and perinatal health care and recovery-oriented practices.

## Supporting information

Dubreucq et al. supplementary material 1 Dubreucq et al. supplementary material

Dubreucq et al. supplementary material 2 Dubreucq et al. supplementary material

Dubreucq et al. supplementary material 3 Dubreucq et al. supplementary material

Dubreucq et al. supplementary material 4 Dubreucq et al. supplementary material

Dubreucq et al. supplementary material 5 Dubreucq et al. supplementary material

Dubreucq et al. supplementary material 6 Dubreucq et al. supplementary material

Dubreucq et al. supplementary material 7 Dubreucq et al. supplementary material

## Supplementary material

The supplementary material for this article can be found at https://doi.org/10.1192/j.eurpsy.2023.2464.
