# Supplementary material for: Toward recovery-oriented perinatal healthcare: A participatory qualitative exploration of persons with lived experience and health providers’ views and experiences
Source: Eur Psychiatry. 2023 Oct 20;66(1):e86. doi: 10.1192/j.eurpsy.2023.2464 (PMC10964275; doi:10.1192/j.eurpsy.2023.2464)
Supplement: Dubreucq et al. supplementary material 1 — Dubreucq et al. supplementary material [file S0924933823024641sup001.docx]

**Supplementary Table 1. Evidences before this study**

We searched PubMed, Google Scholar and Embase for articles published in English and in French between Jan, 1, 2000 and May, 22, 2023, using the search terms “improvement”, “perinatal” (peripartum, antenatal, postnatal), “mental health care”, “participatory” (collaborative, coproduction), and “recovery” (recovery-oriented, person-centered, treatment preferences). We also screened the reference list of five systematic reviews on topics related to improvement of peripartum mental health care (barriers and facilitators, person-centered care and women’ experiences). The search yielded 75 articles that were applicable to our study objective. While many qualitative studies explored this issue, there remain some limitations to the current body of research. First, most studies were conducted in the United States or in the United Kingdom and did not include the perspective of all stakeholders (e.g. no comparison between the perspectives of persons with lived experience and those of peripartum health / mental health providers). Second, while optimal service provision refers to person-centered care co-produced with persons with lived experience, most studies did not involve researchers with lived experience nor used a participatory design. Third, the literature on personal recovery and recovery-oriented care in the peripartum remains scarce. Fourth, there is limited research on the experiences fathers with peripartum mental health disorders. Fifth, most studies excluded women with serious mental illness (SMI) and did not cover preconception care. Sixth, the experience, views and needs of autistic (future) mothers remains under-investigated.
