# Supplementary material for: Toward recovery-oriented perinatal healthcare: A participatory qualitative exploration of persons with lived experience and health providers’ views and experiences
Source: Eur Psychiatry. 2023 Oct 20;66(1):e86. doi: 10.1192/j.eurpsy.2023.2464 (PMC10964275; doi:10.1192/j.eurpsy.2023.2464)
Supplement: Dubreucq et al. supplementary material 2 — Dubreucq et al. supplementary material [file S0924933823024641sup002.docx]

**Supplementary Table 2. Semi-structured interview**

General information (health providers):

-Gender:

-Age :

-Profession:

-Duration of professional experience:

-Type of practice (hospital, private practice, territorial practice):

-Did you care for a patient with peripartum depression within the 3 last months?

How do you feel when caring for patients with peripartum depression?

Not comfortable at all Rather uncomfortable Neutral Comfortable Very comfortable

General information (patients):

-Gender:

-Age:

-Education:

-Marital status

-Number of children:

-Psychiatric diagnosis:

Semi-structured interview :

1. With respect to a recent - positive or negative - experience, could you tell us what happened? What challenges did you face or anticipate and what helped you?

Subsidiary question

1. What would you expect from e-health tools (e.g. a mobile application) dedicated to parents and health providers?
