# Supplementary material for: Toward recovery-oriented perinatal healthcare: A participatory qualitative exploration of persons with lived experience and health providers’ views and experiences
Source: Eur Psychiatry. 2023 Oct 20;66(1):e86. doi: 10.1192/j.eurpsy.2023.2464 (PMC10964275; doi:10.1192/j.eurpsy.2023.2464)
Supplement: Dubreucq et al. supplementary material 3 — Dubreucq et al. supplementary material [file S0924933823024641sup003.doc]

Supplementary Table 3: Quotation supporting the theme "Towards deep changes in the socio-cultural conception of the peripartum" (**bold** figure in Table 2)

| **Towards deep changes in the socio-cultural conception of the peripartum** | |
| --- | --- |
| **Increasing social support during the peripartum**  Support by health providers (HPs) and social workers  Partner and family support | **Mother 4 [39 years old]:** “Well, I read a lot of things on the subject, so I’m inevitably a bit oriented, but that’s true that to detect them, it would imply that midwives could come […], it’s not necessarily the first weeks, look A. it was at 5 months. But **it would need a longer follow-up by midwives during the postpartum**. Indeed, the first appointments, I don’t know the precise legal context anymore, but the first appointments on the first two weeks, the weight monitoring, ok, baby ok, but well, I don’t know, an appointment at 2 months postpartum to punctuate this in time. For that matter, I don’t know if you have read “this is my postpartum”, well she had a lot of good ideas. […] there are countries where it’s much more developed. Doulas, isn’t it? Mums who come to help us. **In our society, you’re much more isolated, the mum is sometimes all alone all day long, that’s all, that’s it.** Unless there is another child, but if it’s a young child it’s even more difficult. I think we miss […] being supported during the day, **that’s the worst, I think, to stay alone.** **Because if you’re supported,** it’s better by a woman but well could also be a man, I’m open to this. But **well, it would change a lot of things** if you had some people who stay home longer and more durably. **You would less worry on things that occur on a loop like that.”**  **Mother 4:** “I think mums should be offered more help in the first months after childbirth. To avoid finding yourself waiting all alone. I remember I waited […] the next breastfeed, but at the same time I was telling myself “it comes back too soon, I don’t have the time to do anything”, but actually I did nothing, so it did not make any sense”  **Midwife 5 [female, 58 years old]:** “mothers often come back home and find themselves really lonely. And it’s a complicated period because they end up with a newborn, with many worries, questions. […] We don’t listen them enough, they don’t really know to whom talk about this […] Getting back home is sometimes complicated and there isn’t enough listening and help at this period. […] a visit by a private practice midwife, well it allows […] to monitor these women a little longer, but some years ago they had only the pediatrician at 1 month postpartum. They eventually saw no one in the meantime.”  **Mother 2 [36 years old]:** “it was very hard, but at the same time I felt I built around this 2^nd^ pregnancy a sort of village**,** that everyone got organized to avoid it to be that way. B offered to continue childbirth preparation online, C. called me several times on evenings to chat with her private mobile phone. My obstetrician […] she gave me her private mobile number. Something actually happened, there was a village of women around me**."**  **Mother 1 [33 years old]:** “that’s something very challenging […] to get home all on your own, rather shortly after maternity discharge”  **Obstetrician 1 [female, 62 years old]:** “We should really reinforce the postpartum […] prepare the immediate postpartum: private practice midwife, PMI [maternal and child protection] or home treatment. I wish it could last longer in postpartum, that the peripartum network could continue in postpartum”  **Midwife 13 [female, 40 years old]:** “I build on the issue of society and this cultural question […] this period is tiring and that, but we do not give women the resources to rest”  **Midwife 13:** “Well, there are some we identify, return visiting and then refer to maternal and child protection, but not all of them. […] Unions claim a postpartum interview that would allow to settle down, to review things together. […] That we could also offer groups, small groups of mums, those who attended together to childbirth classes so that they can meet again. And share and break a bit this isolation. […] that’s important they receive the most possible support”.  **Woman with serious mental illness (SMI) 2 [22 years old]:** “After the pregnancy, well I tell myself I’ll need some people, I’ll need some help. I’ll need anyway to know who and when call, I’ll need some safety, even if I never call and that everything is going very well, fine. But I need to know that some people are available. Honestly, in a possibly utopian world, I’d need to have someone available day and night (laughs). But, well, I think anyway that keeping the network a bit longer than someone who hasn’t bipolar disorder, although I think some people without bipolar disorder would really need it, well, I think that would reassure a lot”.  **Psychiatrist 3 [female, 32 years old]:** “the day you become parent, you understand what being alone means. […] And that’s weird. Well, that’s weird to tell. But this is something that comes out a lot during consultations.”  **Autistic mother 10 [37years old]**: “Well, having someone sometimes to look after my child, it would have helped much. Well, I mean someone I trust and to whom I can tell eyes wide shut “this afternoon, you look after him, I’ll rest”, that would have been fine”.  **Pediatrician 3 [female, 47 years old]:** "on postnatal care by midwives at home, I still find that it contains well the parents. The fact of seeing a health provider quickly after discharge, the fact that the baby is weighed, [...] there is someone coming and listening to them. That’s done at home, which is rather adapted to the neonatal period, because that’s true that anyway parents are tired, [...] that's still rather positive for the parents."  **C&A psychiatrist 3 [male, 49 years old]:** “she had a real anxious depression on a rather obsessive personality. She was going better and **last weekend, she had all her family at home** […] And she went out this exhausted, and she really thought about killing herself. She said she was willing to jump through the window. […] **She had the impression to be the only one to care about what her 5-month little girl was living […] She spent the whole weekend being worried about her. And her husband was there, he pushed the pushchair, but he did not share at all the same concern, the same worry. At the end she felt totally left alone, abandoned**”.  **Psychiatrist 3 [female, 32 years old]:** “Or that you makes a link between depression and support, relatives, in fact. And that’s very obvious. […] I had more than once the feeling I was prescribing an antidepressant telling myself, “in fact, in itself, I’m not sure that’s the solution” […] but in fact, I get by as best I can and, well, the reality. I’m not going to grab the partner by the scruff of the neck and tell him what he has to do.”  **Psychiatrist 2 [female, 34 years old]:** “there are a lot of studies showing the importance of father support, of marital satisfaction, this is quite well described. But that’s true I can’t think of studies that investigated father’s investment, and beyond investment of the baby, the quite dynamic role the partner can have in daily organization.”  **Midwife 13:** “And I think the month of paternity leave is a huge progress (laughs). It should be a bit a little more longer, but that’s a huge progress […] if it’s correctly taken, if the partner invests this period too and takes it really for the family and the household, obviously”  **Psychiatrist 2:** “I think that’s anyway also an issue in social support and in family support, that is to say brought to women, which is something obviously central. That’s it. That’s slightly parallel to your question, but I think we can anyway make the connection with the place of this extended paternity leave”  **Psychologist 8 [female, 30 years old]**: “we intend to open parental rooms, and in particular the fact that there would also be the longer paternity leave could allow easier joint parents-baby hospitalizations”  **Mother 4:** “well at work my manager told […] the team I wasn’t well, that they should leave me alone a little (laughs). And that was very nice. And so, well, I anyway managed to work but I think I was slower. Surely, that helped, I had kindness around me. In my family too. […] My mother, who was still working, she went a weekend to see me. […] My stepmother came at noon to prepare lunch because at one point I was not eating anymore […] I had a friend who came by to support me. So, well, I did not feel like seeing a lot of people, but like this at small doses it was ok.”  **Psychiatrist 2**: “the extended paternity leave […] because that, that’s indeed a small revolution”  **Mother 4:** “Well, I don’t know what my parents-in-law thought about my hospitalization […] That was my things, my choices, my decisions, anyway my partner fully supported me, that’s even him who told them. So I did not care, yes and no, I did not much have to tell them myself, saying, “I’m going to be hospitalized”.” |
| **Switching from a focus on individual level to community level factors**  Changes in the conception of peripartum (individual vs. community responsibility; responsibility incumbent on mothers; period of increased vulnerability)  Sanitary crisis: a revelator of the responsibility incumbent to young parents | **Pediatrician 3:** “**Maybe we could change how we look at the peripartum. Why that’s women who bear this**”  **Midwife 14 [female, 45 years old]:** “**I thing that’s something cultural in France. Political women who come back working after one week… So women believe that’s the real life […] And you’re telling yourself “but I’m just a loser”.”**  **Mother 1:** “I think there is a pressure today, on the mum. And in particular this role, being perfect, to take perfect care of her house, to succeed at breastfeeding, to have quiet children […] I found that huge. And I thought I could succeed. That I has the shoulders for, well, I put myself a huge pressure”  **Midwife 13: “**I think things begin to change because we see books come out, such as “the 4^th^ trimester”, this shows there is a small evolution. But in fact, I think we do not prepare women enough because this period will be particular. And they expect giving birth and return to their former life. While, actually no, there are still a lot of ongoing physical and psychological changes”  **Mother 3 [35 years old]:** “To involve fathers more. […] He can stay the 1^st^ night or sometimes not. And after, the woman has actually to deal with everything. And that, that’s nor normal.”  **Mother 3:** “In the room, for instance, when he was there with me, she looks only at me. “Did you gave them the bottle today?”. She looks only at me. “How much they took?”. Actually, he’s there too, I don’t see why I’m the only one who has to answer to these questions. […] I have the impression to be at the university doing practical work with my partner, and that we had a bad grade and that the teacher comes to see only me actually, because my partner isn’t worth more than that in the group. And so, he did not feel involved, so for taking his role, well, he did not feel helped. […] There are some fathers who wait someone to give them their role. And the role is quickly filled by the mother who has to deal with everything and that’s not a good situation, I think”.  **Midwife 13:** “And I think the month of paternity leave is a huge progress (laughs). It should be a bit a little more longer, but that’s a huge progress […] if it’s correctly taken, if the partner invests this period too and takes it really for the family and the household, obviously”  **Psychiatrist 2:** “there are a lot of studies showing the importance of father support, of marital satisfaction, this is quite well described. But that’s true I can’t think of studies that investigated father’s investment, and beyond investment of the baby, the quite dynamic role the partner can have in daily organization.”  **Psychiatrist 2:** “We're personally just going out a family gastroenteritis, next week after the 4-day weekend imposed by the school and daycare, there is a national strike, so, the daycare is closed on Monday and Tuesday, so when can you work when you’re parent in these conditions? Well, it’s actually a challenge.”  **Midwife 26 [female, 52 years old]:** “couples coming with demands on their role of parents that are extreme. […] They have objectives for taking care of their child […] we see them get exhausted in the 1^st^ day of postpartum care.” (p4)  **Midwife 16 [female, 50 years old]:** “A lot of women idealize this period of childbirth and what comes next. That’s all beautiful, all rose […] they’re not been told that some things will be put in parenthesis, that the nights will be difficult.”  **Psychologist 5:** “before that, often for some […] there was anyway still to bear the idea that it wouldn’t be easy. […] they cling a little to some ideals, transmitted by the family or transmitted in the business sector too”  **Mother 2:** “and with a very mothering mother, anyway who fulfilled herself in her motherhood. So that seemed easy as pie to me. Let’s say that, to me, it was the normal continuity of a woman’s life to have children. I did not really ask myself any questions.”  **Mother 2:** “I did not feel the need for surrounding myself because for me that was hyper positive to have a baby, and that was not an ordeal.”  **Mother 3:** “And in addition to this, before becoming pregnant, I was telling myself, “but being pregnant, that’s not a disease”. And I saw so may pregnant women conduct their lives as they weren’t pregnant, well, classically, I told myself “that’s great, I’ll do the same”. And actually, I got to a zone I had not anticipated at all.”  **Psychiatrist 3:** “that’s not that once they come to us that’s done, but actually, the thing is to manage to come to us. And in fact coming to us is difficult. […] In fact, the idea is to coming to the psychiatrist and in that story that, well, you’re pregnant, that normally that was the best moment of your life, that everything is going well… that socially there is no problem. And actually that’s totally wrong!”  **Mother 3:** “Well, actually, we should stop idealizing pregnancy.”  **Mother 3:** “And he says, “well, I’m making your discharge”. And at this point (smiles), I secretly tell myself “I’ll come back home before them. Great!” […] He tells me, […] “you have to physically stay here day and night, because they’re not discharged yet”. And (waves arm), I tell myself, “damn. I actually have to stay in this hospital with them, while I don’t want to at all, I just want to go home”. […] And I ask, “why have I to stay?”. And he answers, “well, you’re the mother, you’re responsible of them, so you have to stay”.  **Pediatrician 3:** “I find that when we pass scales, e.g. of depression, we put a diagnosis on the mother. And just that, that’s a charge. That means that the mother isn’t going well. But is the father unwell? Is the family supportive? Is the family dysfunctional? Couldn’t we put this too? […] but finally why the mother is unwell? And I think that it makes them bear some guilt too. […] putting a diagnosis of depression only on the mother, I find that sometimes it burdens her even more”  **Psychiatrist 3:** “Or that you make a link between depression and support, relatives, in fact. And that’s very obvious. […] I had more than once the feeling I was prescribing an antidepressant telling myself, “in fact, in itself, I’m not sure that’s the solution” […] but in fact, I get by as best I can and, well, the reality. I’m not going to grab the partner by the scruff of the neck and tell him what he has to do.”  **Psychiatrist 4 [female, 35 years old]**: “it’s managing to discuss the contours of what we call peripartum depression. First, that’s not depression, that’s too diverse (psychologist 8 nods). […] The people I see know, who really have anxious and depressive symptoms, I have the impression the common feature for consulting is a trouble in parenthood. That means that’s the burden, […] meaning, “I have the impression that it falls only on me”. […] Women who live the feeling that the survival of their baby depend on them. And whatever the relatives who are very nice, kind, they have partners who really want to do what it takes, but they nevertheless feel extremely lonely in the preoccupation concerning their baby.”  **Psychiatrist 2:** “the sanitary crisis put the finger on that, anyway the difficulty for parents with children who had to do school at home, even if it was very delimited. […] it has highlighted that being parent was very complex at this period, and not only the first years of life”  **Mother 4:** “we were coming out from lockdown, the 1^st^, the real one. […] We were working from home, with the girls, the partner… this was a little crazy organization. So when I got pregnant, it was the moment when we could not, a little, we could not breathe anymore, we argued a bit with the girls (laughs), well, it was sometimes tense, it was really not the moment.” |
| **Switching from infant-centered care to family centered care in the peripartum**  Development of coordinated care by midwives trained in perinatal mental health  mothers’ preference to talk with a midwife  Midwives: key providers to improve the continuity of care between pregnancy and the postpartum  Switching from infant-centered care to family centered care during pregnancy  Switching from infant-centered care to family centered care during the postpartum period | **Midwife 25 [female, 60 years old]:** “**childcare nurses do a great job but, still, they’re focused on the baby. […] That’s not always easy to withdraw a little from the baby to take more interest in the mum. And, I think that us the midwives, we’re more on the mother’s side, and the job isn’t the same and for us, seeing again the mums in a second phase could maybe be interesting. Because that’s true that these depressions can stay unnoticed if the babies are going well.**”  **General practitioner (GP) 1 [female, 58 years old]: "**So at the PMI I work with midwives in antenatal, in postnatal midwives intervene maybe on 1 or 2 visits but not more. After that, we work a lot with childcare nurses who go at home.  **Midwife 25**: "colleagues in private practice see mothers more for perineal reeducation, for the gynecological side and that allows [...] to see again, discern and detect depressions that would set in a little later. It's true that I don't necessarily see them after the first month. So we lose them. So the childcare nurse colleagues try to take over, but we don't have the same job, and there are mothers they don't necessarily see them again if everything goes well in the first month. So there isn’t always a PMI follow-up in the long term."  **Mother 1:** “my midwife made me doing perineal reeducation even though I did not need it, extra, to make me go out”  **Obstetrician 1:** "the care network, the midwifery network, that’s a long story in Valence, and (...) our child psychiatrist (...) waved that work on the importance of a midwifery network, trained the providers to screening, and did all this underground work."  **Mother1:** "they made me see the psychiatrist... I didn't really like it I'm not fond of psychiatrists. [...] I preferred to consult my midwife that suited me very well. She acted as my psychologist, that was perfect"  **Psychologist 2 [female, 40 years old]:** “I was telling myself, on shame, probably that the way frontline midwives discuss this or intervene… they make it more accessible and, so, that’s something easier for patients to confide themselves.”  **Psychiatrist 1 [female, 43 years old]:** “To sum up, a good midwife that’s very nice… We know there are midwives who have this psychiatric listening. […] And sometimes when there is this handover and for example, when the midwife comes with the person to the consultation of the psychiatrist, things happen in a way that is far less painful, with much more trust”  **Midwife 3 [female, 42 years old]: "**That’s good to rely on a care network, it has to exist, but I find that in Valence we are lucky to have that position of midwife case manager, which allows [...] to detect vulnerabilities, precariousness, psychological distress, patients using drugs, and then in a second step we can call them, orient them and that creates trust."  **Psychologist 6 [female, 39 years old]:** "For me they really are frontline providers to detect. With pediatricians and GPs, but I find that the midwives"  **Midwife 4 [female, 34 years old]:** **"**as midwives we detect a lot patients and we can really propose the right team and the right follow-up**.**"  **Pediatrician 2 [male, 74 years old]:** "Apparently, every woman has a referent midwife when leaving the maternity I didn't know that existed. But at the limit, maybe she’s the most easily identifiable provider, and to me, there are still some midwives. [...] GPs aren’t there anymore, pediatricians even less."  **Mother 4:** “I think that in maternities, well not only in maternities but since the 1^st^ consultations during pregnancies, once you know that the baby is kept, that’s midwives who should do this work during pregnancy follow-up, to talk, to evoke difficulties… well, not the medical difficulties of childbirth, well, there’s a lot too, but at least, you can’t see all, the psychological difficulties in the first months postpartum. But I think that should be since the first consultations.”  **Midwife 12:** “I always try to tell them that if they don’t feel good in postpartum […], they shouldn’t hesitate to contact providers […] a psychologist, […] their GP or the private practice midwife […], or the PMI”  **Midwife 17 [female, 36 years old]:** "there is the perineal reeducation. And that, as it is often done with midwives, well we can intervene. We can also use this tool to say "well, you don't have an appointment with your psychiatrist right away, but you still have a health provider who is there and there you go, you talk to her about the baby, and she knows a bit about the subject".  **Father 1 [34 years old]:** "Finally the midwife, that was just the beginning, really the primer.”  **Autistic woman 5 [33 years old]**: “**during pregnancy monitoring, I’d like that the mother would not be considered only as an uterus. Meaning child’s health, and also maternal health that would be taken into account**”  **Woman SMI 2:** "During pregnancy, we have more providers available, and afterwards, that's when there are fewer things. Even if there are some, but still fewer. We're more often alone at home, what."  **Mother 3:** “thinking back on the ultrasounds every 15 days, well, it did good, because I was the focus of the consultation and because before ultrasounds, they asked me, “how are you feeling? Are you eating well? Are you sleeping well?”. And I told myself, “I’m not only the person who bears”. […] I knew it was also in the interest of the babies but they didn’t ask, “how are the babies?”. And that was later during the consultation that they asked “do you feel them moving?””  **Mother 3:** “to ask again providers just being themselves. […] they’re human beings like others. That’s just that they’re put in a context, in a flow that is not natural. And so they endorse masks that indeed render them inhumane.”  **Mother 3:** “**That childcare nurse comes […], weights the babies. The babies are well, and I tell myself “well, once again they focused on them”.** So we put them to sleep. And at this point… I tell myself, “well, she’s going to leave”. And actually, she asks, “can I sit?” (Silence). And I, in front of her, I was longing for this and I answered, “yes, yes”. And she sat and looked at my husband and asked, “could you leave?” […] **And she asks, “how are you feeling?”, I answer, “well, he doesn't sleep, they are always in pain”. And she says, “no, how you’re feeling?” and actually, I have the impression that it’s been ages that no one asked me that question**. And I don’t know where to start. And I begin to tell her all that’s going out”  **Mother 2:** “our health system in France results in women meeting some providers. That’s not a system where a woman experiencing her postpartum depression, experience it alone, without any interaction. […] Which is pretty good news for me because that means there are multiple occasions for these providers to grasp this question. Except that, at first glance, these occasions are actually not oriented towards these questions. You’ve the postpartum visit, in which you’re told about your episiotomy scar, but finally we could discuss something else, you’ve all the visits to the pediatrician, all the lactation consultations. There are many occasions for women to connect with providers.”  **Mother 4:** "the 1^st^ time, I was angry, well angry, against the system, because I didn’t know and I told myself, “well, I spent 3 weeks in a kangaroo unit with a premature pregnancy so a risk factor for depression, and not once in the 3 weeks although I always ran into team members, someone told me, “anyway, you should know that if you’re not going well after returning home…”. I would have liked that someone told me about it. [...] I think that in maternities, well, not only in maternities, but from the first pregnancy appointments, once you know that the baby is kept, that should be the midwives who do that job during the pregnancy follow-up, talking about it, evoking difficulties… well, we don’t speak about the medical difficulties of childbirth, well, there are plenty of them too, but at least, psychological difficulties in the 1^st^ months postpartum."  **Pediatrician 4 [female, 48 years old]:** "we don't see the babies very often afterwards, and [seeing the mums] that's not our number one job. Anyway, that would be good if they were systematically seen again on the obstetrical level."  **Psychologist 4 [female, 38 years old]:** “one of these mums who had a depression that remained unnoticed because she was doing all that needed to be done for her baby but in the inside, it was a disaster”  **Mother 3**: “And there was also this double hospitalization I was not at all aware of and I did not know someone could say to the mum “from now, we do not take care of you anymore, you’re physically there, but you stay for them”. This, really, I still struggle to understand it now.”  **Mother 3:** “from the moment I’m administratively discharged, no one cares for me physically, except for removing staples.”  **Mother 1:** “I think there are a lot of mums, before suffering from depression… because I really think that sometimes this depression is caused by all the questions you ask to yourself, and sometimes this tiredness you produce yourself”  **Father 1:** “Well, the care had a big role. We were taken care of by… it was very simple 1-h sessions, and it allowed discussions, and above all, she did not gave the floor only to me. To my partner too, and for that, that was not bad. Because without realizing it, I think it was rather demanding for her, even if she is rather strong”  **Father 1:** “It was a psychiatrist. […] What helps there is that there is an environment a bit for parents but also for children, meaning there is not a lot of white cloaks, you see what I mean?”  **Nurse 2 [female, 50 years old]:** “I build on access of the child through pediatric services. We often receive overwhelmed mothers because they’re not consulting for themselves, but for the baby. And very often with our colleagues in pediatrics, child who are there for crying and who finally reveal maternal depression”  **GP3 [female, 52 years old]**:"They [midwives] warn when the baby is not well, but I have never been contacted when the mum was not well."  **Midwife 25:** "for us, seeing again the mums in a second phase could maybe be interesting. Because that’s true that these depressions can stay unnoticed if the babies are going well. […] I don’t know if this postpartum interview could allow to see the mums a little more and in a little more systematically"  **Midwife 1 [female, 51 years old]:** “we often ask ourselves about postpartum depression during the next pregnancy. […] There is a period of emptiness for women in postpartum between the end of the private practice midwife’s care and the 1^st^ appointment with the GP. There is a moment when we’ve the impression they have no monitoring. Perineal reeducation is over, they don’t see their private practice midwife anymore and they’ve contraception, they return to work, they haven’t seen their GP yet and thus there is no one at this moment and that’s when it happens. And so that poses the question of the continuity of care.”  **Mother SMI 8 [40 years old]**: “the lack of care. I found it hard to be abandoned […] after childbirth, that I didn’t have a doctor anymore and all that. I find the follow-up important, and I believe I wouldn’t like do it again with such a precarious situation […]. I would not like to be abandoned. Actually, the part on maternal healthcare is important. […] I prefer to be cared for.”  **Pediatrician 4:** "These mums who had emergency caesarean sections, finally, they don't necessarily see the psychiatrist systematically, [...] after 5 days that's not so bad, hop you go home and then you'll see, if you're lucky, in a month - a month and a half the obstetrician who did the caesarean section, and there you go. In the meantime just figure it out." |
| **Towards parent-friendly vocational organizations**  Raising awareness of peripartum mental health issues  Who bears the responsibility to organize maternity leave (individual vs. company)  Acceptability of paternity / maternity leave  Adapting work environments to improve peripartum mental health  Parental leave: adaptable vs. mandatory  Extended maternity leave vs. early return | **Psychiatrist 2:** “**a specific part on basically work environments, and what company leaders should know about perinatal psychiatry and consequently what vigilance they should apply in their company, etc**.”  **Psychiatrist 3:** “Actually, that’s such an ordinary event that concerns absolutely everyone, and so absolutely all companies and even so, actually that’s not socially supported because that’s not acknowledged that, yes, well, actually almost disappear, and everyone is more fragile, but not at all. We’re not there yet.”  **Psychiatrist 2:** “And we see now that for instance depression in the general population […] is much more easily detected, well, that’s something which has really entered… in the general population, in GPs, in frontline providers, in companies too.”  **Psychologist 5 [female, 41 years old]:** “before that, often for some […] there was anyway still to bear the idea that it wouldn’t be easy. […] they cling a little to some ideals, transmitted by the family or transmitted in the business sector too”  **Psychiatrist 2:** “**That’s not institutionalized. While that’s you who bears this”**  **Psychiatrist 3:** “the day you become parent, you understand what being alone means**.** […] And that’s weird. Well, that’s weird to tell. But this is something that comes out a lot during consultations.”  **Psychiatrist 3:** “Well, I’ll talk a bit about myself, because being pregnant, I can testify of something, […] that’s things I hear during consultations. Although everyone is very nice and willing to adapt my schedule or to tell me “no problem, get some rest”, actually this rely only on me. So basically, it’s up to me to arrange everything, up to me to do all this process and so to constantly remind “by the way, I’m pregnant”, so basically, I’m not in depression, but I would say that basically, what that weakens is that finally, you feel not supported. To not be socially supported by the idea that it’s acknowledged, that it’s something that shouldn’t be discussed, I feel like saying that’s my problem and my concern. Actually, this doesn’t concern the society at all.”  **Psychiatrist 3:** “the small limit I personally see to this, telling it's a bit almost always the same thing, that’s as long as it’s left to the person’s appreciation and charge, actually, I find It difficult.”  **C&A psychiatrist 3:** “”We do not replace”. The maternity leave, you create a huge pressure on the hospital. So that means a cultural revolution of companies and in particular public hospital. It’s also how we consider pregnancies”  **Psychiatrist 3:** “That’s acknowledged that I will be absent and will not be replaced […] and I live more things I hear a lot during consultations, that is “that’s up to me to organize all, and actually, this is very complicated”.”  **Psychiatrist 3:** “Actually I was imagining an ideal where it would be taken care of by employers. Meaning basically: “I don’t want to see you, you’re on leave”. I’m telling myself that as long as it’s the responsibility of the employee, well partly, that remains actually a complicated measure, and that if finally people don’t have to be there and at worst the employer is sanctioned if her employee is there while she shouldn’t”  **Psychiatrist 3:** “We’re health providers at public hospitals so anyway we’re in […] something sacrificial, but there is always something like “that’s complicated to be absent, that’s complicated to decide to put yourself on priority” […] that weakens, that renders vulnerable. […] Because, as you said, I have the impression I’m the only one who bears this. But everyone is very nice (laughs), very kind, but that’s my problem, that’s the problem of no one else”.  **Psychologist 8:** “the way fathers are considered, well… can render problematic some things. There are some who tell themselves “ok I can take my paternity leave” and who take it, but I think there are still a lot of them who have pressures. […] So legally, there maybe some possibilities, practically, I think it’s not yet completely… well there remains work to do.”  **Mother 3:** “I was thinking “I arrive, I have two babies, I’m a woman”, I have to say I’m a young woman, so even if I tell them I have no baby they will think “well, she will want children at some point”. That’s things that are implicit during a job interview”  **Psychologist 8:** “Well, I think indeed there are some for whom it’s well accepted. I think in some companies, even if you’ve the right to take your paternity leave, are you for all that, well… you know, what is conveyed if you take it […] indeed that’s probably unequal depending on the companies.”  **Mother 3:** “above all I returned and asked, “well, why did I lost my position?”, and I was told “well, you realize, you’re mum of twins now, you can’t be as expert as you was before”. And at one point, I was even told, “you know, you have to choose your missions in life. Now, your mission is to be a mum”, while my only desire was to return to work. So I thought “actually they’re choosing for me””.  **Psychologist 8:** “I think that, well, that’s not always socially well accepted or valued [the paternity leave]. Well, that’s extremely recent”  **Psychiatrist 3:** “**being parent and working, that’s not incompatible at all, but actually that needs an adaptation. Necessarily. There is inevitably an adaptation just so that you manage to not get burnout or overloaded**”.  **Mother 4:** “**I** did not return to work because I quitted during my maternity leave and I found another job and when coming back to work, it did not went well because that was a job that implied too many responsibilities and trips. […] I think if I hadn’t been in a peripartum context, I would not have been that much impacted, you know. I still have the impression that I’m more vulnerable during the peripartum”  **C&A psychiatrist 3:** “My 1^st^ child was at the very beginning of paternity leave: 3 days. It was a weekend. You make more weekends and you would not necessarily stay with your wife and your baby. You went our seeing friends. That’s not like that anymore at all. So, really, culturally, that’s a way to be together.”  **Psychiatrist 2:** “And on pregnancy and postpartum, you have a little the opposite reflection for now, to tell yourself “well, I have to finish everything before leaving”, so eventually, there is an increasing workload, and when coming back from maternity leave, you get back everything that hasn’t been done, so instead of being spared, we rather have double work than less work. And so, that’s it, that there would be the opposite consideration in the workload incumbent on peripartum women, that would maybe be more reasonable”  **Psychiatrist 2:** “Ladies in the audience who were pregnant, you all experienced that it’s complicated to be pregnant while working, and to return from maternity leave when working, because it’s not well received”  **Psychiatrist 2:** “we’re not all in the same boat, because, for instance, work shifts, I mean at public hospital anyway, so nurses from I don’t remember which term, they work one hour less per working day, or 45mns, I don’t remember, meaning there is anyway a consideration that pregnancy itself is a tiring state, I think we all agree on that. But, well, there are some professions that take this into account and reduce working hours, there are some jobs that allow that, that allow to access remote working”  **Autistic mother 9 [40 years old]:** “I had also worries related to return to work, that was not that easy. Because when I return to work, I return at full-time and… as no one replaced me during my maternity leave, I was drowning in work and even had a burnout. So my return to work after the birth of my daughter was very bad. […] I preferred to change and to look for part-time jobs for that reason, to spend more time with my daughter and also to get some relief and manage to better cope with my life”  **Psychiatrist 2**: “that there would be an awareness of this high period of vulnerability, and potentially, why not, an adaptation of the working hours. That seems to me already quite good.”  **Psychiatrist 2:** “that’s very important because we don’t discuss this very much, there is quite few work on […] vocational distress and the peripartum”  **Women SMI 6 [36 years old]:** “well, the challenges… maybe to continue working. Because my job asks me to be really present and dynamic. Well, at the theater […] when I play, that’s emotionally, there is a lot of emotional discharge. […] So being pregnant at the same time, actually, I don’t know if that’s compatible. I don’t know I would be able to carry on my work.”  **Mother 4:** “I was not feeling that good to leave her at the daycare even if I was thinking “well, that’s normal, I work, parents today leave their children at the daycare”. […] you return to work and […] priorities actually change”  **Mother 4:** “well at work my manager told […] the team I wasn’t well, that they should leave me alone a little (laughs). And that was very nice. And so, well, I anyway managed to work but I think I was slower. Surely, that helped, I had kindness around me.”  **Father 1:** “I managed, even before my daughter’s birth, to take a day with her”  **Mother 3:** “I got assignments with meetings with the US at 7-8 pm CET, while I had two babies yelling day and night. I was asked to do trips while it was not planned in my contract. They imposed me days of vacation.”  **Psychiatrist 4:** “**Finally, the conclusion of all that, that’s it should almost be adaptable to each patient, even to each couple. For instance like in Scandinavian countries where they can share the parental leave, as they prefer, easily.** This kind of things. And that could be taken before, after, up to the person’s choice. And there are patients saying that in their company they can, if they wish to, it’s included, add, generally one month, up to one additional month. It’s in the contract with the employer and they are entitled to this. And I think it’s significant for them.”  **Psychiatrist 2:** “that’s saying “companies should get the measure of this issue and be force of propositions and that it should be their responsibility to make adjustments”, just like… well once again pregnancy is not a disorder, but it’s a period of vulnerability and so just like they will protect vulnerable persons, at some point it should anyway that there would be this institutionalization of this period, which is far from obvious. I think public hospitals are in such an abyss that it’s even more complicated to think about this at the public hospital, but once again, that doesn’t affect only public structures, […] but really all the structures concerned by this issue”  **Psychiatrist 3:** “the small limit I personally see to this, telling it's a bit almost always the same thing, that’s as long as it’s left to the person’s appreciation and charge, actually, I find It difficult. And I tell myself that even though it’s awesome that we have an extended paternal leave, and even in my relatives I heard I don’t know how many men who said, “I’m not taking it” or “I’ll take it later, that doesn't matter”. **Psychiatrist 5:** “There is a mandatory part!” **Psychiatrist 3:** “Yes, yes, but we all agree that that’s not… […]. Actually, that’s a proposition, and, in fact, let’s not forget that a proposition is something that should culturally be developed. Well, I don’t know, it’s a proposition, so once again it’s actually assumed only by one person”. **Psychiatrist 5:** “Once again, it’s just symbolic but the fact that there is a mandatory part, that changes things”.  **C&A psychiatrist 3:** “well, today at the hospital, the strategy when someone goes on maternity leave, well that’s “we lie low”.”  **Psychiatrist 2:** “when by misfortune women need to be absent longer than the 4 ridiculous months of the maternity leave, it incredibly messes things around. Because the hospital indeed doesn’t replace maternity leaves, relies on the fact that it would last 4 months and not one day more. And when you need to be absent longer, it deeply disrupts the care and all everyone want is that you come back on the double. Well, I think awareness should really be raised on this”  **Psychiatrist 2:** “even more at the hospital, this role of “everything will collapse if I leave”. You absolutely have to be there, you should not place the maternity leave on your colleagues who will replace, or not replace or are absolutely terrified at the idea of replacing…”  **Autistic mother 10:** “To have less… all that is mental load, organization, all that, that’s very hard. While working. Well, everything is doable but doing everything at the same time, that’s very hard for me. I think for everyone, but that’s true that given my condition, that’s complicated”.  **Midwife 23 [female, 39 years old]:** “**from one hand, maternity leave is very short and for some women it’s too long […] And paternal leave was also very short**”  **Mother 3:** “I had only one desire in my head, it was returning to work. Because I’ve always been a very career-oriented person. At this point, this was putting a spoke in my wheels, and I actually needed to feel useful.”  **Psychiatrist 2:** “We’re looking at early leave, well, at which moment women left during pregnancy. Knowing that’s more complicated data to take into account, because maybe if they left earlier, that’s because there was anyway a complicated pregnancy […] But still, there is the idea that maybe leaving earlier could be protecting too […] To testify myself, on my 1^st^ pregnancy I continued a bit beyond the maternity leave saying “I have 4 months so I prefer keeping it for after childbirth” and on my 2^nd^ pregnancy, I’ve been stopped 5 weeks earlier, or 4 weeks and half, and well, that’s actually really good. And if it was to be done again, well, obviously you have to leave earlier. This 4-months maternity leave is clearly not enough. And you have to come back too. […] But frankly, I can only recommend this to people.”  **C&A psychiatrist 3:** “Thinking about this, maternity leave duration before and after pregnancy… independently of identified disorders, because theoretically when you stop that means anyway that there is a problem. So, well, thinking about this because, we also know that […] there is anyway a lot of health providers who stop the patient because of a bit broadly, her wellness, without any severe identified disorder. And fortunately.”  **Mother 2:** “And I returned to work. And I think it saved me. […] Fortunately I returned to work (laughs). I returned to work at 80% […] My maternity was not very happy, but it was better when working. Because when working, I existed once again, in a job where I was skillful, where I was recognized. So it gave me a fix of confidence”  **Mother 3:** “At my 1^st^ return meeting, some people told me “aren’t you in parental leave?”, “well not”, “ah ok, we thought you would take a leave”, “but I never said that”, “yes, but well, when you have twins, you take a parental leave”, “well, no”. And actually, I felt like saying them “I wish you knew what I lived and here now you’re simply adding something.” |
