# Supplementary material for: Toward recovery-oriented perinatal healthcare: A participatory qualitative exploration of persons with lived experience and health providers’ views and experiences
Source: Eur Psychiatry. 2023 Oct 20;66(1):e86. doi: 10.1192/j.eurpsy.2023.2464 (PMC10964275; doi:10.1192/j.eurpsy.2023.2464)
Supplement: Dubreucq et al. supplementary material 4 — Dubreucq et al. supplementary material [file S0924933823024641sup004.doc]

Supplementary Table 4: Quotation supporting the theme "Challenging stigma" (**bold** figure in Table 2)

| **Challenging stigma** | |
| --- | --- |
| **Reducing stigma**  Public stigma (depression as a weakness of character or inability to provide adequate childcare) / Anticipated stigma  Reducing stigma associated to help-seeking  Reducing stigma related to mental health services and medication stigma  Reducing stigma associated to help-seeking (fear of CPS involvement and custody loss)  Reducing stigma related to assumptions on the fathering role  Lack of knowledge / minimization / banalization (from relatives, frontline providers and health providers (HPs))  Increasing awareness of PMHD in the last decade | **Mother 1 [33 years old]:** **“to appear as someone weak. And I never liked that, and for me that was a weakness […] that was not my temperament.”**  **Mother 3 [35 years old]:** “Then, I saw it written by my doctor on my sickness leave, it was written […], “psycho-depressive disorder”, something like that. And to see it written, I told myself, “ok, you’re part of that group”, while I have now experienced that, I tell myself there is no group, there is no stigma, but to see that, for me I was affiliated with that. […] And I told myself, “that’s it, all you didn’t want to, well, you’re inside”  **Mother 1:** “My husband, the same, that’s not someone who would have easily understood… For him, the depressed are people letting themselves go, you know? But that’s part… well that’s a lot like this in the countryside. I don’t know how to say, we’re in small villages, so that’s normal, that’s societal they all think that. And they’re hard workers. […] I had also this perspective, telling myself, “they all would see it badly”. My major fear was he that he would leave me. That…. Because for him, being with a depressed woman…. I find this word so strong by the way. “Postpartum depression” (waves her arms to make a circle). So, yes, that’s a depressive state, but I think that for everyone that’s the poor woman who doesn’t manage to care for their children. Above all, that’s what I feel when I hear people talking about this. […] Even myself. Now I understand it better, and by the way, I often try when some of my relatives give birth to look at… I pay attention to a lot of things. But often, people tell me, “well, no, don’t worry, I’ll not do as you did”  **Mother 1:** “to tell myself, “shit, that had to happen to me. I’m the 1^st^ one to have a baby”. Yes, more weakness, well. To tell myself, “I didn’t know how to cope”, well. And to tell myself I had to go to be helped and by my parents and my parents-in-law”  **Mother 1:** “There are those […] who are open to discussion and who understand, who hear and who are in empathy, saying, “well that must have been difficult”. And, so, there are those saying, “well, it would never have occurred to me” or “you could have rested more” or… more judgmental”  **Mother 1:** “I think depression will stay taboo for a very long time. Because it remains a weakness in people’s opinion. Even those who experienced it and got through, I think that’s one of the illnesses, […] which are the most difficult. […] But I think providers have a huge role in that.”  **Pediatrician 3 [female, 47 years old]:** “that would be good that there would be a flyer for the father and a flyer for the mother, because there are some families where you can’t say that you’re sad or that you’re not going well.”  **Midwife 19 [female, 60 years old]:** “**To not feel stigmatized when we want to orient them towards psychological support. That’s still classic to get the response that they’re not crazy**”  **Mother with serious mental illness (SMI) 8 [40 years old]:** “you must not hesitate to accept help. That’s complicated when you’re alone with your child and above all when you’re afraid. I know women are afraid of help because they’re afraid of being judged and all that, but I would say you must accept help.”  **Midwife 20 [female, 37 years old]:** “accepting help actually doesn’t mean not being able to be parent”  **Midwife 1 [female, 51 years old]** "What’s most complicated is for women aware to have a pathology, who are psychologically vulnerable, and who refuse any care, or who will be in a form of denial, with a rather defensive movement, for whom approaching the problem of her psychological fragility is complicated."  **Psychiatrist 3 [female, 32 years old]:** “that’s not that once they come to us that’s done, but actually, the thing is to manage to come to us. And in fact coming to us is difficult. […] In fact, the idea is to coming to the psychiatrist and in that story that, well, you’re pregnant, that normally that was the best moment of your life, that everything is going well… that socially there is no problem. And actually that’s totally wrong!”  **Midwife 24 [female, 35 years old]:** “you would need also to reassure on the side, taking an appointment for consulting a psychologist or a psychiatrist. […] I think there is a big apprehension on that side and that you have to find how to explain this, how to reassure.”  **Mother 1:** “So they made me see the psychiatrist… I didn’t like that, I’m not found of psychiatrists. Well, basically, I don’t like much talking […] it was a bit hard to confide myself”  **Midwife 25 [female, 60 years old]**: "there are mums who spontaneously and also some who are a bit reluctant [...]. Mums who are reluctant and that we’ll see late during pregnancy.”(p3)  **Father 1 [34 years old]:** “And even if that was not going well on my side, I still wished that… it was worth it.”  **Mother 4 [39 years old]:** “That was the 1^st^ time that I consulted in my life. […] but you see, that’s something in my family… my mother often did an analysis or stuff like that, so that wasn’t something taboo at all.”  **Mother 1:** “**people associate a lot depression with pills. […] So, well, reducing stigma, yes. […] People think a lot about psychiatry. And this word, “depression”, that means hospital, that means hospitalization, and I think that’s scary**.”  **Midwife 17 [female, 36 years old]:** “that’s really, for my patients, prejudices. That’s, the psychiatrist will put me on medication, so I’m already a grade above, while the psychologist we’re more into discussion. Anyway, the psychologist can’t put me any treatment**.**”  **Mother 1:** “I felt even more ashamed to tell myself I took an antidepressant.”  **Midwife 16:** “Some mums, as soon of you mention the psychologist, you immediately feel that […] they dig their heels in.”  **Mother 1:** “I had a bad experience of taking antidepressants”  **Mother 1:** “That was the fact to be helped by something chemical. I told myself I could pretty much find my way out by myself […] taking this pill made me feel even sicker. […] That was really the chemical side, to tell myself I had the impression to not sleep naturally, to not think naturally and I was a bit sedated. […] I found it not easy to disclose taking pills…”  **Mother 1:** “To say that’s not a treatment but a help. To wrap up all that so that it becomes pretty, and… not a punishment. […] That, when needed, you can take some […] that that’s only a help, a transition to get better.”  **Midwife 27 [female, 34 years old]:** “Another of my limitations too, when I think one of my patients needs psychological or psychiatric support, they’re very, very often afraid of medications.”  **Mother 3:** “I went to see my general practitioner [GP] […]. She prescribed me antidepressants I didn’t want to take because one of my very close friend is under antidepressants for 7 years now, and I saw to which extent it was difficult for her to stop it, I didn’t feel like to be in that situation, and I was persuaded I would manage to get trough on my own. So I did not take it, and my doctor told me […], “listen, if you don’t take it, go at least take them at the pharmacy and keep it at home because if you’re controlled by the Sécu [social security system], that’s possible they ask you the blisters”. So I was faking to take it, I was throwing it everyday to trash to show them “look, the blister is started”.  **Mother 4:** “that was more the antidepressant, where I was a bit, well, reluctant. But once I took it and I saw the good it did for me, I must say I was very happy. Because, well, you sleep better, you think less, the brain calms down and it allows taking thinks a little more lightly.”  **Pediatrician 1:** “there is a great worry, I think, from families, if they’re not “well-behaved”, firstly to be viewed badly, and secondly to not be good parents, and for families where there may be preexisting difficulties, there are some kind of red flags: above all, to make no waves, above all, not be viewed badly because it could lead to a report of the child.”  **Midwife 19:** “the fear, also, of interventions […] related to maternal and child protection [PMI] […] related to social services”  **Midwife 25:** “We also have some mums who had these experiences, sometimes a history of foster care themselves, some difficulties in their family, who already saw psychologists during childhood and are not willing to see some again. So we try to monitor, to support and to screen, […] to bring them to consulting”  **GP1 [female, 58 years old]:** “That double hat of the PMI [maternal and child protection]: to be there both to prevent, support, and in the same time being vigilant concerning protection […], there is that fear. The fear that it goes until report.”  **Mother SMI 7 [31 years old]**: “I was also supported by a midwife from the PMI. She went all weeks. […] Because my midwife told me, “you’re entitled to it as much as others”. Because for me that was… I had the image of persons with difficulties. And she told me that no. So I had before and after that person who came. That was very important and very helpful.”  **Midwife 25:** “that’s maybe not always easy to detect mothers who want to show that everything is ok for their baby and who maybe don’t communicate easily on their emotions.”  **Mother 4:** “And to break silence, that’s clear. Well, because I think some mums don’t disclose it because they fear to have their children placed, aren’t they? That still exists.”  **C&A psychiatrist 2 [male, 28 years old]:** “because they have the idea they have to be the strong man, that if they show they’re collapsing to their wife, they won’t support them.”  **Psychologist 8:** “regarding fathers, even if things have much evolved, there is still a long way to go. We recently had a father who was hospitalized at full time. That was actually a first. […] her partner died one month after childbirth. […] there was all an orientation worked with a maternal center that was ready to receive that father and his baby. The answer of the Department on the funding was, “that father has just to return to work. He has better confide his baby, to place him and to return to work. That’s not his place to be with his baby.” […] That was really surprising. […] Finally they were hospitalized in our service, and he actually told me to which point, well, first, for a man to arrive in a service where almost everyone was a woman, not simple! And he told about the shame he was feeling as a man to be in that process to get hospitalized, to seek help, and yet he told, “I have to admit I really need that help”.  **Psychologist 1 [female, 49 years old]:** “all the men I see that’s mainly on extremely traumatic births […] Also medically assisted human reproduction”  **Psychologist 2 [female, 40 years old]:** “that’s through the child we manage to get contact with fathers and to have their trust too, and then their confidences.”  **Pediatrician 1:** **“With all the dysfunctions you can imagine, phoning the firemen, “baby is crying”, “but, well, Mrs., that’s normal, children cry”, and all that kind of things.”**  **Midwife 14 [female, 45 years old]**: “that’s banalized a lot by relatives. […] A mum who just gave birth, surely she’s tired […] surely, she doesn't sleep as well as she used to. […] she doesn’t feel free to tell herself she’s not going well, because actually, around her, everyone send her the picture, “well, that’s normal, you’re tired, but your baby is going well”.”  **Midwife 17:** “fathers put all on baby blues. Meaning, “my wife is tired, that’s baby blues” […] without understanding that at some point, baby blues, that’s not that anymore. So I often tell them, “that’s 7 days. If after 7 days, your wife is not going better, you have to start asking yourself questions”. But, well, not all fathers see that too. And they’re also exhausted. […] When the baby arrives, they’re both tired and he’s returning to work and doesn't necessarily see it too.”  **Midwife 14:** “So, they have the impression that’s not serious. Mums are tired, that they’re coming home, that they’re going better. […] because that’s the reduction in hormones, because they’re tired, because that’s only crying, but, well not that much.”  **Psychologist 3 [female, 60 years old]:** “she went to a psychiatrist […], “it’s not going well, with my baby. I’m not feeling good”. But I think that given this psychiatrist was not trained to this issue, told her, “well, everything is going well”. So her request was rejected. […] Because she went to the right place and was not listened.”  **Psychologist 8:** “in populations a little more all comers, better socially inserted and all that, I find there is a real concern, meaning that even in terms of prevention. I think about a patient who was hospitalized to the mother baby unit and who explained me that in fact her baby had a stroke on his first day of life. Following that, parents were anyway relatively weakened, and apparently they weren’t told at all that if there were difficulties, they could maybe contact this or that service. […] and finally these parents went out their way to call out providers around them and each time resulting in something like a banalization of what was happening, telling them, “but that’s normal”, and she finally found herself the hospital, contacted us and she was hospitalized. She had a relatively severe, well, postpartum depression.”  **Obstetrician 3 [female, 34 years old]**: “there is an ignorance that relatives have of this disorder. […] they lack knowledge about postpartum depression”  **Pediatrician 4 [female, 48 years old]:** “the midwife in charge of breastfeeding here told me nothing. She met me, she told, “when I saw you coming home, I told myself you’ll never manage it”. [shrugs her shoulders]. But she told me nothing, she told me all the advices, “Well, safe return to home”. […] She told me a while after, “I wouldn’t have given much of your breastfeeding”.”  **Mother 4:** “I feel no shame on the topic and that’s why I try a bit… well, if I accepted to do this with you today, that’s because I wish it could improve for women. Even if **I observe that it has already improved over 7 years. Anyway, it has evolved. And I see it, a lot of things happen. […] The speech is freed, even**…”  **Psychiatrist 2 [female, 34 years old]:** "I have the impression that today, whether my relatives, the people I meet who are not from the field, etc., when we talk about postpartum depression, they know better that we are talking about a disorder, that it's frequent, that it exists, etc. Finally, I have the impression that it is indeed identified, in educated environments of course, but that it is still better identified."  **C&A psychiatrist 3 [male, 49 years old]:** “I think health providers will change too because the general public will talk about that. […] Depression is a theme that has been popularized for 2 years. […] Women come more easily. […] Women come for that reason and talk very spontaneously about […] suicidal ideations. Or women we screened and finally who are not cared for and call back telling “I know I have your contact details, I’m not going well at all, otherwise I’ll throw myself through the window, I have to come seeing you”. […] I have the impression, now that there is a movement. […] I don’t feel it was so spontaneous five or ten years ago, anyway, so supported by service users”  **Psychiatrist 2:** “I think that today, there is something much more acceptable and much more known on that, and I think there is an important work that is currently done on perinatal psychiatry in particular, and peripartum depression that’s let’s say, the emblematic thing of perinatal psychiatry.”  **Psychiatrist 2**: “Well, there is now a consideration of mental disorders existing in the general population”.  **Psychiatrist 2**: “We feel that people understand what we’re meaning, that’s clear, people hear that baby blues is not the same thing than depression, whereas […] when I defended my thesis on depression, my relatives used to tell me “ah ok, you’re working on baby blues”.”  **Psychiatrist 2:** “and you see now that for instance depression in the general population, that’s a topic that is much less taboo”  **Psychiatrist 2:** “I think things will progress anyway, […] you’re all concerned by heaps of research topics, clinical, authorities, whatever you want, and we struggle for showing the importance of that thing, both at the national level and at the international level. I have anyway hope that frontline providers would be more and more alerted and trained to that issue and that we wouldn’t have to train them in the field anymore, and the general public too.”  **Mother 1:** “I think you’re in such a state of moral, physical and mental weakness that in fact, you have the impression that everyone is judging you, that no one understands you and actually not at all. […] I even was surprised. […] That’s full of kindness” |
| **Reducing feelings of shame and guilt** | **Mother 1:** “**Well, completely [the feeling of shame]! […] What’s funny, however, when speaking again about this, well, I realize that many people hadn’t noticed. […] I believed it could be seen through me and in fact not at all!**”  **Psychologist 5 [female, 41 years old]:** “Mothers are feeling very guilty […] that’s very locking up […] the silence […]. They stay in a form of loneliness because they’re feeling guilt or there is a question of a little shame.”  **Psychologist 6 [female, 39 years old]:** “the issue of guilt of not feeling well. The arrival of a baby is indeed supposed to be a happy event, and then, well, no, finally, that can make feel sad, aggressive, and that’s true I think that’s very complicated to tell”  **Midwife 18 [female, 64 years old]:** “they don’t recognize it. There is the family factor […] shame about disclosing it […]. There is a lot of shame, how the family would view it, to feel diminished, to cry in family, telling themselves, “you feel incapable, you’re a bad mother […] while others manage it”.  **Mother 4:** “Well, I don’t know what my parents-in-law thought about my hospitalization […] That was my things, my choices, my decisions, anyway my partner fully supported me, that’s even him who told them. So I did not care, yes and no, I did not much have to tell them myself, saying, “I’m going to be hospitalized”.”  **Midwife 17:** “to tell herself, “why don’t I manage it while others […] I’ll master it, I’ll deal with it and I don’t need help”.  **Nurse 1 [female, 46 years old]:** “That’s true a lot of mums tell us they don’t dare to speak up because they feel completely judged and that they’re ashamed”  **Childcare Nurse 1 [female, 28 years old]:** “That’s difficult for them, well, to accept their condition, let’s say, uneasiness. Because that’s not necessarily a depressive state as it is. […] I work in an area that is quite precarious. So that’s mums who can be very isolated, very lonely, victims of domestic violence…[…] And the fact they don’t manage to be the mum they wished to be, that’s complicated to accept, to hear and so to accept support”  **Psychologist 1**: “Many patients talk about shame.”  **Mother 3:** “Because in fact I felt incapable. And I kept telling my husband, “I’m an incapable”.”  **Midwife 25:** “Because there is so much guilt they actually feel bad and that’s the baby that matters more and they refrain from talking about this”  **Psychologist 3 [female, 60 years old]:** “as soon as these mums, these dads, take care of their worries with someone else, well, maybe the baby has less to care for it ”  **Psychologist 3:** “That’s extremely important, they are understood. […] To get rid a little of the feeling of guilt”  **Psychologist 3:** “the question of guilt, shame […] when things are not going well with the baby because the mother is depressed or the father is depressed […] that’s terrible. […] the fact that they have ambivalent psychic movements towards the baby, even movements of irritation, aggressiveness or hatred, that’s absolutely terrible.”  **Pediatrician 1:** “that’s indeed difficult to confide in our reanimation field”  **Pediatrician 3: “**sometimes there are mums who can tell they’re sad, not happy with their life conditions and all that, who can name it and sometimes nothing comes out all this and honestly…”  **Mother SMI 7:** “So during pregnancy, I had a great fear of having again a depression, to be really unwell, and that the baby felt… I was too scared that the baby would feel all that was negative and after that of the impact afterwards.” |
| **High stigma for mothers who work as health providers or social workers** | **Mother 1:** “that’s very stigmatized, depression, whatever it is postpartum or not, I find anyway. And even still in our jobs, isn’t it? **I who am a nurse, I could see with my colleagues, if we care for depressive patients, there many who don’t understand it. And who tell, “no, but did you see, she doesn’t move during all day”, or, “she doesn’t work, she could motivate herself”. I who experienced it, now, well in fact that’s something hard. There is something stronger than not going out from your bed**.”  **Mother 2 [36 years old]:** **“Yes, I think, of shame and then the fear of being judged by… what that could produce. I’m a social worker, do you realize what that can produce?”**  **Pediatrician 2 [male, 74 years old]:** “that, that’s also the problem of medical professions […] and under the pretext that you’re a health provider, you know. So they let you go saying, “you know what to do”. […] Whereas you’re a mum first.” |
| **Experienced and anticipated stigma in mothers with SMI or autistic mothers** | **Woman SMI 9 [31 years old]:** “**Well, I fear being labeled mother who is potentially dangerous for her child. I fear to be told, “she is potentially dangerous”.** Not dangerous, but potentially that there are some vulnerabilities and that potentially there could be risks of… well… […] I’d like they leave me that freedom, being able to care for my child”  **Autistic mother 8 [37 years old]:** “**it will depend on the practitioner. If the fact of knowing I’m autistic, it renders less… they take it less seriously when I tell them there is that thing that isn’t ok, or that I tell symptoms and that’s not taken into account.**”  **Mother SMI 5 [30 years old]:** “Maternities, we’re only numbers, and that’s all the more complicated when you have a disorder, because when you get a right mouthful by health providers, so well, you go downer than people, let’s say ordinary, who don’t have any disorder. […] during the postpartum, the follow-up by nurses, who grumble, “but we don’t have the time to care for you, get by on your own a little”. Well, there is no support, no listening, I told several providers about the withdrawal syndrome, they all looked at me with bulging eyes. I had difficulties to have my usual medication there, so… that’s really something that is still unknown. That gives a little the impression that people with psychiatric disabilities, well… that they must not have children! That’s a bit hard what I’m saying, but that’s the feeling I had there, well… that’s, “yeah, don’t get under our skin, you’ve got a disorder, so you shouldn’t have had a baby”.”  **Autistic woman 6 [36 years old]:** “In addition to a free space where you could not be afraid of really telling what is ok, what is not ok, without feeling judged. And for the couple too, as long as you don’t do this alone. Because that could not be easy for the partner too, depending on how that could go, so that the person understand. And take this into account.”  **Woman SMI 3 [28 years old]:** “by helping me go beyond all the prejudices they throw back in my face everyday. So that I could, despite my illness, have a normal life. Which is currently not the case.”  **Autistic mother 4 [35 years old]**: "for the moment I still find it difficult to talk about the diagnosis (...) And when I go to the doctor, that depends, there are times when I say it, and times when... there is always this fear of judgment, that they will raise their eyes to the sky, that they..." |
| **Stigma related to lack of knowledge in health providers**  Lack of knowledge about peripartum mental health disorders (PMHD) / stereotypical generalizations about parents with PMHD  Perceived dangerousness for others when disclosing suicide ideations or SMI  Personal / family history of peripartum mental health disorders | **Midwife 17**: “the point indeed is that we could take them in charge. After, once again, I see them quite early during the pregnancy, so I don’t have the time to see the well-installed depression with the risks… **For** **me, suicidal risk is either immediately, but the big decompensation, meaning postpartum psychosis, the big thing, either it takes more time, a few months, a few years, and as a private practice midwife, I’ll have tendency to lose them on the follow-up level**”  **Obstetrician 1 [female, 62 years old]:** “depression, that’s the postpartum**”**  **Mother 2**: **"**I think that theme isn't easy to catch for providers, depending on their sensitivity, what they experienced themselves, on how far away their core target is... well, a general practitioner deals with many issues and maybe postpartum depression isn't one in which he is most trained and most comfortable" (p18)  **Mother 1:** “I think depression will stay taboo for a very long time. Because it remains a weakness in people’s opinion. Even those who experienced it and got through, I think that’s one of the illnesses, […] which are the most difficult. […] But I think providers have a huge role in that.”  **Obstetrician 2 [female, 32 years old]:** “I found it simpler as anyway it was mothers with risk factors, where there was previous pregnancies that were very complicated or a specific disorder during pregnancy that makes easier to figure these women out. I haven’t got any women, like the postpartum depression which can really be the woman who hasn’t any risk factor and decompensate for you don’t know really why. During the antenatal period, I didn’t have any.”  **Pediatrician 3:** “there are more and more fathers who are in the race, they’re much more present than before. Sometimes there is a father who isn’t well and what is complicated that’s when the father is anxious. […] we go in things that are difficult to contain. Or anyway, I have no ease for that.”  **Psychiatrist 1 [female, 43 years old]:** “I had a father who had symptoms, it looked like a feminine postpartum depression. He even had intrusive thoughts of harming the baby. But otherwise, you find other symptoms in postpartum depression in fathers.”  **Psychologist 8 [female, 30 years old]:** “in populations a little more all comers, better socially inserted and all that, I find there is a real concern, meaning that even in terms of prevention. I think about a patient who was hospitalized to the mother baby unit and who explained me that in fact her baby had a stroke on his first day of life. Following that, parents were anyway relatively weakened, and apparently they weren’t told at all that if there were difficulties, they could maybe contact this or that service. […] and finally these parents went out their way to call out providers around them and each time resulting in something like a banalization of what was happening, telling them, “but that’s normal”, and she finally found herself the hospital, contacted us and she was hospitalized. She had a relatively severe, well, postpartum depression.”  **Mother 3:** “I went to see my GP […]. She prescribed me antidepressants I didn’t want to take because one of my very close friend is under antidepressants for 7 years now, and I saw to which extent it was difficult for her to stop it, I didn’t feel like to be in that situation, and I was persuaded I would manage to get trough on my own. So I did not take it, and my doctor told me […], “listen, if you don’t take it, go at least take them at the pharmacy and keep it at home because if you’re controlled by the Sécu [social security system], that’s possible they ask you the blisters”. So I was faking to take it, I was throwing it everyday to trash to show them “look, the blister is started”.  **Woman SMI 2 [22 years old]:** "I had doctors who wanted to fit me into boxes (...) Well, being able to individualize, and not to stigmatize, and not to generalize."  **C&A psychiatrist 2**: “we are relatively poorly trained to detect these postpartum disorders in fathers […] because that’s not the same signs than postpartum depression in mothers.”  **Psychologist 3: “I don’t know if you can say postpartum depression in a psychotic patient who has a baby.”**  **Psychologist 3:** “the level of psychopathology. And yes, if you deal with a mum who is crazy, who can have a depression but who is a severe borderline, huh. The risk of acting out is a little more severe than if you have a normally neurotic mum, who has a depression and suffers much.”  **Mother 1: “**Even I as a nurse, I don’t know if that’s because I experienced a postpartum depression that I pay more attention, because besides, we see a lot of pregnant women.”  **Mother 2**: "I think that theme isn't easy to catch for providers, depending on their sensitivity, what they experienced themselves, on how far away their core target is... well, a general practitioner deals with many issues and maybe postpartum depression isn't one in which he is most trained and most comfortable" |
| **Providing clear, accessible and non-stigmatizing information about peripartum mental health (PMH) and wellbeing to all parents beyond universal routine screening**  Raising awareness on PMHD  Clear, accessible non-stigmatizing information on mental health and well-being during the peripartum  During antenatal period (e.g. early perinatal interviews, childbirth classes)  During the early postpartum period (e.g. postpartum advices, perineal rehabilitation)  Raising awareness on PMHD in fathers  Targeted vs. universal screening  Support for reducing stigma and opening discussions about PMH  Use of m-health tools to improve detection | **C&A psychiatrist 3**: "**general public information too**."  **Psychologist 6**: “relatives, actually, the father or a friend […] that we could raise awareness the relatives.”  **Midwife 14**: “that it would be part of the landscape, that everyone read these flyers, the relatives… That maybe we’re pointing out something that is frequent.”  **Midwife 14**: “that it would be part of the landscape […] Known and recognized**”**  **Father 1:** “I wasn’t surprised because as I said at the beginning, yes, if someone would have told me it existed, of course, I wouldn’t have denied it. That’s just that maybe I didn’t think it would happen to me. I would have been less surprised if it had been my partner, whereas she’s stronger than me, you see? […] But I wouldn’t have been surprised because there is the mental and the physical too. Well, I mean, childbirth is really a huge thing for the woman. So I wasn’t surprised to find few testimonies but really… we know that that exists”  **Mother 4:** “I feel no shame on the topic and that’s why I try a bit… well, if I accepted to do this with you today, that’s because I wish it could improve for women. Even if I observe that it has already improved over 7 years. Anyway, it has evolved. And I see it, a lot of things happen. […] The speech is freed, even… […] I was very well supported. Not judged.”  **Autistic woman 5 [33 years old]:** “I think that in maternity, there are some posters saying that if you’ve psychiatric problems, you should tell it to avoid postpartum blues”  **Psychiatrist 3:** “that’s very much a story of awareness of the general population”  **GP1:** “**maybe testimonies related to feelings, saying: 'I am afraid of hurting my baby'? Well, some questions or ideas that you may have when you’re not well. "I'm tired of my baby", "I don't like him", "I'm afraid not to like him", "I feel sad all the time, I just cry". [...] the impression that you’re not alone, because in these moments you feel [...] completely out of place. [...] dads too**."  **Psychiatrist 2: “**that it would be part of the information, just like we will inform women on Guthrie or breastfeeding if they wish to lactate, well, that it would be part of basic information and even very early, at the early prenatal interview, that there would be an information on peripartum depression that would be delivered, in itself, it would really be a very important leverage.”  **Midwife 23 [female, 39 years old]:** "something to reassure mums a bit [...] they set themselves a lot of objectives. [...] get back to basics in fact. A baby needs security and to be cuddled, he doesn’t need an exhausted mum because she did all the ironing. [...] A baby who cries, that’s anyway more or less normal"  **Obstetrician 2 [female, 32 years old]:** “something simple, or flyers… like the posters we have in the consultation rooms”  **Psychologist 3:** “how could we do to make these young pregnant women know that that’s not the dreamed life, that pregnancy is not a bed of roses, that envisioning a baby in a family, that’s not all rose and that’s normal, and that maybe they can discuss this with providers”  **Mother 4:** "Do we put testimonials? I'm not sure that's a good idea. I'm not sure that's a good idea. Saying that if you feel sad, isolated... but already starting… on the leaflets they tell you, "if you feel sad, isolated, that you don't know how to deal with your baby, or that you don't feel anything for your baby, well it happens, and there are solutions". Speaking of possible solutions."  **Mother1:** "That could also be good to give little booklets. That’s always good to have something you can take back home. And why not tell people that, in fact, they can talk to anyone. [...] put that they should talk about it. That it can be your pharmacist, your osteopath, your midwife, your obstetrician, your GP... also your parents, your friends..."  **Mother 4:** “put articles saying that maternity is not something all rose. We begin to know that, but the truth on that. On the reality of motherhood, not what is written in the magazines.”  **Midwife 26 [female, 52 years old]**: “I’m not ashamed to say, “perfect parents, that doesn’t exist, let go the pressure […] and that’s how you’ll learn your parenting role”.”  **Psychologist 3: "**What are the movements they can go through? The psychic, affective, emotional movements they can go through? The pregnancy and the aftermath of the pregnancy? What are the movements of sadness? Depression? Major depression? Depressive movements? I don't know if that's necessary to go that far, but maybe something that could be a support that could echo the person and already tell them that it exists [...]. She can recognize herself in it, that’s not completely crazy. The tool could allow to provide information that that would get away from idyllic things and that could accommodate the negative..."  **Midwife 25:** “I think they don’t recognize the signs. […] they can’t always perceived by themselves that they’re not well, that that’s not normal. And if you discuss that beforehand, I think it can echo and bring them to not staying alone. To discuss this with a provider of their choice.”  **Midwife 20:** “When pregnant, you have the right to be worried. Once you gave birth, you have just to be happy, you're not entitled to worry anymore.”  **GP 2**: "There are indeed a lot of information, on crying, on sleeping, how to do the ironing, the dishes, etc. when you have a very small baby who takes up all the space... And they could be more interested by these things at the start"  **Autistic mother 9 [40 years old]:** "Well, talking about baby blues too, that's true that it exists and you shouldn't feel guilty about it. That’s normal for women to be a little overwhelmed after childbirth."  **Mother1:** "And assuming to say "damn, I can't stand it anymore!". That was important to say that I had the right to say it and that, well damn if people hear me say it, that's okay. I have the right to not put up with my daughter's screams. And you have to accept that too, because that's not easy to tell yourself "she's only a few weeks old and I already can't stand her""  **Mother1:** “my midwife advised me that nicely: "you can put your baby in his bed. [...] You go to your garden and you will not hear his cries anymore, but for 5 minutes. A baby crying for 5 minute, that’s not serious. [...] That it came from a provider was reassuring”  **Obstetrician 2:** “simply some information [...] to say, "Well, sometimes you could feel overwhelmed, tired in postpartum”  **Mother1:** "I would have liked to type a little message, "I can't stand my daughter's screams anymore, could you help me? I tried everything, the osteopath, nothing calms her". I don’t know and maybe someone could have answered at that moment, “well, try to find a midwife's practice nearby, I know whatshisname or..." (p16)  **Mother 1:** “Where they could listen, saying, “well, no, you're no crazier than anyone else, not bearing your child because of the screams could happen, yes, that’s exhausting to hear a baby screaming all day long."  **Mother 1:** “which could reduce guilt too. That happens to many women. Something that would be accessible, not too many big terms, not too many numbers…”  **Mother 1:** "saying that we all have weaknesses, and that we have the right to turn to a provider if we have some doubts. [...] And that we have the right not to love our child right away [...] There is no perfect mother and there is no typical mum either, so accept yourself... [...] that’s important that it could appear in an application, saying: "there is no perfection, you are a whole person, and you’ll be a whole mother, different from the one next to you".  **GP 2: "**Mums are often full of questions when they’re discharged from the maternity, about the baby's rhythms, how a baby sleeps, how he eats, etc. That’s ways of access that they would be easily interested in because because they're confronted with it every day.  **Psychologist 3**: “Maybe also something a bit didactic [...] that finally, some of these movements are normal, ordinary, the questions of ambivalence, worries, fears, maybe that would be important to name it… [...] to integrate the dads too”  **Pediatrician 1:** "something with a visual allowing to make choices without necessarily needing to understand all the writing."  **Pediatrician 2:** "These leaflets could actually start with, "I just gave birth, I'm tired", or "I just gave birth, I'm not well" or whatever. Something very banal."  **Pediatrician 3:** "all these flyers made by Polygon, that are very, very good, that are very didactic, and finally that’s a way to approach the topics. It could be for example different topics: "my child cries, I can't comfort him", "I didn't sleep more than 4 hours last night, my mood is going down", finally all these events that can occur around the peripartum, where finally you lack sleep, there is a problem in life rhythms, the problem of the incomprehension of the child's functioning. Things that could a little bit address topics on children’ functioning, provide information and be a source of exchange with the doctor."  **Pediatrician 4:** "Could it be mini videos? Could it be a question? Or little drawings with smiles, I’m feeling okay / not okay. [...] But that could be either in the form of drawings, or even with several languages, why not? [...] we could imagine something quite short, with an image corresponding to a question, and a rather simple answer. [...] It could be in the form of small drawings, or funnier, small videos. [...] we could imagine a small drawing of the mother who doesn't wash herself, who stays all day pyjama, who doesn't wash herself, who doesn't eat anymore, who doesn’t eat anything, for example."  **Father1:** "that’s a good app by the way [the 1000 days mobile app]. That’s a good tool for young parents. Even with children around me, nieces, when that’s not your own, that’s not the same. And that’s a toolbox very practical for many things. Even today, [...] what can we do as an activity at one year old? Something that would both interest him and whet his curiosity. That’s a little toolbox."  **Psychiatrist 2**: "testimonies [...]. In particular a father whose wife committed suicide during the peripartum and who is going to give a testimony on camera, and I think that's really… I don’t dare saying that’s lucky considering the context, that would be inappropriate but still, that’s unique [...]. Well, anyway, testimonies, podcasts, well, all sorts of different sources of testimonies that could provide additional information with different perspectives and that would give access to pre-existing resources or external resources."  **Midwife 25**: "An app… to inform them well, so they would know who to contact. And de-dramatize that "I'm not crazy, I'm not going to see a shrink" side."  **Psychologist 1**: "Mothers with postpartum blues, they go on the internet. And as a result [...] they access to content related to postpartum depression because there are some websites. And that helps to clear the air a little, to well differentiate things"  **C&A psychiatrist 1 [female, 52 years old]:** "references to websites that could help mothers [...] but also fathers."  **Pediatrician 2:** "We have made leaflets, if you go on the Pédia Santé network, you can find topics that allow patients and parents to quickly identify, with regard to a crying child, what is worrying, what is not. With regard to a child with headaches, what is worrying and what is not. So on postnatal or perinatal depression, I think we could probably provide a common document, one that is written in common. That is, written with psychologists, psychiatrists, midwives, mothers, etc.... [...] That's presented in a very simple way, and not simplistic."  **Psychologist 2:** "I very much like a doctor in pediatrics, who is on Facebook. I find that he has a way of vulgarizing while being specialized in his field, but being accessible and funny. [...] that's a way of giving information that is hyper effective, efficient and pleasant to read in fact"  **Childcare nurse 3 [female, 37 years old]:** "indeed a platform for a "large public"."  **Psychologist 3:** "how could you make these young pregnant women know that it's not the dream life, that pregnancy is not all roses, that the idea of having a baby in a family is not all roses and that it's normal, and that maybe they could talk about it with providers. [...] Yes, maybe an application, I think that since these are tools that are hyper connected and consulted, that could be a tool that would be accessible.  **Psychiatrist 2:** "Another section that I think would be interesting, would be a "to go further" section, [...] where, depending on the topic, we could propose to the teacher to do a “to go further” section with whether vulgarization articles, scientific articles, testimonies and there we have Maman Blues who works on that with us.”  **Father 1:** "something that could work well, well that worked for me, [...] that’s indeed testimonies. Or people who could be listening [...] but anyway testimonies, maybe before, to realize that that does exist, that there are some people experiencing it, that we won’t all experience it in the same way, uh... I think that's never hidden, that fathers can be affected, but that would be nice if they were more represented. [...] That’s very often associated with the mum, and that would be nice hear some testimonies from fathers too. So testimonies for sure."  **Midwife 26:** "To put some testimonies of mums who went through that and tell a little bit about how they did it."  **Mother 1:** "testimonies from mums who experienced it. Because I find that it's still... that’s even better heard when that doesn’t come from providers [...]. I always appreciated fairly direct testimonies like that”  **Mother 1**: “that’s important to tell yourself “listen, there are such signs that will indicate that maybe you’re suffering from this depression”. So telling mothers, that, I don’t know “if you’re not sleeping that’s not normal”, “if caring for your child is too demanding, that’s not necessarily normal too”, “if you don’t feel like taking him in your arms, that’s not normal”… Well, even not talking about normality, because sometimes people… but you can say “it can alert you”.”  **Psychologist 6**: “**I** **think there is still awareness to conduct […] of these mums too, through childbirth classes. Anyway, midwives talk now more about this. […] to make some pedagogy or training, to be able to say to mums, well, to make difference between baby blues and postpartum depression. Well, that’s a speech I can regularly have in postpartum care. […] there is all that prevention work that is interesting**.”  **Psychologist 8:** “there is anyway more antenatal detection […] it would be interesting that there would be more systematic information to women, once they are hospitalized in a maternity, monitored, that they would be given a document saying, “well, this kind of things can happen, if that’s the case you can contact…” […] maybe a broader information at this level and to tell these women where they could go in case of necessity, or if they have these symptoms. I think it could be really important to broaden it, let’s say”  **Psychologist 3:** “the diversity finally of the signs that can tell about depression or not […] even again to make the difference between ordinary sadness, depressive movement and depression. […] And the manifestations such as phobias […] daring to tell they thought about putting the baby in the microwave”  **Midwife 26:** “we should talk also of all those who could be detected by the early prenatal interview, but to my mind that’s not developed enough. But after, we all do what we can […]. So I think there are small indicators that we miss, and that’s also women at risk. As I said earlier, those very at risk are clearly identified and the pathway is relatively clear and there are others, and that’s true that the early prenatal interview lacks painfully in those cases.”  **Midwife 13 [female, 40 years old]:** “I do couple childbirth classes. So, I discuss these topics and they both hear the same speech. And when it’s possible, he can attend to the early prenatal interview”.  **Midwife 19:** “There is the early prenatal interview, but also childbirth classes […] with fathers, the work that could be done around fathers, in fact for raising awareness among them […] we discussed systematically baby blues and what patients could feel when coming back home.”  **Mother 1:** “to discuss that beforehand. […] I followed all childbirth classes, and well, I did not hear about that. Anyway not enough so that stays in my ear. So, yes, discussing that beforehand, more at the midwives and obstetricians level, even discussing that every time. And even at the hospital, during all my stay, I haven’t the impression that someone discussed that so much with me.”  **Mother 1:** “I think it would be important that is should be done…, well, like a childbirth class. That should not be put in the middle of a class. I know me I wouldn’t have listened. Because you don't want to hear these things, you don’t want to hear what can go wrong. Well, so… it depend on persons, but I think it should really have been stipulated… even in mandatory. To say, “mums have to listen that”.  **Midwife 27:** “Actually, I think that’s also interesting to discuss that during childbirth classes […] that’s interesting to have discussed that in the antenatal period. Under the topic of postpartum. […] for me that’s an important information.”  **Midwife 16 [female, 50 years old]:** “Maybe discuss that more during the antenatal period? […] Because in that situation, sometimes it’s a bit aggressive to tell them like that about the psychologist.”  **Midwife 16 [female, 50 years old]:** “regarding discharge, we have a small maternity booklet where we discuss the possibility to consult a psychologist if coming back to home would happen to be difficult”  **Midwife 14**: “we manage more easily to introduce a psychological follow-up during pregnancies compared than during the postpartum, where they’re already not well, they’re worried […] I think that during pregnancy, we sometimes manage a little better to discuss this with them. […] The baby is not there yet.”  **Mother 4:** “you don’t have the instructions for becoming parent. Like during childbirth classes, well I couldn’t do it, but that prepare us to childbirth and a little to breastfeeding, but that could also be during childbirth classes. One session, during which you do prevention, awareness, on what is motherhood? That’s not all happiness, etc. And to discuss the great moments of happiness and the great moments of sadness. To avoid a little that emotional lift”  **Father 1:** "we bought a few books too before childbirth. But I would be lying if I told you that these were the chapters I read the most."  **Midwife 26:** “**during the postpartum care, […] I also tell them the signs.** Meaning that it could be insidious, “beware, if you’re experiencing low mood, if you cry a lot, if…”. And when the dad is there, I tell it to him, “beware, sometimes she can’t realize it”  **Midwife 12 [female, 54 years old]:** “I always try to tell them that if they don’t feel good in postpartum […], they shouldn’t hesitate to contact providers […] a psychologist, […] their GP or the private practice midwife […], or the PMI”  **Midwife 12 [female, 54 years old]:** “Or maybe discuss the signs of depression with these mums, […] telling them they can receive help in the case that occurs. Anyway, being able to discuss these signs beforehand during postpartum care, e.g. when we do the preparation to the return at home. […] we still don't have much the full network, but maybe discuss more this topic in the advices for returning at home in the maternity and connect with other providers. […] and free them from guilt.”  **Psychologist 6:** “the perineal reeducation […] that’s really the ideal moment to detect them and to orient them too”  **Psychologist 1:** “when I allow myself to discuss this with women even before something happens […] that’s when they tell me about postpartum blues. […] that’s a moment that could be about prevention.”  **Psychologist 2:** “there is a reduced feeling of guilt on all these motives: crying, parental exhaustion, a lot of gastro esophageal reflux disease. And pediatricians now systematically tell we call the psychologist”  **Mother 1:** “postpartum depression […] it isn’t supposed to be the things that happens immediately.”  **Nurse 1 [female, 46 years old]:** “informing mums, […] like that it’s exhausting […] maybe they could a little more turn to health providers, saying, “I heard about this and I believe I have the symptoms”.”  **Psychologist 6:** “that would make dads aware of their own risk of depression, but that could also make them aware to pay attention to the condition of their wives”  **Obstetrician 2**: “that mums would be aware that it exists, dads too […] that it would be known”  **Father 1:** “I often say, “I didn’t see it coming” and at the same time, when it happened, I immediately felt that there was something wrong. I didn’t keep it inside and actually I think that it helped. But I thought… Well, […] that’s just that maybe it wasn’t anchored in my software**”**  **Mother 1:** “you have to target women and future parents. Men too, because the partner is very important in all that care. And even a lot of information. I’m sure we could make boards that we can be hanged at doctors, at midwives’ office. Because, without realizing it, you read what is in front in your eyes in the waiting rooms”  **Psychologist 4 [female, 38 years old]:** “there is awareness towards fathers with the idea of childbirth classes for fathers”  **Midwife 13 [female, 40 years old]:** “he can have that postpartum depression too […] and that can initiate an instability at home.”  **Midwife 25:** “I would say that’s indeed often complicated to bring them to care [the fathers]. And to diagnose depression too.”  **Midwife 19:** "**Our psychologist intervenes twice a week on half days and sees a large number of mothers in a systematic way. Meaning that we don't tell the woman, "I find you a bit tired, would you like that to see the psychologist?”, the psychologist just goes see her. Well, not at all intrusively, but rather systematically** because she had a C-section, because her delivery was long, because that was difficult, because the expulsion was a bit traumatic, because the baby is in neonatal care. So with that knowledge, she goes to see the parents, because she doesn't necessarily see mums alone, and she introduces herself, and that’s a first contact. **And very, very often that leads to [...] real psychological interviews. While initially these mums wouldn’t necessarily saw her if there wasn't that systematic contact**"  **Midwife 23:** "on red flags, well, if you have clinical doubts you’ll ask it quite easily. Well, in my experience, I didn't systematically ask that question"  **Midwife 18:** “we keep the relationship but that’s very complicated and sometimes we lose them and we get them back later on, or we have news, because they decompensated or they’re very, very unwell. But later. And finally we let quite a lot of time fly during which we could have done something.”  **Psychologist 8:** “to have something broad because finally, there are some who aren’t detected in the antenatal or the postnatal and that for that matter can have symptoms, either that appear later on, or that indeed are… that’s discreet enough to avoid being noticed by providers.”  **Psychologist 2:** “**it removes the taboo and something that is both mental and physical care […] I find that the way they’re seen by midwives too, that’s… […] if indeed they’re at ease like when talk about contraception, if they discuss mental disorders the same way, that’s accessible and that’s not taboo. That doesn’t bring uneasiness to them. But I think that for that they have to be at ease**.”  **Psychiatrist 2: “**that is to say**, some realize the screening using EPDS [Edinburgh Postpartum Depression Scale] during postpartum care, but the midwife who has to administer the questionnaire, she’ll not drop the scale in the patient’s room and leave, she has to contextualize things a bit, so to evoke that the point is to detect depression, so, what’s depression, what is its frequency, what are its symptoms, etc.”**  **C&A psychiatrist 3:** “I really believe that it allows accessing to a population who wouldn’t have any demand otherwise. […] a woman who was screened 14 at the EPDS, that’s not terrible. Yet if she cheated, she answered 0 to question 10, because she told herself, “otherwise I risk staying at the maternity”. […] And then the baby is 3 weeks old […] she says, “I’m at the end of my rope, I can’t hold, I need help”. She has an history of depression that was already identified by a GP more than one year and half ago, but that’s through screening that something formulates itself and she makes an explicit demand of care”  **Psychiatrist 2:** “one of the biggest difficulties, that’s the absence of screening that comes with late diagnosis. So finally patients come to us with more severe symptoms that is more difficult to take care of and more difficult to treat and to improve, with potential consequences, whether it’s for them or for the baby if it’s during postpartum, or for the whole family, that are much more important. So, well, the delay before accessing care that is to be related to the absence of screening or to incomplete screening.”  **Midwife 14:** “postpartum visits, whey we see them at one month and half - two months, actually that’s that we feel they’re sinking. […] And that’s true that if we don’t ask the questions […], they will not necessarily tell us because, well, at home, they always send them back the idea that, well, that’s normal, “you have to take it upon yourself”.”  **Midwife 27:** "Well, now there's the Edinburgh scale? I give them the questionnaire from time to time and they complete it at home. I know that sometimes it helps me for discussing that.”  **GP3 [female, 52 years old]:** “I was GP at the beginning, and mothers tell me… first, I ask them, if no one asks them the question, they won’t tell. And if you don't go looking for them, they won’t tell. So I give them the choice. Do they feel like telling it or not? […] I haven’t that problem or that reluctance to talk with parents.”  **Mother 2:** “And maybe an interview guide or a questionnaire to be completed by the woman, well, a support of discussion for all providers who don't dare to open the discussion. […] our health system in France results in women meeting some providers. That’s not a system where a woman experiencing her postpartum depression, experience it alone, without any interaction. […] Which is pretty good news for me because that means there for these providers to grasp this question. Except that, at first glance, these occasions are actually not oriented towards these questions. You’ve the postpartum visit, in which you’re told about your episiotomy scar, but finally we could discuss something else, you’ve all the visits to the pediatrician, all the lactation consultations. There are many occasions for women to connect with providers.”  **Mother 2:** **“**I have the impression there would need a support, to facilitate discussions between providers and patients**.** And I think anyway that given patients are those who suffer in that situation, the patient can’t be an expert when he’s still… I strongly believe to… I think that change comes from patients once they’re able to take hold of their experience and to transcend it, and well. But, well, immediately, when you’re at the end of your rope, you need that the provider put some words, ask some questions, open some doors, that you take or not.”  **Midwife 24:** “ask that question even if sometimes you can sometimes feel a little uneasy.”  **Pediatrician 3:** "And then there could be items on emotions, on sadness, on relationship with the father... if only to allow discussing some topics we don’t cover and that could serve as a consultation tool that could be used in a transversal way by different providers who act during the peripartum. What’s difficult sometimes is daring to tackle the subjects, and sometimes when you have a tool that allows us to put your feet in and open spaces for discussion."  **Midwife 14:** “we all worked a lot on all what is violence, and you notice that when you don’t ask the questions, the topics don’t come out anyway. […] So, well, the questions, I think you really have to ask them”  **Mother 4:** “postpartum depression. You must communicate on that. Well, yes maybe by giving some kind of questionnaires”  **C&A psychiatrist 3:** “the interest of screening, that’s that too, to bring always, always, the mental inside the… it has a vocation to tell about their patients’ mental problems.”  **Mother 2:** "**you can take the EPDS on the 1000 days app, I think that’s a very good thing.** It's worth what it's worth, that’s not the perfect tool, but at least that allows the woman, without any commitment because that’s at home, anonymously on her phone, to already situate herself. And maybe open up a space in... to tell herself, "maybe what I'm experiencing is not normal." I read the questions carefully, I tried to think about what I would have answered at that point. But in fact the EPDS would have made “bip bip bip” [mimics flashes with his hands and laughs] And that’s sure that if I had had that tool in my hands at that time, I would have told myself, "Actually, that's not going well at all!". **I think that would have opened up some questions for me. It would have already posed something, it would have made what I was experiencing exist. I think that would have made sense**."  **Mother 1: “**maybe a depression scale. Or for a little exaggerated baby blues [laughs]. But yes, from zero, everything is ok, to 10, that could be, "I don't sleep very well at night, I have dark thoughts", or "I can't stand my child's cries". Or "I don't want to care for my child". And to tell yourself, "well, when you go beyond five, maybe you should tell your doctor, or someone you trust". [...] I always liked these little scales at least that allows a self-assessment. And to tell yourself [...], “Well, I’m to that point, already at 7. And also, “I want to leave home”.  **Childcare assistant 1 [female, 32 years old]:** "In a mobile application there should be a questionnaire for patients that they could complete in their state, how they feel, on their fatigue, their emotions, the crying"  **Psychiatrist 2:** "self-testing, self-assessment, and I think that’s something the 1000 Days mobile app does indeed offer, but, well, that there would be the EPDS but also that users could complete it or that health providers could have that tool available and easily accessible."  **Midwife 12:** "the woman would fills her questionnaire [...] she would receive a link according to her answers and she would be offered care or support, or simply to come and talk about that.”  **Father 1:** "[...] the association was via 1000 days. [...] I was contacted very quickly” |
| **Training frontline providers on PMHD (knowledge / attitudes)**  Knowledge of peripartum mental health disorders  Knowledge of treatment options  Knowledge of / attitudes towards screening tools  Knowledge of the referral pathways / available resources | **Childcare nurse 2 [female, 37 years old]:** “**training is very important, we still have a lot to learn and see**.”  **Psychologist 3:** “about providers’ awareness, I think to GPs too […] on what could be the signs of a postpartum depression”  **Mother 1: “**Even I as a nurse, I don’t know if that’s because I experienced a postpartum depression that I pay more attention, because besides, we see a lot of pregnant women.”  **Psychologist 6:** “raising awareness in providers on that clinical examination too, perinatal care, which is very particular”  **Pediatrician 4:** “Well, I don’t know if midwives are well trained on postpartum depression. Probably more than us.”  **Psychiatrist 3:** “that’s very much a story of awareness of the general population [Psycho 8 nods], and among other things, I’m telling myself that in fact health providers…”  **GP1:** “that’s also the need for training as well childcare nurses, midwives or doctors”  **Psychologist 2:** “They [midwives] are willing to know the symptoms and to know how to handle postpartum depressions”  **Psychiatrist 2:** “**when a patient is pregnant, people who are untrained will tend to say, “oh, let’s stop or at least reduce psychotropic drugs in late pregnancy, that will be better for the baby’s arrival”, whereas, in fact, data rather supports the opposite: there is a volume increase, doses should be increased at this period rather than reduced**.”  **Psychiatrist 2**: “Yes, and similarly in adult psychiatry. Adult psychiatry, anyway, there is a rejection of mothers’ peripartum care saying, “oh no, that’s a mess, I don't know how to do it, that’s complicated. There is a baby in the belly so psychotropic drugs I don’t know, the interactions, I don’t know, that’s very complicated”. I say that when I teach classes, “interactions, that’s first looking at what is happening.” […] There is also some very basic things that are within the reach of anyone. But as says Psychiatrist 5, when you’re not regularly confronted to the situation, you’ll not participate in training, rise your skills on that topic, because at the end that doesn’t correspond enough to your activity.”  **C&A psychiatrist 1**: “They often look at the CRAT [center of reference for teratogenic agents] to have information, but there are things that are missing very much, I think, to the training of GPs and maybe indeed to have access to psychiatrists and prescriptions, all their knowledge, I find it interesting too.”  **Pediatrician 2:** “**there is a maternal depression scale made by the French Association for Ambulatory Pediatrics […] that is well constructed, that I never use. But anyway, I know that scales exist**.”  **Pediatrician 3**: “I saw they put a scale too. I don’t know how to use it.”  **Midwife 8 [female, 35 years old]:** **"**I think that what is important is that we could all speak the same language, so effectively assessments grids... and that we could all base ourselves on validated grids"  **Pediatrician 4:** “Because the problems with too long scales, [sighs]… we don’t use it”  **Pediatrician 2:** “finally, screening algorithms aren’t used much.”  **Pediatrician 3:** “I find that when we pass scales, e.g. of depression, we put a diagnosis on the mother. And just that, that’s a charge. That means that the mother isn’t going well. But is the father unwell? Is the family supportive? Is the family dysfunctional? Couldn’t we put this too? […] but finally why the mother is unwell ? And I think that it makes them bear some guilt too. […] putting a diagnosis of depression only on the mother, I find that sometimes it burdens her even more”  **Psychiatrist 2**: "Either by lack of resources [...], “Your screening system is great, but we don’t have anything behind, so, in fact, we can see that some are suffering, but we don't know very well to whom to refer them, etc.", so there is also that difficulty, that finally they only come when they’re really in extremely severe cases and so that’s a bit late… too late."  **C&A psychiatrist 3:** “the interest of screening, that’s that too, to bring always, always, the mental inside the… it has a vocation to tell about their patients’ mental problems.”  **Midwife 18:** "there is much positive in screening these peripartum depressions, referring them either to networks, to psychologists"  **Mother 4:** “I felt very unwell, and I told her, “well, I feel like being hospitalized. I have to be taken in charge, I can’t cope anymore”. **[…] I went to my doctor at that moment who was desperate too, who didn’t know about mother-baby units, who tried to contact a local hospital. […] She managed to tell me, “I leave you a message. That’s not a psychologist that you need, that’s a psychiatrist, maybe your treatment should be adjusted”, well the poor was a bit panicking. She left a message to a psychiatrist who called me back the day after**.”  **C&A psychiatrist 3:** “Recently, a melancholic woman hospitalized in our own hospital, followed-up for her pregnancy in a hospital with which we work regularly, well, nobody told us before childbirth. Never. Everything was to be built. She lived 100 meters from us. They never thought to contact perinatal psychiatry. Why not? That’s repeated, that's regular”.  **GP2: “**That’s to have an answer to provide to families” |
| **Training frontline health providers (skills)**  How to discuss PMH issues and create a trusting relationship  How to start discussions about PMH (fear of being intrusive in particular for those without prior history / identified risk factors)    How to evaluate suicidal risk (discomfort for health providers; not in midwives thoughts; assessment only when explicitly told)  How to handle psychological distress  Deciding what and how to disclose to other health providers | **Childcare nurse 1:** “**That’s often complicated for us to make the mums adhere with the follow-up, or anyway to put in place some support […] even if putting in place TISF [technicians in social and family interventions], […] that’s sometimes difficult for them to hear because they already feel very guilty about the care they can provide to their baby**”  **C&A psychiatrist 1:** “trust is important and sometimes that is well brought by some providers who anyway know how to bring it. […] And that’s an experience too the issue of trust and kindness.”  **Midwife 18:** “how to bring the mum back to reality, to her difficulties? Because our care is mostly antenatal. During the postpartum, we see them once or not anymore, and that’s very, very, complicated to make them hear they experience difficulties, that we can support them, even refer them.”  **Midwife 1:** “I rarely interrupt a person who is talking. […] Then I systematically return on a form of reformulation of what was told.”  **GP 1**: “that’s time, that’s connection, that’s listening”  **Midwife 1:** “to receive a couple or an isolated mum, the 1^st^ thing, even if that can seems banal, is to create a trusting relationship. […] to allow them to be at ease.”  **GP1:** “simply by naming things, by not hiding things, by being rather in the dialogue with families and hospital services. […] the possibility to tell things to each other and to explain it to the mum, the dad, sometimes to family relatives so that they could be involved too, not feel put aside. And also to try to reassure to move forward. But yes, that needs a tight partnership and to respect each one’s place, that’s not always easy, and to know well what the ones and the others do and what are our skills, or ways of doing things too.”  **Father 1:** "once again maybe that what helped is to come across the right people. If I had came across to someone… because that could happen, right? On a midwife who would have say, “the 1^st^ weeks are always complicated, you know” and well, that’s it [shrugs his shoulders]. We didn’t do too much nor… [...] that was taken as it should be.”  **Nurse 1:** "explaining in simple words what postpartum depression is, that helps to build the relationship and that they could relate to themselves and also put down what is not going well."  **Psychologist 2:** “I was telling myself, on shame, probably that the way frontline midwives discuss this or intervene… they make it more accessible and, so, that there was something easier for patients to confide themselves.”  **Midwife 18**: “that’s mostly the issue of trust. […] We intervene at home, everything can go well during several meetings and from one day to the next, they don’t open the door anymore and we remain very worried.”  **GP 1: “That’s in the subtleness of not turning them on us, to avoid breaking the relationship too. And sometimes when we contact the psychiatry, that’s much more complicated.”**  **Midwife 17:** “**I have more difficulties in postpartum than in antenatal**. I must admit that in the antenatal they talk more. You’re more on worries. Anyway, I manage more to discuss it […] in the antenatal period and **in postpartum I really have this, “no, that’s not severe, that will pass”**.  **Midwife 17:** “everything goes well. And so, we start discussing with her, well, psychiatrist, etc. and so, well, she don’t come to her next appointment […] as soon as we tried to introduce the PMI […] that closed the doors”  **Midwife 25:** “We also have some mums who had these experiences, sometimes a history of foster care themselves, some difficulties in their family, who already saw psychologists during childhood and are not willing to see some again. So we try to monitor, to support and to screen, […] to bring them to consulting”.  **GP 1:** “After, that’s convincing mums to go”  **Midwife 12:** “For those without prior history, we still will ask ourselves when we’ve patients crying a lot, who are sad, who can’t take care of their baby, when we feel that’s complicated, or conversely who are very, very demanding. […] We see that’s complicated with their baby, who ring very often, who always need to have a provider alongside them, supporting them.”  **Midwife 26:** “I sometimes discuss it during postpartum care, when I see the vulnerability set in, to ask the mums if they’ve a prior history of depression, if they have some things in their history that can make them more vulnerable”  **GP 3:** “I have very small things to detect them, well, that’s not necessarily to look for… to be too intrusive. First, saying when they come back home, “do you manage to eat? To go to the bathroom? To shower?”. Small things of daily life, if they manage to organize themselves. If they’re overwhelmed in daily life organization, I think you should look for something else. […] If they manage to organize themselves, I’m quite relaxed, I tell myself, “they’re holding up”. If they can’t, if they don’t manage to eat or shower anymore, that alerts me.”  **Mother 2:** “And maybe an interview guide or a questionnaire to be completed by the woman, well, a support of discussion for all providers who don't dare to open the discussion. […] at first glance, these occasions are actually not oriented towards these questions. You’ve the postpartum visit, in which you’re told about your episiotomy scar, but finally we could discuss something else, you’ve all the visits to the pediatrician, all the lactation consultations. There are many occasions for women to connect with providers.”  **Midwife 24 [female, 35 years old]:** “**that’s not so much that we don’t want to ask it or that we’re afraid to ask that question [suicide ideations], well, for me, that’s my romantic side, a bit naïve, that’s no [laughs]. Unless,** as said earlier midwife 26, **the woman express it clearly and from that moment, we’ll put all in stage for the care**”  **Midwife 26:** “I don’t have it in mind at all. I don’t think to discuss it. Except of course if she evokes dark thoughts during postpartum care. […] Well, unless she discloses immediately dark thoughts, […] I wouldn’t have discussed that with her”  **Midwife 21 [female, 38 years old]:** "postpartum hemorrhage is a leading cause of mortality, we realize that… well, that’s with us within the service. Whereas suicide occurs finally weeks, months, or even in the first 1000 days, well, in the next 2 years, and when you are a hospital midwife, you will never be aware of it. And so that’s invisible for us. [...] but that we could have some kind of feedback on whether there were perinatal deaths in the first two years. I don't know how that could be possible, but to be aware"  **Psychologist 2**: “what helped me to evaluate suicidal risk, that’s my training to RUD [risk, emergency, dangerousness]”  **GP2 [female, 47 years old]:** "that’s my fear. To miss that [suicide risk] in fact. That danger in particular. By not being reactive enough."  **GP2: "**that sense of urgency, for me that’s that [suicide risk]. That’s when you think there's a danger..."  **Midwife 19:** “For that matter, I don't know how to do this concerning screening. I think that’s something very different. I'm not really convinced we could identify suicidal risk. I was confronted to 2-3 situations where the women said it, or things were expressed, so we took care of it."  **GP 2:** "Because when they end up saying they’re not going well [...] we really feel like we're in an emergency."  **Pediatrician 1 [female, 54 years old]**: "We had some mums who have attempted it [suicide] with medication or things like that. The ultra-catastrophic situation I told you about earlier was a shaken baby situation. So a mum who was signposted [...] in fact, she shook the baby almost immediately after maternity discharge.”  **Psychologist 5:** "as far as the risk of acting out is concerned, the psychiatrist is essential in this loop.  **Midwife 2 [female, 38 years old]:** "it took me more than an hour and a half to get her hospitalized, psychiatrists being unwilling to receive her right away, giving me an appointment for the next day. So I was very worried, worried about the baby, worried about everyone. I even had to call one of my colleagues for support [...] It took half an hour to put her in the car with my colleague, to send her back to the hospital when at the end they accepted to receive her. So really a very, very uncomfortable, very unpleasant situation. A lot of worries as private practitioner."  **Midwife 13:** “**to be confronted to the patient’s distress, I think that puts us in a difficult position**. […] Sometimes, that’s complex patients too, […] we would prefer to forget all about the consultation because we know that sends us back to a difficulty.”  **Midwife 21: “**they will say, “well, I already had a postpartum depression, that’s something that scares me”. And in fact, I feel currently resourceless to answer them well.”  **Midwife 5 [female, 58 years old]:** “because in private practice, we’re a little, well… [swallows] alone facing ourselves and the patient… and the couple. […] I find that we have many situations of depression”  **Pediatrician 3:** "we don't have any space to take another look on things. That’s true they give you an emotional load or a suffering load, and, well, sometimes you don't know how to deal with it. You come back with that at home, because you perceive something but you don’t have any space to take another look at it. So there should really be something that could exist, because that’s a network I think. And that has to be waved, thought and elaborated.”  **Midwife 15 [female, 47 years old]:** “that’s often mums who are not really willing to ask for help and this, we rapidly feel resourceless during postpartum care”  **Pediatrician 1:** “**There is that gap where you’re told, “we’d like care at discharge from maternity because this mum will need some support and not to be separated from her child”, and suddenly, something terrible happens while we did not understand anything. Which renders for instance the concerning information very difficult in some situations because you tell yourself, “what will I start?”**  **Obstetrician 1:** “beware of stigma. […] Well, when that comes to psychiatry, that’s very complicated to write and very complicated to transmit. And that we would once again reflect, on how we’ll transmit from one to another on the vulnerabilities of the woman. […] Because that’s concerning when you see the woman for the 1^st^ time, that it would be that that appear first. You have to write she’s vulnerable, that they should pay attention to her intimacy, you have to transmit things, but you have to transmit it [hesits], well, delicately. […] In a tool, you have to find how to transmit on psychiatric disorders. Similarly, you must not label them “psychotic”. […] you have to learn how to transmit. That’s really a common language we have to develop in peripartum care that points out vulnerabilities without stigmatizing them and without locking the patient inside. […] That’s pregnant patients, you shouldn’t stigmatize them, that’s really important”  **GP 1:** “that’s the issue of secret, of what we share, the information we have to share”  **Pediatrician 1:** “there is information that is transmitted and that is altered. The bigger the team, the faker the information. […] what do we share? […] with the psychologists, we try to agree on what we share or don’t share. […] We try to share some things that are obviously absolutely imperative […]. But there are some things we don’t share within the big team because that alters the picture of what people could do if the information was altered.” |
