# Supplementary material for: Toward recovery-oriented perinatal healthcare: A participatory qualitative exploration of persons with lived experience and health providers’ views and experiences
Source: Eur Psychiatry. 2023 Oct 20;66(1):e86. doi: 10.1192/j.eurpsy.2023.2464 (PMC10964275; doi:10.1192/j.eurpsy.2023.2464)
Supplement: Dubreucq et al. supplementary material 5 — Dubreucq et al. supplementary material [file S0924933823024641sup005.doc]

Supplementary Table 5: Quotation supporting the theme "Empowerment and personal recovery" (**bold** figure in Table 3)

| **Empowerment and personal recovery** | |
| --- | --- |
| **Personal recovery**  Personal growth  Hope  Identity extending beyond peripartum mental health disorders (PMHD)  Importance of social roles beyond the parenting role  Relief / sense of legitimacy after diagnosis  Overcome childbirth trauma  Connectedness with the baby  Connectedness with partner  Connectedness with providers  Being involved in peer support activities | **Mother 2 [36 years old]:** “**During all that time, I had much grown up. I often say that having lived all that, I feel I went deep into myself, but I came much higher than I would ever have if I hadn’t lived all that**.”  **Mother 3 [35 years old]**: “I often heard, “I reached the bottom and now I’m back”, and I was telling myself, “but why do they say that?”. In fact, that how it is. I looked deep into myself for who I really was and what I really wanted, and above all, what I did not want to endure anymore. And that’s that, that resulted in finding who I am. Actually, I think that before all that I only myself on the surface. I did not go deep into myself and into what I wish in my life. And that in fact, that built all I was missing.”  **Mother 2:** “I think it gave me a certain emotional, intellectual density. Well, at the end, that was certainly the most difficult ordeal of my life but in fact with perspective, I wouldn’t wish to have lived it otherwise because that really built the person I’m today.”  **Mother 4 [39 years old]:** “Sometimes we had very hard times like that, but then, how… well, that’s hard, but how we enjoy it even more, you know? I haven’t any regret. […] By the way, I didn’t told you, but when I was pregnant, when discussing it with my partner, I told him, “you know, even if I redo a postpartum depression, we keep it”. You never can tell, I was pregnant, well the beginning. Saying that maybe I already felt it. But saying that, that’s anyway worth redoing it.”  **Father 1 [34 years old]:** “the wave is the good word because finally I have the impression to drown. […] I didn’t see a way out, you know? […] And **that was in particular [EM, the president of Maman Blues] who told me,” don’t worry, it will take weeks, months but there is always an end”. And that’s small words that do good. […] even if that will not happen right now, you know there will be the end of the tunnel. Or small progresses. That was more with […] a psychiatrist. […] She outlined the small steps that you don’t notice. […] Even in the first weeks, there is still some positive moments**.”  **Father 1:** "something that could work well, well that worked for me, [...] that’s indeed testimonies. Or people who could be listening [...] but anyway testimonies, maybe before, to realize that that does exist, that there are some people experiencing it, that we won’t all experience it in the same way, uh... I think that's never hidden, that fathers can be affected, but that would be nice if they were more represented. [...] That’s very often associated with the mum, and that would be nice hear some testimonies from fathers too. So testimonies for sure."  **Midwife 26 [female, 52 years old]:** "To put some testimonies of mums who went through that and tell a little bit about how they did it." (p13)  **Mother 1 [33 years old]:** "testimonies from mums who experienced it. Because I find that it's still... that’s even better heard when that doesn’t come from providers [...]. I always appreciated fairly direct testimonies like that”  **Mother 3:** **“Actually, the fact of doing a series of video interviews gave me more strength each time because I was telling myself, “maybe that will not be this job, but that’s it I’m getting back on top, I’m coming back in the game” [laughs], that was that in my head and, “I’ll manage to do it and you’ll understand what I’m capable of, I’m not just the mums of twins, I’m not just a wife, not just a daughter, a sister, I’m also myself. [silence] And I’m not just a victim**”.”  **Mother 1:** “I **don’t necessarily like playing mother […]. Well, I knew however what I didn’t like. And I was not ashamed to say it anymore [smiles]. I know I have the right to don’t like playing mother. Period**.”  **Mother 4:** “With perspective, I now see that I fell relatively rapidly, but I went back up relatively rapidly too. And then, I adjusted to this new life. I think that A [her child] did not suffer too much of it, it did not last very long. The moments I couldn’t care for him did not last.”  **Mother 3:** “now when we receive friends at home, we receive them during the boys’ nap so that we could have this sphere a bit… a bit like it used to be, and then the boys wake up and we’re all together…Well, that’s much louder but at least we had that moment, that is essential for me and that makes me live as me, a woman, a full individual and not just a mum, just a wife, etc.”  **Father 1:** “Well, everything helps actually. Her above all, and the providers, the association helped, and without realizing it, the return to daily life. Work, because first I like my work, that’s simple because you return to work, you start taking rhythms. That’s new rhythms, aren’t they? Well, you know it before having a child, but you don’t know how you’ll absorb **it**.”  **Mother 3**: “I found that job in a very small 40-persons company, people are all nice […] And I tell myself, “it was worth doing all that to find that back, that do well” [smiles]… in fact, I’m able to tell you all that because I see there is now a rise in energy and positivism that comes back in my life and I tell myself, “the fact of spreading it will confirm me in what is happening”  **Father 1:** “I can pretty well identify the beginning, the end […]… maybe after 4 to 5 months, you know? With some kind of return to daily life in fact, you know?”  **Father 1:** “Actually, there is already a word on it. That’s dumb, isn’t it? That’s the provider who used it, because you know at the beginning, you don’t really dare to use it. Is that the good term or not? Is it legitimate to talks about baby blues or not? […] And it does… without noticing it, it does good too. Because you tell yourself that’s too important to make a big deal of it… how to say it? That’s too important to say, “maybe that’s not that”. There are some who are really suffering, if you can avoid taking their place. So that was important. […] Well, I knew I was unwell, but then you tell yourself, “is that that?”. Yes that legitimize**.**”  **Mother 3**: "She told me that she thought it was postpartum depression with post-traumatic stress related to childbirth. But she told me that there were already some factors that could have been identified before that actually happened, including my sister’ death, the miscarriage. And she said to me "having twins is a more favorable ground for depression". So she put words to that."  **Pediatrician 2 [male, 74 years old]:** "these traumas are often not told at the time of the delivery and all, but very often when I see them again a long time, even years later, when you unroll and pull on the thread, well then there is the whole ball of yarn that comes, comes, comes. [...] at some point there is a kind of small psychological or psycho-affective opening in the mother and on the occasion of a behavioral disorder in the child, there is an absolutely incredible unwinding. [...] and then too, you have to be there and take the blow"  **Mother 2:** “I went to see that psychologist, and in fact that changed my life. With perspective, I regret not having done it before, but surely that if I did it wouldn’t have been the right moment, so well. And she helped me… to unravel that childbirth, to remove the… Because in fact, deep inside me, I kept some guilt all that time. They may well tell me that what I lived was horrible… In fact in my head, that was my fault since the beginning. I was the one who couldn’t […] thump the table, saying, “that’s not normal, where is my baby?”  **Mother 2:** “**very quickly I really felt better in general… in my relationship with my son. […] But in a few weeks that story that he preferred his father was over. He only said, “mum, mum, mum”. That really unblocked something. I felt much more competent. I had the impression to reinvest myself. That something that completely collapsed was rebuilding differently. And I found myself again**.”  **Mother 2:** “I told myself that in fact during all the time I had the impression to screw up from beginning to end, for him [her son] that was ok. That took place between me and me, but for him that was ok.”  **Father 1**: “And even if things weren't going well on my side, I still wanted to... it was worth it in the end (…) As it happens, I never felt guilty. Because as I was telling a bit earlier, I think that seemed worthwhile, to be treated.”  **Woman serious mental illness (SMI) 2 [22 years old]:** “my biggest resource is my partner. […] That’s someone on whom I can count. I know perfectly well that if I’m at the bottom and that I need time, he will make sure I got some and that’s obvious.”  **Father 1:** “Well, when I say, “I found a way out”, that’s more, “we found a way out”.  **Psychiatrist 4 [female, 35 years old]**: “depending on the father’s involvement […] with the baby and the perception the mother has of the father’s involvement. […] Because many patients coming for postpartum depression tell many difficulties related to that, with a partner they perceive not that involved in relation to the baby, or even involved but not taking a role in organizing things, very passive in fact. That’s really quite a recurrent reproach, anyway for many of my patients, towards their partner and consequently the feeling of loneliness.”  **Mother 2:** “he went to a great daycare […] I felt that he was safe there. So to me that was already… let’s say**… I think that it gave me a positive perception of me: I found a good place for my son. […] There were providers, who without really saying the words, felt, I think, what was happening to me and knew… anyway who made a lot of efforts to valorize me as a mother. […] They had a real role in the fact that it became better”**  **Mother 3:** “the bond with the babies built itself in the course of consultations at [medico-psychological center] too and home visits. The childcare nurse came regularly at home. […] And when she came, she went for the bottles, putting them to bed and then we took a coffee together and that was our one to one moment**.** So there was some kind of rituals too. The Maman Blues association gave me the contact of an association that sends home help or TISF [technicians in social and family interventions] at home. I had a TISF during 3 months. […] She was incredibly helpful too.”  **Psychiatrist 4:** “indeed in peripartum depression, you’ve really the impression that for some patients, the context plays a big role in the appearance of depression. […] in these patients, that’s putting in place some helps or reworking the organization of daily life, for instance by bringing PMI [maternal and child protection] follow-up, TISF, helps like that or by clearing a bit things at the family level, in general that leads to improvement, of course combined with other types of care.”  **Mother SMI 8 [40 years old]:** “We had a midwife too, we had before teams of midwives and childcare nurses. You shouldn’t hesitate to ask child and adolescent psychiatrists for help… There were childcare nurses, midwives who can do home visits. Even if we were well-prepared, if we did all needed to be done, that’s reassuring to have someone else who says, “well, that’s good, you did well”  **Mother 3:** “I consulted a psychiatrist and a childcare nurse. […] they were very present, they did home visits during the lockdown to see the babies, they brought a motor therapist to give us leads on how to awaken them. I could speak without any taboo, without any fear of being judged.”  **Mother 3:** “**And I tell myself, “if I generate something positive and if tell my story, that will make things grow and that’s how it will help other people too”. And the association Maman Blues, they’re on Instagram and all Fridays they offer a mum’s testimony, anonymous or not, on ten slides. And in fact, I did it a couple of weeks ago. And the fact of reading it and viewing the comments… the true really nice comments below, that gives strength** and I told myself, “actually, that’s great to have this network”. And just for that, the associations, that’s… I found it magical because that’s the first lead of help I could put my finger on.”  **Mother 3:** “I had this help from the CMP, I had help from Maman Blues. And I could participate to several video meetings where I was the one who could advise several mums. So, the fact that there was this retroactive loop, that confirmed me in my role. […] And I told her, “in fact, I had the impression to be torn into pieces. That I was really crushed, that I was just pieces and that I had to put the pieces back together as I could”. So postpartum was hard. Today boys are 19 months old and I could die for them while before I wanted to die because of them, that’s too weird [laughs].”  **Mother 2:** “I told myself, “maybe that’s the moment to enroll into an association”. Well, that’s the good alternative between finding a job and staying at home. And well it started like that. And in fact, a job occurred very rapidly and by chance near my place, so I grabbed the opportunity, and now I’m doing both.”  **Mother 4**: “That’s why I posted a testimony at Maman Blues too. Well, I would like to do something of all that” |
| **Informal or formal peer-support: Repairs parent identity / facilitates recovery** | **Mother 2:** “**that’s mentoring that first saves women.** […] A connecting tool, if there could be a forum… so that women support each other. Because you don’t necessarily have someone in your relatives who lived or is living the same thing. […] **I see on the forum how that can help women to repair themselves to just know they’re not the only ones going through this. And too, not the example, but knowing that other women went trough that, well, that many found a way out and who are now helping others. That shows that there is an end to the tunnel, that it’s not definitive**”  **Mother 3**: “**I tell myself, ‘actually, they won’t judge me’. And so I tell my story. […] And I feel not alone and I tell myself, “damn, there is an opening door, I have to take it”. And I hear many mums helping me. There is even one who offers to come help me with the bottles, etc., although she has two very young children. And I tell myself, “that’s it, you put the foot in a network … and that's them who will bring you solutions in fact. Currently, that’s not health providers”.”**  **Midwife 19 [female, 60 years old]:** “And it was enough if one or two mums told about their 1^st^, told what she went through, and that, I really saw that it echoed in others and that was far more eloquent than what I could say because that went from another mum”  **Midwife 21 [female, 38 years old]:** "peer-support groups but also groups with providers within the structures [...] So that they are not isolated with the impression of being stigmatized in relation to a possible peripartum depression."  **Father 1:** “And then [EM, president of Maman Blues], what I appreciated was something like that, “well, yes, in fact you know you’re not alone”. And I told myself I wasn’t the one who was the most feeling sorry, because indeed when you hear about postpartum depression, you tell yourself that could last long**.** […] having someone listening, that in fact there was a dialogue. That’s it. [rises his fingers] The big difference is that you don’t talk to a health provider, that’s… […] something between peers. And that really played a big role. That’s someone who really took time and… first in writing and then by phone”.  **Father 1:** “Well, good network, testimonies, that seems to me the two big strengths. And maybe persons who’re listening too. Well, people who are listening, that comes with testimonies but for after. For people who are affected. Having not only providers, but also persons like [EM, president of Maman Blues], that’s precious. […] We did not discuss the same things. She explained well that she was not a provider… and that, that’s precious. Having both. […] I think that associative mutual help, […] that can work well because that’s selfless, that’s sincere, that’s my opinion anyway. […] And there is one barrier less, that’s not a provider. […] sometimes we can discuss other things or lighter things. […] Knowing how others experienced it. What I appreciated […] that’s that she repeated, “what happened for me doesn’t mean it will be the same for you”. The few moments she told about her experience… well, that allowed knowing that others experienced it even if maybe we’ll not have the same solution. […] That’s someone, as it happens, who doesn’t only share her experience, she talked to other people, mostly mums who went through this. […] As I was advised, I arrived through the forum of the association. And that’s where you realize that there are many, many persons who all live it in their way but…”  **Father 1:** “I want to put in the spotlight the work of both providers and EM [president of Maman Blues] contacted me very rapidly. […] That’s quite a giving of yourself. Because when she took me on the phone between noon and 1 p.m., for instance, during one hour… She also has a life. Well, you see, that’s dumb, but well, […] that should be valorized. Associations like that… well, quite a job!”  **Psychologist 2 [female, 40 years old]**: "expert patients or things related to sharing experiences that was not uninteresting." |
| **Personal recovery in SMI**  Motivating factor for recovery  Facilitator in making decisions about starting a family* | **Mother SMI 7 [31 years old]:** “**Well, when you’ve got an illness, in particular, a mental disorder, […] in the positive indicators, there is also, let’s say, the desire to have a normal life. So being able to have projects: getting married, having children, well… Starting a family**”.  **Mother SMI 5 [30 years old]:** “As long as you have adequate follow-up and treatment, you’ve the right to live an ordinary life like everyone else.”  **Women SMI 6 [36 years old]:** “yet I think that I’m willing to fight. And that illness proved it. […] Well, I think that can’t be seen anymore that I’m bipolar and taking medication. […] I evolved and managed to repel the illness in a little corner where it’s quiet, hidden, but… I think I have big personal resources.”  **Woman SMI 9 [31 years old]:** “I know that I’m doing more and more small steps towards more autonomy, more knowledge of my illness, and all that that can lead me to more stability.”  **Woman SMI 4 [37 years old]:** “**I rely a little on myself too. Because I think I asked myself enough the right question and now I feel ready. So I count on me too**.”  **Woman SMI 2:** “I know myself very well on my mood disorder. I know how to detect it, I know when it’s coming, I understand it well too, so I can speak about it relatively well. So, well, that’s a good resource. […] In fact, spirituality helped me to bear life, and to bear it, to understand it and even to take what’s positive. For sure, that will help me.”  **Woman SMI 4:** "I think about a possible relapse because I would no longer be on treatment, but I am well enough supported to not worry about it anymore. I know that’s one of the risks but that don’t worry me anymore. Because I (...) could talk about it a lot with health providers, so that's a concern that I put aside. That was there in the first place, and that I could manage." |
| **Person-centered recovery-oriented in peripartum health services**  Switching from a risk reduction approach to woman centered care promoting self-determination and empowerment during childbirth and in the postpartum (take into account mothers’ preferences and experiences; discrepancy between health providers’ and mothers’ experience of childbirth)  Shared decision-making about disclosure to other health providers  Individualized peripartum care and support  A movement initiated by mothers  Anticipation / preparation for a 2^nd^ pregnancy | **Mother 3:** “**And this job is to give back to the woman her place in the childbirth. Because that’s the woman who is at the core of the activity. Health providers are for me necessary electrons that gravitated around her, but the core is the mother**”  **Obstetrician 2 [female, 32 years old]:** “That’s a topic that goes well in the spirit of the times. We liberate women’ speech, either at the level of violence, traumatic childbirth, care-related violence. So I find it goes well with all that. **We try to take care of the patients globally, as well in terms of medical care, psychological care, lived experience and I find all that very interesting**”  **Autistic woman 3 [32 years old]:** “**And maybe at the time of childbirth, that your wishes, let’s say, could be respected as much as possible […] with variations if there is an emergency or whatever and depending on the course of childbirth, but that there would be a guideline with the wishes that should be respected as much as possible**.”  **Mother 3:** “And on the 3^rd^ or the 4^th^ day, I slip my hand to touch these staples, and in my head, I think, “actually, that’s the symbol I was silenced”. And I tell myself “that’s awful to think that”, but that’s actually what I think. So at one point that means that something happened to make me think this**.**”  **Mother 3:** “the feeling of infantilization was during the hospitalization because they were telling me what I had to do with the babies, they did not consider me as a decision-making adult; and that was also when returning to work because they dictated me what I had to do.”  **Autistic mother 1 [26 years old]:** “I would not like there would be some things I’m forced to do and that I don’t feel like too. I’m the one who takes the decisions that should be taken. I don’t want someone to call the child protection services for nothing”  **Autistic woman 5 [33 years old]:** “during pregnancy monitoring, I’d like that the mother would not be considered only as an uterus. Meaning child’s health, and also maternal health that would be taken into account.”  **Mother 3:** “that she would be active in her childbirth, […] she’s active in the action because her body is supposed to do things, but the way it’s done, actually she has no power at all.”  **Mother 3:** “maybe to stop saying you should lie on your back when giving birth, with the feet in the stirrups. No, there are many other ways to give birth that are much more comfortable. That’s also the period before childbirth, the control of contractions”  **Mother 3:** “that’s her moment. And not the moment of the team. […] Actually, she’s the one making choices, and she’s the one deciding how things will go on […]. That she would have control because that’s her moment of life”  **Autistic mother 4 [35 years old]:** “I don’t think there should be something “mandatory”. Well, offer to persons, not that she could “dispose” because doctors aren’t’ service providers, but that the person could feel a little free in her care.”  **Mother 3:** “Maybe to inform the persons before childbirth, on what type of childbirth they are entitled to. For instance, a friend of mine gave birth in another hospital, they gave her the choice of the position during childbirth. Well… I find it very important because that’s the person who is at the heart of the action, and yet, at the moment of the act of childbirth should be realized, well, actually, they wipe out all the person’s wishes. I also have a friend […] she was asked about her birth plan […] they took her choices into account, and at the time of childbirth, they totally put that aside and did things according to their protocol. And she felt too that her speech was totally wiped out at the moment of her daughter’s birth.”  **Mother 3:** “Actually, this picture is societal, that’s putting the mum in a context where “feel happy because you had two children, there are some people who can’t have any”. That too… Well, that’s enough! That means that once again our word is turned down”  **Midwife 19:** “on transmissions […] to be able to ask the patient on what she wants us to tell or not to tell […] but at the same time […] a woman who says, “I don’t want you to tell anyone”, that’s extremely difficult to handle.”  **Midwife 18 [female, 64 years old]:** “We tell them, “we call with your approval” […] that still happens and often that we come with them in the consultations […] to create that trusting relationship”  **Pediatrician 1:** “there is some kind of filter that is necessary. […] Mums are willing to by the way. What we systematically do, that’s when we have some information a bit complicated, we ask the mum if she’s ok or not for that to be shared or known by other people than us, and possibly in which conditions. And to assure them that if she’s not willing to, that it’s sure that will not come out.”  **Woman SMI 9:** “and how to disclose it to the obstetrician. How to tell him I take medications that can have an impact on the fetus. I don’t know how to tell. Because I think I will. But is that my doctor who does it or me?”  **Midwife 12 [female, 54 years old]:** “Well, I would say that **just sitting down at the bed of a woman and simply listen what she has to say and free the speech, it allows settling down things a little. […] Also about bad experiences of childbirth,** and when we make the effort to come back seeing these women and just listen what they have to say… **we sometimes aren’t on a equal footing: we experienced it well because we have a baby who is doing well, but this woman had a very bad experience of her childbirth. And to listen them, you realize that that’s already a lot**.”  **Mother SMI 5:** “the hospital where I gave birth, […] we’re numbers, and we give birth, and… the baby is in good health and, well, everything is ok. Unfortunately, there is not… not enough proximity, empathy from health providers”  **Mother 3:** “the anesthetist, so nice, tells me in which position I should be. He puts his forehead on my forehead so that I can push with my forehead if that hurts during the injection. We’re holding hands in fact. I had a very connected moment with that anesthetist.”  **Autistic woman 7 [33 years old]:** “Well, that’ll remain on the human side, that providers don’t think they know it all and that’ll work with everything, everyone.”  **Mother 3:** “There is XX [a midwife on social media]. I’m completely fan of this woman, who is in the spirit of the times, without taboo, she tells it frankly. And she dares saying that she was abusive. And I have the impression that that’s there was that movement too, towards providers becoming aware that they’re not always as perfect as they should be. She actually signed a petition for “a woman, a midwife”. The fact nobody was in my delivery room for hours, well, that lacked. Even if only for chatting, asking questions […] giving elements to the woman so that she can envisage something real”  **Mother 3:** “Well, actually, we should stop idealizing pregnancy. When I was told “don’t worry, it will be vaginally”, well I trusted blindly […] I told myself “that was what I’ve been promised, actually that’s not what I’ve been offered”.  **Mother 2:** “**I think that change comes from patients once they’re able to take hold of their experience and to transcend it**”  **C&A psychiatrist 3 [male, 49 years old]:** “I think health providers will change too because the general public will talk about that. […] Depression is a theme that has been popularized for 2 years. […] Women come more easily. […] Women come for that reason and talk very spontaneously about […] suicidal ideations. Or women we screened and finally who are not cared for and call back telling “I know I have your contact details, I’m not going well at all, otherwise I’ll throw myself through the window, I have to come seeing you”. […] I have the impression, now that there is a movement. […] I don’t feel it was so spontaneous five or ten years ago, anyway, so supported by service users”  **Mother 2:** “**I had a strong desire of a 2^nd^ child and a 2^nd^ childbirth too. I really desired to live things differently. […] So my pregnancy started very well. Let’s say that I was preparing my childbirth like you prepare a siege. […] Actually, I told myself that maybe I couldn’t do much on how that will happen in terms of context, but what I could change was how I would experience it**.”  **Mother 4:** “**the thing is to know how to communicate without being scary. But I still think that us, the women, we’re entitled to the truth, to know, to be in the knowledge, and that when it happen to us, see, I better handled the 2^nd^ time than the 1^st^**.”  **Mother 4:** “and well, that’s true you know that’s a taboo topic, but I had no shame at all anymore […]. And when I felt I wasn’t going well, I told myself, “well, you’re not going well, you know what to do now, don’t do like for A […] well, get supported”.”  **Mother 2:** “In fact I had a great childbirth. B [a friend midwife] was there all along. My husband was there all along. It happened like I wanted to. […] it started at home, we arrived at the maternity, the midwife was very nice, B. arrived immediately after. […] And I found myself, well, there, with my husband at the same place [the place of her 1^st^ childbirth], the monitoring at the same place… the difference was that there was a friend near me to hold my hand, but that was really… with perspective I had the impression of the pastoral of the nativity figures of Provence. Ad the end of the pastoral, all the nativity figures freeze eternally around the crèche. I had the impression that was that […] the nativity figures reawaken. We found ourselves in the same position, at the same place, in the same room and the story plays again. And happened what had to happen, the labor stopped immediately. […] And actually, I had really the impression to be alone but positively alone. I don’t know how to say it, but in fact powerful. I felt… I was there and I was deciding. I decided that what was happening was a good thing. […] after a while I… well apparently I did not tell them and so much the better, but in my head I yelled to everyone to shut up. […] I pushed as if my life depended on it, exactly as I wanted to. […] I let the room to that baby so that he comes into the world. That was incredible. Frankly, that was the craziest experience of my life. […] And after that I felt very powerful for weeks.”  **Mother 2:** “B. [her friend midwife] told me one day, I’ll always remember that phone call, “listen, I don’t know if your husband could be there, but I would. Whatever happens, I’ll be there. Day, night, on shift, not on shift, I’ll be there. You can consider it as granted. I organized the care of my daughter everyday around your possible childbirth and I’ll be there”. And in fact, that saved me”.  **Mother 2:** “it was a great meeting, that’s an extraordinary woman [her psychologist]. […] that was very nice and I think she really was part of my path to get better. […] I really had the feeling to meet the good persons in that pathway.”  **Mother 2:** “I had the impression to be supported by a line of women. […] I had the feeling to be part of something big and to accomplish something big and I really discovered my power at that moment. And so, surely nothing was the same. Postpartum was much simpler”  **Mother 2:** “I relived my childbirth under hypnosis. […] So I really could relive my childbirth, adding something positive… she made me verbalize the fact that… […] I did the best I could. And I really went out with, “actually I did the best I could with the weapons I had at that moment, with the help they offered me”. And that was great. Actually, that was a miracle that my son was going well, that I went through this like that, given what could have happened”.  **Mother SMI 7:** “Well, the resource persons, there is my husband who is there, because he lived all the difficult moments before, and my parents because they live at ¾h, one hour from our home, so they were very present during pregnancy, and very present after childbirth. They came almost every day to support me because I was not that well”. |

*Theme identified only by non-parents
