# Supplementary material for: Toward recovery-oriented perinatal healthcare: A participatory qualitative exploration of persons with lived experience and health providers’ views and experiences
Source: Eur Psychiatry. 2023 Oct 20;66(1):e86. doi: 10.1192/j.eurpsy.2023.2464 (PMC10964275; doi:10.1192/j.eurpsy.2023.2464)
Supplement: Dubreucq et al. supplementary material 6 — Dubreucq et al. supplementary material [file S0924933823024641sup006.doc]

Supplementary Table 6: Quotation supporting the theme "From a fragmented service provision to a graduated joint parent-baby care**"** (**bold** figure in Table 3)

| **From a fragmented service provision to a graduated joint parent-baby care** | |
| --- | --- |
| **Investing in peripartum mental health (PMH)**  Lack of training in perinatal psychiatry  Need for perinatal psychiatric services  Needs extra-time to address PMH issues  Increasing the duration of the stay at the maternity / improving continuity of care after discharge | **Psychologist 3 [female, 60 years old]:** “she went to a psychiatrist […], “it’s not going well, with my baby. I’m not feeling good”. But I think that given this psychiatrist was not trained to this issue, told her, “well, everything is going well”. So her request was rejected. […] Because she went to the right place and was not listened.”  **C&A psychiatrist 3 [male, 49 years old]:** “There is the issue of access to care, and the problem is that the basic skills of adult psychiatrists and child and adolescent psychiatrists do not allow them to develop an activity because they don’t have enough volume and thus they don’t have skills and… at the end that remains undetected. The lack of basic skills results in forgetting these situations. […] Adult psychiatrists don’t know the resources and they don’t have the sensitivity. So, well, they monitor them otherwise or they don't monitor them.”  **Mother serious mental illness (SMI) 7:** “Psychiatrists in general, they don’t know that much.”  **Psychiatrist 2 [female, 34 years old]:** “Or they say, “oh, still that’s very particular to evaluate a mother who just gave birth or during the peripartum” […] while we’re in a service where that’s something they bath in. […] However, they really have the feeling that that’s something that needs very specific skills and they prefer avoiding it.”  **Psychiatrist 2:** “that rather support an over-expertise, but still that says… something about an inadequate anxiety in frontline evaluation. […] When, you’re in the screening consultation, you just say, “well we should prevent the disaster”. You just have to be sure that indeed there is no overwhelming symptoms. […] Well, you see what is very delimited.”  **Psychiatrist 3 [female, 32 years old]:** “I work in child and adolescent psychiatry [C&A psychiatry] but I’m the only adult psychiatrist. I’m exclusively affected to the maternity and replacing me is a huge problem. […] So when I’m not there that’s very complicated and I … Well, I understand it as a great anxiety from my C&A psychiatrist colleagues because they’ll have do deal with mums and that’s very technical. […] Meaning, yes, that’s very specific so that demands to be equipped for that specificity, and at the same time, sometimes that’s accessible too and I tell myself, “in fact, we could know how to do the minimum”.  **C&A psychiatrist 3:** “There is an issue with mental health providers’ skills, who at the end see too few babies, in particular in C&A psychiatry to avoid… they see many adolescents, a few 6-12 years, very few babies. So they don’t have the time or the resources to develop specific skills. And it we stay in a “secteur” logic, that will not be able to correctly develop itself.”  **Psychologist 8 [female, 30 years old]:** “indeed the colleagues in C&A psychiatry and all that, don’t see enough people to, well I think, unless by personal interest, to develop that skills.”  **Psychiatrist 2**: “Yes, and similarly in adult psychiatry. Adult psychiatry, anyway, there is a rejection of mothers’ peripartum care saying, “oh no, that’s a mess, I don't know how to do it, that’s complicated. There is a baby in the belly so psychotropic drugs I don’t know, the interactions, I don’t know, that’s very complicated”. I say that when I teach classes, “interactions, that’s first looking at what is happening.” […] There is also some very basic things that are within the reach of anyone. But as says Psychiatrist 5, when you’re not regularly confronted to the situation, you’ll not participate in training, rise your skills on that topic, because at the end that doesn’t correspond enough to your activity.”  **C&A psychiatrist 3:** “Recently, a melancholic woman hospitalized in our own hospital, followed-up for her pregnancy in a hospital with which we work regularly, well, nobody told us before childbirth. Never. Everything was to be built. She lived 100 meters from us. They never thought to contact perinatal psychiatry. Why not? That’s repeated, that's regular”.  **C&A psychiatrist 3:** “the problem of screening, that also belongs to the woman who found hard to express that kind of problems, to insufficiently trained providers”  **C&A psychiatrist 3:** “Maybe that's thanks […] to the training. Because after all, that’s better than the liaison psychiatrist who does rheumatology, endocrinology and then goes at the maternity to say, “she’s not delusional, she’s not suicidal, so I’m leaving”. That’s not at all what is asked.”  **Psychologist 6 [female, 39 years old]:** “we’ve been confronted to a gap between what we can detect with our education as childcare providers and adult psychiatrists, who indeed don’t share the same concern as us for the baby”  **Psychiatrist 2:** “historically, perinatal psychiatry has been developed by C&A psychiatrists in France, adult psychiatrists grab it more and more, which clearly poses questions, even problems and even conflicts, for many people and finally I find that what is great currently, that’s that the younger clinicians, I have the impression, get out of that conflict, and hear that we need multiple skills and that everyone has a role to play. And that the option of perinatal psychiatry will be opened to the DES [diploma of specialized studies in psychiatry] of adult psychiatry and C&A psychiatry, I think that’s really very good and a good way to answer to the end of that division that is obviously detrimental. I agree with Psychiatrist 5 on the fact that **peripartum care should not take place in adult psychiatry as well as in C&A psychiatry. Really we should have dedicated places at maternities for perinatal psychiatry. And we see that the services offering that type of care are more effective**.”  **Nurse 3 [female, 45 years old]:** “We indeed work in pairs, meaning doctor-nurse or psychologist-nurse, and that indeed allows to have that global perspective on the situation. Meaning that we’re not focused on the baby or the mum or the dad, we’re really on the bond, the relationship, parents, even the family. […] That’s another way of caring.”  **C&A psychiatrist 3:** “finally you’re talking about two things that are connected but also slightly different, that are access to care and the organization of care. The organization of care, finally what we’re saying is that it’s important to have joint perspectives…”  **Psychiatrist 2:** “crossed perspectives”  **Mother SMI 5 [30 years old]:** “Well, at that moment, my fear was mainly about the medication. But I was lucky enough to have a very good psychiatrist who asked […] a psychiatrist trained in peripartum to have a treatment compatible with pregnancy and to have the less withdrawal syndrome as possible after childbirth.”  **C&A psychiatrist 3:** “skills in adult psychiatry, skills in interactions and that’s better if we’re two to embody it. That’s not mandatory, but that seems more consistent because that embodies each role.”  **Psychiatrist 2:** “it’s no big deal if the persons taking in charge haven’t all skills. Meaning that if there are adult psychiatrists who aren’t specialized in taking care of bonding or babies, if there are C&A psychiatrists who don’t know how to handle psychotropic drugs in pregnant women, that’s no big deal if there is a service into which there are multiple skills that are mixed and that we can refer to the colleagues. And I think that it’s really more and more the model of perinatal psychiatric services as they’re conceived”  **C&A psychiatrist 3:** “The 2^nd^ point I was thinking about was the issue of referral. I’m very, very sensible to that. I agree on the huge loss between liaison psychiatry, work inside maternities, and care services. To me, that’s not in adult CMP [medico-psychological center ] that they should go, that’s in perinatal psychiatric services. That we don't have enough, that’s not very obvious yet, but that’s where they should go.”  **Psychiatrist 3:** “sometimes I tell myself that the purpose of perinatal psychiatry is to raise awareness in others on what that could be and to play things down by saying, “well, indeed that can be accessible just by thinking a minimum about it, and after, of course that needs technical skills and we’re there to answer to that.  **Father 1 [34 years old]:** “It was a psychiatrist. […] What helps there is that there is an environment a bit for parents but also for children, meaning there is not a lot of white cloaks, you see what I mean?”  **Midwife 12 [female, 54 years old]:** "Sometimes at the maternity, we had patients for whom we called liaison psychiatry and, in fact, we had the impression of not being heard. Meaning they trivialized [...] I would say that they come rather easily but that we have the impression that [...] when we call them, [...] we are not listened and we have a bit the impression to be let down.”  **Midwife 12:** "They told us that was normal, [...] but we found that, besides that, there were red flags, things that really worried us. [...] after 2 years, she is still accompanied by the PMI [maternal and child protection], by the TISF [technicians in social and family interventions]. [...] we put quite a lot of things in motion, and there is a complex support [...] We could talk about that situation with the perinatal psychiatrist who told us that she was indeed worried too for these children.”  **Pediatrician 1:** "there is also a complete time gap! Meaning that the temporalities of one another are not at all the same. [...] Really there is some kind of temporality where we detect some great suffering and an emergency situation, which in reality maybe isn’t.”  **Midwife 3 [female, 42 years old]:** "that’s important to emphasize the fact that psychology and psychiatry during pregnancy is a specialty in its own right and we need to rely on a network of C&A psychiatrists and psychologists who have this perinatal training, with whom we can, in fact, exchange more easily because indeed, there is a problem of temporality."  **C&A psychiatrist 1 [female, 52 years old]:** “regarding […] family interviews, that’s to see things from the side of the child, and in particular baby. Because if the baby is not there, you can rapidly interpret how things are going with the baby.”  **C&A psychiatrist 3:** "For me, who practiced for a long time in children’ CMP, the frustration was to see children coming for symptoms that you a posteriori identify as a history of maternal depression. That’s what motivated me to do perinatal psychiatry, that was to try to be very, very early on."  **Midwife 18 [female, 64 years old]:** “I indeed sometimes orient to psychologists. As my colleagues said, that’s much easier to make them admit and accept that orientation. But when they’ve previous history, interrupted medications, when they already had a follow-up by a psychiatrist, that’s the moment when we propose that orientation to a perinatal psychiatrist. The fact of saying “perinatal psychiatrist”, they accept a little more that orientation.”  **Psychologist 2 [female, 40 years old]:** “For instance, in a service for pathologies of pregnancy, I think of course to the baby in this mother’s belly, but my patient, that’s the mum. In a pediatric ward, […] that’s the baby […] even if you take care of the parents.”  **Psychologist 8:** “there is that specificity of peripartum care. And when you work in peripartum care as psychiatrist or as psychologist, you’re interested in adults as much as in children. You should indeed have that double set of skills and that’s true that this clinical care is very particular.”  **Psychologist 6:** “there is indeed the question of trained psychiatrists, because that clinical care is very particular.”  **Midwife 3:** “that takes time to detect, to coordinate and to refer and that’s not valorized at all, and that is a pity.”  **Obstetrician 1 [female, 62 years old]**: “to tell health providers that sometimes that’s by moving a little beyond your role – and that time has to be acknowledged for private and public providers - that it works.”  **General Practitioner (GP) 2:** “when they come, we have many things to see with the baby, and finding the time and the right words to chat with her and then try to catch her. […] I fear of missing it. Meaning that when in half an hour, you have to know how things are going at home, how he sleeps, how he eats, how much he took, to examine him from head to toe, well sometimes we just don’t have the time to know how the mum is going.”  **Pediatrician 2 [male, 74 years old]:** “when I receive a father in my cabinet, I need 30% time more.”  **GP1 [female, 58 years old]:**”But that takes time, so you need availability. And at the PMI, what we also ask for, that’s to have more time to spend with these mothers and these babies.”  **Pediatrician 3 [female, 47 years old]:** “We don’t always have access to that, maybe also because we don’t have much time to ask people, the time of consultation is brief: 30mn. Anyway, we have a specification that is quite important during our consultations, in the sense of we still have to do a certain number of things and maybe we don’t always have the space to go further.”  **GP3 [female, 52 years old]:** “We have more and more to do parental reassurance and for my 1^st^ consultation either at Day 15 or at the first month, I always schedule a long 30 or 45mn consultation to try conducting a 1^st^ assessment of the mum.” (p3)  **Midwife 5:** “we don’t have the same functioning in private practice and in public hospitals. We’re more easily reachable. I got messages… […] late at night, the weekend when you can’t contact anybody, etc. This kind of care takes much time. […] that work should be acknowledged. Because that takes much time but at the same time that’s so rich.”  **Obstetrician 1:** “that’s the time acknowledge, you’re right, that’s the time spent. Because we realize that all midwives don’t take the same time, don’t do the same and sometimes that’s also voluntary work. And these psychologically vulnerable patients […] ask time to everyone. And we could ask that it could be acknowledged.”  **Midwife 17 [female, 36 years old]:** “Anyway, backing this there are midwives who are there and take time with patients.”  **Midwife 14 [female, 45 years old]:** “So as health provider, there are many themes like that that we should discuss. That takes time.”  **Midwife 26 [female, 52 years old]:** "Exactly. Hospital times are getting much shorter and it's true that, in my opinion, we are missing out on quite a few situations. And there was a problem of identification [...] it's difficult to clear up things in hospital in immediate postpartum."  **GP1:** "I would like to react to the length of time spent in the maternity ward. It is that when we are aware of some more fragile situations, or at risk, with sometimes multiple problems, where we have the impression of playing the carpet merchant to negotiate a sufficiently long stay and a quality observation time."  **Mother1:** "We stay what, 3 days in the maternity ward? (...) But, yes I would have liked to have a midwife, come on, every day for a week. It would have been great I think. While she came, what? Twice a week?"  **Midwife 15 [female, 47 years old]:** "stays are getting shorter and shorter "  **Pediatrician 1**: "But the time spent in the maternity ward, [...] you still have to be very quick. [...] . And in this, we have to find the warning signs if they were not there before.  **Psychologist 2:** "It's easier in antenatal care because it gives us a little time whereas in maternity care you don't have much time... Well, yes, you have those discussed with the PMI who are identified beforehand..."  **Midwife 12:** "we try to do this prevention in any case, but it's not always easy because we sometimes don't have the time to see everyone [...] the stays are very short"  **Mother 1 [33 years old]: “**The PRADO [“support program to get back home”] was great.”  **Mother 1:** “I think it may come from the “PRADO”. I find it really great […] she almost did not come enough”.  **Mother 1:** “my midwife made me doing perineal reeducation even though I did not need it, extra, to make me go out”  **Midwife 26:** “And maybe a local network of providers, well, local resources.”  **Midwife 24 [female, 35 years old]:** “To inform also on home help. […] the welfare office help […] for hours of housework”. |
| **Improving continuity of care**  Continuity of care between pregnancy and postpartum  Continuity of care between hospital and other actors involved in peripartum mental health  Continuity of care between peripartum and psychiatric and mental health providers  Role of the psychologists integrated to maternity wards or neonatology services  Clear and transparent communication / trusting relationship between mental health providers and other health providers  Continuity of care between liaison psychiatry and perinatal psychiatry  Provision of communication tools between perinatal and mental health providers and parents  Updated directory of resources available on the area | **Pediatrician 4 [female, 48 years old]:** “**we don’t see those babies, but that’s true that our level, regarding hospitalized babies, I think there are really things that should be improved in the connection obstetrics / pediatrics.** […] An obstetrician called recently because he saw a mother at one month and said, “damn, that’s not going well”. I told myself, “wow, that’s the 1^st^ time I have this phone call for I don’t know how long”. Unbelievable. He was young, particularly sensitive […]. That’s good he did that.”  **Midwife 1 [female, 51 years old]** “we often ask ourselves about postpartum depression during the next pregnancy. […] There is a period of emptiness for women in postpartum between the end of the private practice midwife’s care and the 1^st^ appointment with the GP. There is a moment when we’ve the impression they have no monitoring. Perineal reeducation is over, they don’t see their private practice midwife anymore and they’ve contraception, they return to work, they haven’t seen their GP yet and thus there is no one at this moment and that’s when it happens. And so that poses the question of the continuity of care.”  **Midwife 24:** "On the private practice side, it is true that we have the advantage and the strength of knowing our patients well when we do the follow-up, the preparation for birth. And it's true that when we do the PRADO follow-up, so the home visits, it's all in comfort because we know the patient. The difficulties I have in these cases that’s when I go to the home of a patient I don't know."  **Pediatrician 4:** “That’s more that I already handed over the reins. I would rather say that’s good to be alert to signs that will be beyond the usual in neonatal care, for us during the hospitalization. […] And the connection. I would really see an enhancement of connections, because that’s really a problem […] the connection between obstetricians and pediatricians, or midwives and pediatricians, that’s everywhere. […] That’s as if it was a ditch. There is childbirth, and before and after. And that will certainly pose a problem”  **Midwife 27 [female, 34 years old]:** "I receive letters for patients that I never saw and they explain to me that I was supposed to take over. [...] No appointment was made and they did not contact us. [...] And we are not necessarily aware if there was a contact with the PMI liaison in place."  **Midwife 8 [female, 35 years old]:** "we discuss a lot with the PMI midwives, so we manage to find support solutions for mums who have difficulties and to solve these… well, to support these mums. So we work closely with the PMI midwives and the private practice midwives.”  **Midwife 27**: "For the city-hospital relay, I think it would be interesting if we could have "postpartum" meetings, I don't know, to discuss some cases where we got limited so that we can all coordinate better and understand... we need to understand the limits in the hospital and we also need to understand our limits at home. [...] It's true that I would love it if we could have more postpartum meetings from time to time, to see what happened to that family."  **Midwife 9 [female, 41 years old]:** "We have a small-scale functioning, but that works very, very well with the PMI. We have regular meetings on situations [...]. We manage to create a network”  **GP1**: "with the hospital psychologists we make connections, we call each other. [...] We contact the person’s GP, we try to refer.”  **Midwife 10 [female, 53 years old]:** "But that's mostly the city-hospital links."  **Childcare Nurse 1 [female, 28 years old]:** "At the PMI, I only receive mothers who gave birth. Often the hospital gives us liaison sheets"  **Midwife 18:** "what’s essential is that there is that someone continues intervening: the private practice midwife at home, the health executive at the maternity ward.”  **Pediatrician 1:** "we work with a C&A psychiatrist of the University Hospital, knowing that I know her very little, and that’s true that it’s a link we don't have much, but our psychologist works closely with her."  **Midwife 3:** "that's also important to rely on the health providers already in place. And what’s important is to be able to identify the resource person for the patient, who can be the private practice midwife... and we must not substitute ourselves for this person. We must support the patient while leaving in 1^st^ line the person she designated.”  **Midwife23:** "**there is that Taskforce project [...] the PMI joined it. And in fact the goal [...] was to create a care pathway for women who might already be identified but who could also be referred to us after a first consultation because the midwife would have detected warning signs [...] there would be a midwife identified for the follow-up of psychologically vulnerable people during pregnancy.** She would work in pairs with a psychiatric nurse and within this unit there would be a psychiatrist, a psychologist and an obstetrician as well. [...] These staff could take over in the postpartum period to explain the situation to the teams [...] In fact the ultimate goal of this project [...] would be to avoid having too many placements."  **Midwife 1:** "We either have women who have follow-up, regular treatment, with medical care, and most of the time that goes pretty well. Which allows us to coordinate with a private practice specialist, a C&A psychiatrist, a psychiatrist or a private practice midwife, or a general practitioner."  **Childcare nurse1**: "psychiatry does not necessarily mobilize immediately [...] So there was an emergency placement of mother and child. [...] the mother did not accept the psychological follow-up, nor was it put in place at the placement level. [...] and this led to the placement of the child when he was 2 months old because we were no longer able to handle this mother and this child. [...] We could have avoided the placement if we had worked beforehand"  **Psychologist 6: "**the networking we do [...] I work closely with the PMI [...] she calls the childcare nurse because she feels completely overwhelmed, and the childcare worker tells her to call me and then I answer her on the phone and I call the childcare nurse back.”  **Pediatrician 4:** "We should have a home treatment team of C&A psychiatrists. [...] I think that there is a way to do things in the framework of pre and post immediate perinatal care. [...] And we could imagine that in these risk situations we could alert and send someone, either at home or to a specialized consultation. [...] on the other hand, there would have to be something organized to be able to refer them."  **Midwife 9:** “On the big situations that posed us questions, we finally work a lot with the psycho-perinatal team where the same, we work directly.”  **Midwife 1:** “So I didn't have any difficulties, it was done in a very fluid way [...] I went to the essentials, and there you have it, a C&A psychiatrist was needed, thus some care”  **GP1:** “I was once obliged to go to at home to have a mother hospitalized [...] I came across a psychiatrist on duty who was very supportive, what. And we were able to bring this mother to the emergency ward”  **Pediatrician 1:** “in intensive care [...] mums are systematically seen by the psychologists of the service. [...] We share things on the field. Meaning mums need a space that is also preserved, with the meetings with our psychologists, and I try, as a resuscitator to not put my feet in this field [...] there is a division of tasks [...] what we expect when contacting doctors [...] is news about the child’s health.”  **Midwife 12:** “from what our psychologist will say, she’ll either […] continue proposing to follow her up after or refer her to liaison psychiatry if she thinks that’s more worrying.”  **Childcare Nurse 2 [female, 26 years old]:** “As childcare nurses, we already have a listening role to the parents, because that’s true that we will sometimes detect situations that are a bit fragile. [...] What is interesting is that we have psychologists who intervene quite regularly, [...] who will take over when we have a doubt and who will have a space to speak that is distinct from ours whereas we’re more involved in daily care. And so they have a very different perspective. And that's really complementary, I think. And that allows us to see these parents who can pose problems”  **Pediatrician 1:** "our psychologist has more or less integrated the neonatology service coming from C&A psychiatry. So she has a real culture and knowledge of C&A psychiatry."  **GP1: "**to negotiate a sufficiently long stay and quality observation time. We try to negotiate this when we discuss situations with the antenatal parenting staff with the maternity units. Hence the importance of having a psychologist in the maternity"  **Pediatrician 1:** “We also try to operate on the hospital. So when we involve the psychologists of the hospital with whom I work the most at the service level... that's true that we try to mobilize the network, already to see wether that's possible that the mum returns home.”  **Childcare nurse 1:** "If the mother already saw a psychologist at the maternity hospital, for example, we can redirect her to the psychologist she already saw to try to establish continuity of follow-up. [...] We also easily refer to an association where there are psychologists [...] to the home treatment team."  **Midwife 3:** “**psychologists and psychiatrists having pregnant patients shouldn’t hesitate to come to us. In Valence, we are lucky enough to… I have many psychiatrists calling me, saying, “Mrs. Whatshername is pregnant, I refer her to you”. And that’s true that this trust is agreeable, and that works both ways in fact.** They shouldn’t forget to refer pregnant women, early, during pregnancy.”  **Midwife19**: "at some point I'm not going to be able to help you anymore. I think it's important to have another provider intervene. [...] my colleague who is a psychologist and will know better how to help you. [...] I am a midwife [...] the fact that we communicate with each other, I have the feeling that it reassures them. [...] Each one in our place but working in the same direction. Each one with our own skills and specificities."  **Psychologist 1 [female, 49 years old]:** "I think that in fact all the actors of the peripartum [...] are important and need to trust each other."  **Midwife 18:** "we work with a whole network. We try, with the childcare nurses and the different partners with whom we work, to catch and orient them, and above all to alert"  **Midwife 26:** "Thus, we indicate the relays, we give small things, we possibly do a liaison with the PMI [...] we orient to parent-child support groups, to meeting places for parents / children that exist. [...] After, well, will people take hold of this?”  **Midwife 6 [female, 59 years old]:** "That's not the same to have a list of names and to have a list of referents, concerned, trained, motivated. [...] I need to know people, and when I entrust a woman to someone, when I know him [...] the link is much easier to make and much more useful. [...] does he do that? Is he there? Is he available? "  **Psychiatrist 3:** "This means to come back to the provider, saying 'thank you, this referral was good because of this', or 'well, this could require that'. Well, [...] liaison psychiatry has that exciting and exhausting aspect that in fact you spend an incredible amount of time talking with colleagues, and in fact... [...] in my case I embody perinatal psychiatry and thus in fact I’m potentially responsible... well, I can positively influence on the fact of people would be aware, would be reassured, as I can negatively influence… that’s not simple.”  **C&A psychiatrist 3**: “we have a very specific model that poses problems, that’s that liaison activity is very disjoint from the usual care. If one day we want it to function well, persons in liaison should have a foot into care so that it could circulate. Not simply because that’s the same persons who will take in charge, but because they’re concerned by what happens later, they’re involved in family outcome, that’s not simply an orientation.”  **Psychiatrist 2:** "Our model is that, in concrete terms, the liaison psychiatrists at the maternity hospital are the perinatal psychiatrists who will receive and follow up on the patients during the year. So we have one foot in and one foot out, even if geographically everything is inside, but we have this liaison and consultation-follow-up aspect. And it is certain that it works because there is continuity and it is very comfortable.”    **Pediatrician 1:** "a two-way patient-doctor channel [...] also to have a case manager who could answer to some question while alerting the doctor, the PMI."  **Autistic woman 5 [33 years old]:** "a tool shared between all health providers, so that they could communicate together on the person's health status. I think that would be interesting because there are things that you tell your GP that you don't necessarily tell our psychologist, and vice versa. And things that the GP detects and that would be of interest to such and such a person. A tool like that would be interesting to have.”  **Midwife 3:** "could a mobile application ensure that there is a private practice midwife, that there is a general practitioner because sometimes they declare a GP, but in fact they don't know him, or they don't have any to make sure that there is a real follow-up in the long term. That there would be no gap."  **Pediatrician 1:** “Which means that there would be a part common for patients and providers, and there will need a provider part for just that speed of access.”  **Autistic woman 2 [22 years old]:** "that could be a place where peripartum and mental health providers… different providers serving as a resource during pregnancy or after. Even before for that matter."  **Childcare nurse 3 [female, 37 years old]:** "maybe also a part for providers where they could discuss together. [...] to avoid the mother or father having to re-explain the context several times"  **Psychologist 5 [female, 41 years old]:** "**patients should have access to available places of care, available working time [...] be received without too much delay, being received at a pace that suits them**"  **Psychiatrist 3:** “The big difficulty we all have […] trying to cartography perinatal psychiatry care. And that’s discouraging because actually, depending on… not even on the department or the region, that's depending on the 2km^2^, […] Actually cartography all that’ something of a headache because that depends very much […] of the local willingness of each one. So a person who’s leaving that’s enough for the service to fall apart. […]”  **Mother 1:** "Who to contact? Whom do you contact to if you don't feel well?"  **Midwife 5:** "a reference directory of people to contact in case of emergency."  **Psychiatrist 3:** "It is one of the key points. That’s coordination. Coordination, networking and identifying it and making it live because that never stops because that changes all the time in fact. We have great room for improvement there. That helping us to improve that, would be clearly welcome."  **Midwife 17:** "I would take a geolocation of professionals who are around the patient's home or around my practice. [...] to see what is available"  **GP1:** "the directory is, who to consult? Where? That's to have a panel of choices too. [...] And the cost too."  **Autistic woman 2:** "it could take the form of a folder with contact numbers, or explanations, information."  **Mother 3 [35 years old]: "**The associations are still... I found that magical because it was the first help I could get my hands on (...) They gave me the contacts of all the others. In fact, I left the maternity hospital with a paper that listed the possible helps. So "if you have a problem, don't hesitate to contact the pediatricians in the area". So I contacted all these pediatricians but they didn't have room, they weren't taking new patients. So for the first month visit that was already complicated during the first month, but even more so with doctors without any availability. There was also the number of the PMI, which was obsolete, for the record. They had changed their number a year ago. In fact, that's to say that even when they want to do things right, well, in fact... just check if the numbers are right. I don't think it takes forever. And that's really important. So there's that. They didn't even explain to me that there was a CMP next door if I ever needed it, I never heard from the psychologist from the ward, even though she was supposed to come back to me, nobody knew about... There was even a nurse who said to me one day "but have you seen the psychologist from the ward? Well, I never saw her". "Oh yeah! Okay! So we're actually at that point!?" "  **Midwife 13 [female, 40 years old]:** "To put in some contacts. Whom can the mother contact if she needs it?"  **GP2:** "A phone book?"  **Pediatrician 1:** "if we say, there is the directory, but there are also people on shift, whom to call before 8 and from 5pm"  **Psychologist 2**: "a directory?"  **Psychologist 1**: "Local resources"  **Psychiatrist 2:** "I don't know if that's possible, but if there could be a "I live in such and such a place, well, who can I contact" thing. And why not just a generic thing, just, well, you have the PMI, you have the CMP, you have the CMP for children, but Ok, you live in such and such a place, well in this place, the person best identified to respond to your needs in perinatal psychiatry is going to be Mrs. Bidule, who is a psychologist in private practice at such and such an address with such and such a number, and she is going to be the one most likely to be able to respond to your problem in the area. That would be great!"  **Autistic woman 3 [32 years old]:** "They are not very visible, but I believe that there are PMI, where one can get information, where one can get sound advice. (...) But yes, a place where you can seek advice. I'll also have to find a pediatrician, but it's true that for other, broader advice... Yes, that would be more what I'd be interested in, a resource place we'll say, where we can ask our questions."  **Autistic woman 5:** "Maybe a list of providers to contact with contact information"  **Midwife 14: "**a peer-support group [...] by geolocation, by sector they can find each other."  **Midwife 1:** "since that’s often a secretary or a nurse who picks up the phone in psychiatric or psychological care services, so, anyway having the names and phone numbers of staff members” |
| **Facilitate the development of a common culture and language**  Development of a common language  Organization of joint training sessions on peripartum mental health  More knowing each other place and role  Multidisciplinary work facilitate the development of a common culture  Need of feedback from mental health providers to peripartum providers  Supervision from mental health provider to peripartum health providers  Use of secure online communication tools, allowing a rapid and transparent exchange of information between the different providers | **Midwife 8:** **"I think that what is important is that we could all speak the same language, so effectively assessments grids...** and that we could all base ourselves on validated grids"  **Midwife 3**: "I think that sharing a common language would be helpful."  **Pediatrician 2:** "**there is probably a lack of some kind of common training**."  **Psychiatrist 3:** "It turns out that I am much more involved in obstetric services than in the C&A psychiatry service. And that the assistant specialist put recently me on training classes for obstetrical residents, for medical students (...) she said, "we have to ask one question because perinatal psychiatry is very important" (...). People who contact me are my obstetrician colleagues. So, maybe I'm optimistic, but I say to myself "oh, that's not bad"**.** That’s pretty good. Yes, that’s pretty good because indeed that’s from them, among others, that everything will start”.  **Psychologist 6**: “a place where we can share information, knowledge."  **Psychologist 2:** "It depends on our exchanges with them [the midwives], or their training. They are willing to know the symptoms and how to handle postpartum depression. [...] I had feedback from this group, requests and training expectations."  **GP1:** "**the childcare nurse during the home visit [...] the link with the GP. [...] it requires knowing each other, saying things to each other, knowing each other's place**."  **Midwife 12**: "Maybe knowing our interlocutors better [...] have meetings once or twice a year"  **GP1**: "Learning how to know each other better, could it go through common trainings so that we could know each other better? Regular meetings, peripartum meetings that are open. [...] Hospital pediatricians tried to come from time to time. [...] If the hospital's C&A psychiatrists could also come to these staffs from time to time, I think that would make it possible to establish a link, to discuss situations, to give feedback..."  **Pediatrician 1:** "We know today the psychiatrists even if we don't see each other much, but maybe we would really need a peripartum meeting with psychiatrists, which would also allow it to be something less virtual."  **GP1:** “simply by naming things, by not hiding things, by being rather in the dialogue with families and hospital services. […] the possibility to tell things to each other and to explain it to the mum, the dad, sometimes to family relatives so that they could be involved too, not feel put aside. And also to try to reassure to move forward. But yes, that needs a tight partnership and to respect each one’s place, that’s not always easy, and to know well what the ones and the others do and what are our skills, or ways of doing things too.”  **Pediatrician 1:** "maybe we would really need regular peripartum meetings with psychiatrists, [...] and maybe a discussion platform”  **Psychologist 2**: "in the “pathologies of pregnancy” service, we have meetings with obstetricians, pediatricians and midwives”.  **Midwife 16 [female, 50 years old]**: "I think that we should have more often that small meeting time between providers”  **Midwife 19 [female, 60 years old]:** "all that networking [...] You shouldn’t be alone."  **Father1:** "And a good territorial network of this healthcare service, that was listening. I was referred to the right person in two seconds."  **Pediatrician 3:** “the field knowledge of local health providers.”  **GP2**: "Continuity, yes."  **Midwife 5: "In Valence there is a culture about this. There was a very involved psychiatrist, well, who left the area, but there was really joint work with the maternity team."**  **Midwife 21 [female, 38 years old]:** "that there be a sort of multidisciplinary follow-up. And in multidisciplinary that's not just gynecologist, pediatrician, midwife that’s the psychiatric side too."  **Pediatrician 2:** "that kind of interdisciplinary work is absolutely exciting [...] You have to put a midwife in, a pharmacist, etc., because the pharmacist’s role is crucial too, in that listening. Because many things, many confidences are told. And we cross all our perspectives. Of course, there is a psychiatrist involved. And that’s something that makes connections between providers, and we all use the same signals.”  **Pediatrician 1:** "So the interest of the psychiatrist [...] that’s precisely that he will take care of this and step back and say: "we will look at this with a different eye"."  **Pediatrician 4:** “That’s more that I already handed over the reins. I would rather say that’s good to be alert to signs that will be beyond the usual in neonatal care, for us during the hospitalization. The mental health staff, well, that’s very, very rich, and I think that’s really what the psychologist sees, but also what we see and what the nurses see. That’s really, as we said, the cross-fertilization of perspectives that is very important. And to have someone immediately available, still, that’s really precious**.** Well, you can’t replace it. And the connection. I would really see an enhancement of connections, because that’s really a problem […] the connection between obstetricians and pediatricians, or midwives and pediatricians, that’s everywhere. […] That’s as if it was a ditch. There is childbirth, and before and after. And that will certainly pose a problem”  **Psychiatrist 3:** "It is one of the key points. That’s coordination. Coordination, networking and identifying it and making it live because that never stops because that changes all the time in fact. We have great room for improvement there. That helping us to improve that, would be clearly welcome."  **Pediatrician 2:** "that kind of document [a brochure on PMH Disorders], you should absolutely have an interdisciplinary redaction, otherwise, we’ll not see it coming, as always, that will not be used. The proof is that algorithms for identifying depression, finally, are not used. So that’s a pity."  **Pediatrician 2:** "I also have the chance to work with a team that is not far from here where there are psychologists, motor therapists, speech therapists, neuropsychologists, so there is a private practice network too that led me to create this network"  **Childcare assistant 1 [female, 32 years old]**: “on the peripartum and obstetrics [...]. We really handle the issue of children who are alongside. Maybe psychiatry is not well aware of that, maybe the patient’s condition in itself is not necessarily catastrophic in their terms compared with other patients they have. Anyway, we’ve got this issue of family’s and children’ safety. [...] her situation is concerning because she has kids to handle. And you can’t put these children on the hold. [...] That adds a specific concern. [...] they consider that these patients aren’t that serious"  **GP1:** "You can do it when we are several to handle the situation. That's when I find that what's important is to have crossed perspectives"  **Pediatrician 1:** "that really requires to be several thinking together”  **Midwife 25 [female, 60 years old]:** "Well, then that's teamwork, we try to have several perspectives. Already during the pregnancy, with the hospital colleagues, we try to have meetings [...] We are lucky at the PMI to have psychologists."  **Obstetrician 1:** "the care network, the midwifery network, that’s a long story in Valence, and (...) our child psychiatrist (...) waved that work on the importance of a midwifery network, trained the providers to screening, and did all this underground work."  **Pediatrician 3:** “And then, there is the question of the culture of connection. That’s often when there is a space with a private practice psychologist, well, they say, “that’s the closed space of the consultation, nothing goes out, that’s with the parents”. Except that thus we lose that network which is relevant.”  **Childcare Nurse 2:** “As childcare nurses, we already have a listening role to the parents, because that’s true that we will sometimes detect situations that are a bit fragile. [...] What is interesting is that we have psychologists who intervene quite regularly, [...] who will take over when we have a doubt and who will have a space to speak that is distinct from ours whereas we’re more involved in daily care. And so they have a very different perspective. And that's really complementary, I think. And that allows us to see these parents who can pose problems”  **Midwife 21:** "postpartum hemorrhage is a leading cause of mortality, we realize that… well, that’s with us within the service. **Whereas suicide occurs finally weeks, months, or even in the first 1000 days, well, in the next 2 years, and when you are a hospital midwife, you will never be aware of it. And so that’s invisible for us. [...] but that we could have some kind of feedback on whether there were perinatal deaths in the first two years. I don't know how that could be possible, but to be aware**"  **Midwife 13:** “I had a bit the feeling to be isolated regarding what was being done for that patient. So having a feedback from the psychologist or the psychiatrist following her up would have helped me to care for her. Anyway, would have been professionally more rewarding.”  **Midwife 18**: “When they’re followed by psychiatrists, that’s very complicated to have a feedback”  **Midwife 13:** "I would have liked that there would be more coordination between providers. And to be able to have information, when we have referred, to have a little feedback so that the patient sees that we are communicating with each other."  **Midwife 14:** "we don't get feedback on the degree of concern of the psychologist or psychiatrist [...] sometimes we also have this lack of feedback"  **Midwife 10:** "I didn't hear from the psychiatry department at all afterwards. [...] communication with the team that took over. Then it was me who asked for news, so I knew how it went […] a focus on communication."  **Pediatrician 3:** "And then there is the question of the culture of the link. That’s often when there is a space with a psychologist in town, well they say "that’s the closed space of the consultation, nothing that comes out, that’s between the parents". Except that, as a result, we lose that networking that is relevant."  **Pediatrician 1:** “that’s true that this discussion and the feedback too… Well, there is the secret and we don’t need to know what the mum discussed with the psychiatrist, but to tell, “do we share that worry?”. There is a feedback, saying, “well, where do we go now?” And that, that’s a bit mysterious. To avoid saying all the times.”  **Pediatrician 3:** "when we refer them for example to the CMP we don't really work with them. That is to say, we screen but we don't really get any feedback from that. So we can't really work with them. We only work on the basis of what the parents bring back to us, we are not networking in fact. It's as if there were two spaces, with no communication. So is that necessary or not? Is that the parents' wish? Is that the problem of the time we have to talk to each other?"  **Pediatrician 4:** "that's a real problem because if you don't get feedback you can't improve either. Meaning that if you’re not told "well no, in fact, the mother was fine" or "you didn't see it but she wasn’t well at all”, how could you use it for the next times? You won’t know how to do better.”  **Pediatrician 3:** "So I don't know what they do because they don't contact me, I refer them, but then they take care of them and then the parents come back and they're pretty happy, so I feel like that's handled."  **Midwife 2 [female, 38 years old]** “Well as private practitioners, our big concern is that we have very few feedback, that’s I who had to call them again. I had to ensure that the connection with the PMI had been done. […] Well, much time, worries.”  **Midwife 6:** "The APP [analysis of professional practices] is also an interesting moment, the discussions we have together, that we are lucky enough to have in Valence, that we had before Covid, that allows the private practitioners, instead of remaining alone, to discuss with psychologists, psychiatrists on concrete situations as they come. And that's super important to analyze situations more finely and effectively."  **Pediatrician 1:** "we systematically exchanged around the table with our psychologist on situations where we had concerns"  **Midwife14**: "to have someone referent, to whom we could expose a clinical case, and who could guide us a little bit in the way we can take care of it"  **Midwife 2**: "we have supervision with a psychologist every month and a half/two months. So we could establish together what I needed to monitor, what I needed to do with this woman before came to see her."  **Pediatrician 3:** "we don't have any space to take another look on things. That’s true they give you an emotional load or a suffering load, and, well, sometimes you don't know how to deal with it. You come back with that at home, because you perceive something but you don’t have any space to take another look at it. So there should really be something that could exist, because that’s a network I think. And that has to be waved, thought and elaborated.”  **C&A psychiatrist 2 [male, 28 years old]:** “That’s also the link we can have with the team. Meaning, as mental health providers, what link we could have with the persons gravitating around that situation. And taking time to listen to them, to try to understand their representations and to train them.”    **Obstetrician 1:** "Using SISRA, which is a secured texting tool, that’s an amazing tool for midwifery settings. Because connections can be made very quickly, very easily, on a secure medical messaging service"  **Pediatrician 2:** "One of the solutions, regarding the relationships between midwives / obstetricians, / general practitioners / pediatricians, we have for example the SISRA network. [...] either more or less formal exchanges, that especially are not direct, that are indirect because we are not obliged to answer immediately, I find that’s one of the solutions. On the other hand, that's true that it should be better organized."  **Pediatrician 2:** "A document that would be shared, meaning that it’s made by health providers of all sides, but also female service users, or male service users why not. And then, I imagine an arm where indeed, according to the signs and symptoms detected by parents or providers, someone refers to a referent. [...] all the more because midwives reproached us…. Apparently, every woman has a referent midwife when leaving the maternity I didn't know that existed. But at the limit, maybe she’s the most easily identifiable provider, and to me, there are still some midwives. [...] GPs aren’t there anymore, pediatricians even less. So I imagine two stages to that, we’ll not bother the referent for a yes or a no, so it has to be co-written and co-directed"  **Midwife 3:** "we have a shared obstetrical file that allows exchanging with all stakeholders, in particular private practice midwives, but not uniquely: also the HAD [at home hospitalization] referent midwife for example. This allows exchanging information in a fluid, rapid and efficient manner, almost in real time in fact. We are lucky to have regular meetings with private practice midwives, once a month; with PMI midwives once a week and we work in conjunction with a mobile C&A psychiatry team and a perinatal psychiatrist. And we meet this C&A psychiatry team on a weekly basis. In addition, we have a tool called SISRA, which is a secure medical messaging system, and when we’re confronted to difficulties when caring for a patient, we generate a SISRA, and we integrate the different medical actors involved in the situation as we go along, which gives to providers some flexibility in their exchanges. We can read these SISRAs when we have time, and we respond when we have time. Each actor has his own place we really complement each other. You can also leave a SISRA conversation when you’re not the right interlocutor or when your role over, so to speak. We are also lucky to have a team cell phone. So patients, who are in a vulnerable situation, especially psychologically, can contact us or midwife 4, or me. They know that we are there from Monday to Friday, that they can call us for whatever reason and we can tell them that we can’t answer, but that we will contact a provider who may have the answer. This gives them security. They know we are accessible, we're available and we're connected to each other."  **Midwife 5:** "That's an absolute comfort to have the mobile number of a psychiatric team, to be able to send a SISRA message to the gynecologist following the patient when that's not the midwife, or when it's E [a midwife colleague], because she does coordination."  **Pediatrician 3:** "that's always the problem of the link between providers. Meaning that we have a child health record booklet that is ultra-filled, but we don't have a messaging system, we can't contact each other. As a result, everything is based on our own observation of the families, but if, for example, there are things that we didn’t see but that the midwife saw, well, you won't know. [...] there is not really a care network. [...] if the mum is depressed or if there is some tension in the couple, that will never be written in the child health record booklet. So you don’t know that. And we don't have the culture of contacting each other to talk about this."  **GP1:** "We also work on the basis of the liaison sheets collected from the different maternities or pediatric or neonatal services. So that too is a tool that for us is also important to have red flags and to say, well, you should be vigilant."  **Psychologist 2:** "I appreciate receiving email feedback, or having exchanges to sometimes share elements, but... That would be like a shared medical file?" |
| **Development of a graduated referral algorithm based on the needs of the parent-child triad and the degree of emergency**  Discrepancy in assessment of severity / feeling of isolation  Referral practices depends of available resources  Graduated referral algorithm  Graduated joint parent-baby peripartum mental health care  Joint obstetric and psychiatric care (USAP / preconception consultation) | **Psychologist 2**: “midwives will indeed be more alerted by some things… but because they have only three days ahead and that there is a lot of activity, but they are more alerted, that’s true, more alarmist. While in pediatrics, [...] there is less judgment"  **Childcare assistant 1**: “on the peripartum and obstetrics [...]. We really handle the issue of children who are alongside. Maybe psychiatry is not well aware of that, maybe the patient’s condition in itself is not necessarily catastrophic in their terms compared with other patients they have. Anyway, we’ve got this issue of family’s and children’ safety. [...] her situation is concerning because she has kids to handle. And you can’t put these children on the hold. [...] That adds a specific concern. [...] they consider that these patients aren’t that serious"  **Childcare assistant 1:** “They said that’s not that serious [...] that’s often not very well taken into account. They think that’s not that important, that the maternity, that’s not something serious, that that can wait. [...] We are a bit left out. [...] they minimize things a bit."  **Midwife 8:** "that’s true that we face big difficulties in getting appointments with psychiatrists. The temporality, the notion of emergency differs between the clinical situations we can see and the appointments they can offer. And I find that’s that is out of step with what we could observe on the field. I find we have difficulties with that. We manage to get psychiatric assessments but I find them completely out of step with the clinical situation we encounter. That’s more that kind of difficulty."  **Midwife 12:** "even with a psychologist supporting us, we could be very worried and then, we were not necessarily very well backed up”.  **GP1:** "Sometimes, you find it difficult to make your worries heard [...]. Coming to psychiatric emergencies was complicated. Very complicated. I would say that's very person-dependent."  **Pediatrician 1:** “The discrepancy in concerns about the situation”  **Pediatrician 1:** "a mother who arrived in intensive care [...] who had a major acute decompensation, for whom we contacted all the phone numbers that existed in psychiatry, and for whom we were told that this kind of situation was never urgent. And there you are with a mother for whom you are afraid for her life, you are afraid for the baby, you feel totally helpless, and you face someone who tells you with a disconcerting serenity: "you know, you have to contact the service X or Y"."  **GP2:** “even depression, not at that stage, that’s an emergency but not within one day. But when you feel a sense of urgency, that’s because I could be afraid for the mum or the baby.”  **Midwife 2:** "I receive a call from my colleagues on Sunday to tell me that she's going to be discharged and that they don't feel she's doing very well psychologically or even psychiatrically but that the psychiatrist refuses to see the patient because he thinks there's no emergency."  **Pediatrician 1:** “to have dealt at the CAMSP [early medico-social action center] with speechless babies with mums prostrated in an armchair, where it took months and months of support with the PMI until at one point someone decides that this situation had to be taken care of. You can’t leave a child with a mother who can’t get up in the morning! And when you’re told that you’re excessive, and that you should not take all that that seriously! [angry]. Indeed, I get upset in these situations because I see the mum […]. That’s not that she doesn’t want to, that’s that she can’t.”  **GP1:** "In fact, we have the impression to speak a little more the same language and to have the same preoccupations at the same time on both the mum, the child and the family. And to know when to get alarmed, when to take it to the next level."  **Midwife 22 [female, 36 years old]**: “if you choose to orient first to a psychologist, that’s because it’s indeed much simpler. […] rather than mobilizing a psychiatrist which is rather complicated. […] And often, the remaining option is psychiatric emergencies. […] that’s a little more higher level.”  **Psychologist 6:** “today we orient more to the GPs than to the psychiatrists**.** […] That’s important to have medical support”.  **Midwife 18: “**Because we favor the psychologist in first intention. […] firstly psychologist and then psychiatrist. Sometimes that’s the psychologist who decides of this orientation to the psychiatrist.”  **Midwife 19:** “Referral to psychiatrists, I personally find it difficult too. In the network, we have difficulties to find a psychiatrist.”  **Midwife 8:** “there is always this lack of psychiatrists in some areas where sometimes that’s a bit complicated but still we manage it by being patient. And by finding support and monitoring solutions while waiting for a psychiatric evaluation.”  **Midwife 12:** “from what our psychologist will say, she’ll either […] continue proposing to follow her up after or refer her to liaison psychiatry if she thinks that’s more worrying.”  **Midwife 21:”**That’s first the psychologist and then the psychiatrist”.  **Midwife 9:** “that’s finally more complicated when you go outside of the metropolis to look for local contacts […]. There is not necessarily a connection with the PMI because we don’t have any contact”.  **Midwife 17: “**Well, in my small town, there is no psychiatrist. So that, that’s already complicated.”  **Psychologist 6:** “Meaning we all spoke more about the GP than the psychiatrist because unfortunately, […] we don’t have a psychiatrist”  **C&A psychiatrist 1:** "**the adaptability of care depending on patients**."  **GP1:** "Not some guidelines but a decision tree that can guide a little."  **Obstetrician 3 [female, 34 years old]:** "Some kind of score that could show that they belong to a category at risk for depression, and what type of care could be offered**,** what type of care we could offer them, which providers they can contact. [...] rapid answers, people to contact rapidly."  **Pediatrician 2:** "inside there could be, “well, now, you should consult”, the red flag too. And then “consult your doctor or your midwife**”.** But there too, everything remains to be done**.** [...] Well, actually, the treatment depends indeed on the severity level that we determine together.”  **Obstetrician 1:** **"**that’s important to have some graduated care**.** Meaning that not everyone would see the C&A psychiatrist”  **Midwife 3:** "In Valence we use the HAS [National Health Authority] pregnancy pathways, 1A to 2B, and in fact when you graduate the medical pathway of pregnancy, you define whether the patient will see a doctor or a midwife first. And I think that it would be interesting if we could say, "well this woman is immediately the responsibility of the C&A psychiatrist, or that’s first an assessment by the psychologist”. (...) So I think we should draw the parallel with the HAS medical care pathways and how we could assess psychological aspects, the follow-up we could propose to patients.”  **Obstetrician 1**: “the 1^st^ choice that’s the psychologist. And we should indeed… well, put the specialists by level, meaning that a patient… well, that’s the C&A psychiatrists who’ll help us to make this classification, but… really have that base of to whom refer. Firstly, except for severity factors […] women taking antipsychotics, etc. or with clinical severity factors, they come to see the psychologist. And then the psychologist will say, “well, that’s more severe. […] To spare the child and adolescent psychiatry. There are many things that can be solved by a discussion with a psychologist. And the psychologist will detect the patients who’ll need support, C&A psychiatry, longer peripartum care, mother-baby unit, etc.”  **Midwife 8:** "if we had assessment grids, if we could score these patients, with, let's say, a real suicidal risk, that would already allow referring them more precisely to a psychiatrist, to a psychologist."  **Midwife 1:** "I think the starting point is the diagnosis, meaning a referent, who would allow from information transmitted very quickly, would be able to situate us either in an emergency hospitalization situation, or in a delayed one. [...] an aid to the diagnosis, but also on the temporality. Will we refer that patient the same day, to whom should we turn? And if that’s within a week, to whom should we turn? And then, within that timeframe, is it the care of the mum and if so, what do we do with the baby?"  **GP1**: “**When you get to a certain level of depression when the mum is very, very, very unwell, the idea is to separate her from her baby too. […] And to have no mother-baby unit to hospitalize, to work on the bonding despite everything. […] that’s something that always remains painful**. […] we really need a real mother-baby unit there.”  **Midwife 1:** “that's the care of the baby. You can’t ignore the care of the mum, well… What happens to the baby? At some point, we should think about it. Considering that we can have postponed hospitalization and care too.”  **Mother 4 [39 years old]:** "Maybe put some kind of, not a directory, but the different possible care because in fact there are plenty, and you see the mother-baby unit, I wouldn’t have needed it for my 1^st^ experience while at this point that was necessary."  **Pediatrician 1:** “some situations where we see the absence of a therapeutic apartment, of mother-child care, in which we could go beyond simply separating the mum from the child. […] Because sometimes we look a little like persecutors. […] We would like to be able to support mothers with their babies but there isn’t any service here.”  **Midwife 5: “**the ideal is to have mother-baby care”  **Psychologist 8**: “we intend to open parental rooms, and in particular the fact that there would also be the longer paternity leave could allow easier joint parents-baby hospitalizations”  **Mother 4:** “I know that now mother-baby units receive parents too, fathers too, and that’s very good because my partner, was not affected, well he wasn’t affected, he did not have a depression even if he had a little backlash when I came back from the mother baby unit, he was quite exhausted. But he staid the course, let’s say. But I think that in some situations, that’s the whole family that should be taken care of. Sometimes when that’s a first child, the couple is a little shaken, the father can be completely off-balance too”.  **GP3:** "I must admit that as soon as I diagnose them, I don’t have much problems. Either I orient them directly to the network, or if needed I put them on medication, and above all, as a 2^nd^ curtain, there is an inpatient care just for mums at the TEPPE. So I confess that my network is already in place and that’s pretty comfortable for me [laughs]."  **Obstetrician 2 [female, 32 years old]:** “**Joint consultations with a psychiatrist and an obstetrician**."  **Midwife 11 [female, 53 years old]:** "my clinical experience relates to my consultations in prison, with incarcerated women. So, that’s **woman having psychiatric treatments, opiate substitute treatments and indeed we work very, very, very, well with the USAP [early support in perinatal care]. The USAP accepts to receive them during pregnancy and after childbirth they go to the USAP.**”  **Midwife 4 [female, 34 years old]:** "whether it would be the VALERIANE team or the team in general, we work a lot as a network. [...] Well, I personally work a lot at the consultations I also do some coordination work so I can really detect the patients. As midwives, we detect a lot of patients and we can really offer the right team and the right follow-up, meaning that anyway we’ve time with them. So there are several things. First of all, Obstetrician 1 who is very available for everything that is pathological, the psycho-perinatal home treatment team, psychologists, the TEPPE [psychiatric hospital], also midwives offering acupuncture, a whole team. [...] We know each other pretty well and we work as a network. [...] There is really some teamwork here and that’s very pleasant. And for the postpartum, the HAD team anticipates, we also anticipate during pregnancy and that’s true that makes the care easier afterwards**.** And patients adhering to the program are very happy with it. Well, that’s my viewpoint as hospital provider doing coordination work…  **Midwife 11**: "The USAP, that’s really of great help”  **Midwife 1:** "And then we offer care [...], telling them: 'Well, I offer you that type of care because I can see that it's difficult, it's painful. Then you can think about it, and either we get back in touch quickly and set up concrete things...". So in that case I contacted the USAP in the Woman-Mother-Baby Hospital [...] The doctor taking care of her left so she was taken care of in the baby care unit in Villeurbanne. And I put that mother in relationship with a childcare nurse who did home visits”  **C&A psychiatrist 1: “**the connections between health providers and so later the trust between the patient who will refer herself to a midwife and then will have to refer herself to a psychiatrist or a psychologist. […] offering joint consultations”.  **GP3:** "Despite being in private practice, I’m lucky enough to have a psychologist in the cabinet who has experience in perinatal care [...]. I have this support with the psychologist and in Valence we are lucky enough to have a service specialized in perinatal care in the CMP and a whole team that come see them at home [...] and an inpatient unit just for mums at the TEPPE.”  **Pediatrician 3:** "I had quite a few situations that I sent to LAPS [adult psychiatry community service specialized in peripartum mental health care], the Villeurbanne network that handles peripartum mental health care with " |
| **Facilitating access to specialist peripartum mental health care (SPMHC)**  Organization-level factors  Heterogeneous access depending on available resources  From the psychiatric sector level to a territorial level  Facilitate rapid and responsive access to psychiatric and mental health care  Providers level:  Knowledge of available resources  Knowledge of referral pathway  Screening easier for mothers with identified risk factors  Pediatrician position: a barrier to opening discussions about PMH  Screening practices depend on available resources  Parents level:  Discussions not opened by patients (lack of awareness, knowledge about peripartum mental heath disorder (PHMD) or adhesion into care)  Peer-support facilitates access to peripartum mental health care (PMHC)  Relatives facilitate access to PMHC  Chat / forum facilitate access to PMHC  Support groups facilitate access to PMHC | **Midwife 5:** “**there is still a problem of adequate care in postpartum for reasons related to available beds, mother-baby beds, adequate services to care for mothers and children**.”  **Psychiatrist 2: “The big difficulty we all have […] trying to cartography perinatal psychiatry care. And that’s discouraging because actually, depending on… not even on the department or the region, that's depending on the 2km^2^, well there, the care is supported by adult psychiatry services, elsewhere C&A psychiatry, elsewhere services specialized in perinatal psychiatry… there are very developed maternities and others not at all, there are mother-baby units, PMIs, private practice providers, well…. […] Peripartum health network function very differently from each other, we put in place very different processes**.”  **Psychologist 6:** “we’re lucky to have a perinatal psychiatric service we can rely on”  **Midwife 5**: "So HAD is of great help. We also have psychologists who are available. Finally, I think that in Valence we still have a great wealth of care."  **Midwife 10:** "if I consider that it’s not a situation of extreme vulnerability that should be immediately hospitalized, I contact directly the LAPS or private practitioners to avoid hospitalization or hospitals in general. (...) in that center, there are child psychiatry nurses, psychologists and psychiatrists. And they decide within the team which provider will intervene. That’s already something. We’re also lucky enough to have VITAX, a team from the Vinatier hospital [psychiatric hospital] [...] with a nurse going at home. And we worked closely, the ITTAC [Institute for the treatment of cognitive and affective disorders], the USAP inpatient unit [early support in perinatal care] in the hospital, the PMI and myself.”  **Psychiatrist 2:** “the cartography is so dependent of the area covered, and once again so moving. […] Well, there is all that networking, and when the network is created to discover it.”  **Midwife 6:** “We’re lacking mother-baby units […] that’s difficult to care for mothers and babies.”  **GP2:** “the boy […] is going to be 3 years old. And there is no one anymore. […] She accepted that she was unwell. And well, she’ll find herself at the CMP with adult psychiatrists who don’t have training on that. It’s a pity that there is no hospital service that could care for this mums beyond 36 months.”  **GP2 [female, 47 years old]:** “there is not enough staff, that’s not only in peripartum care where that’s difficult to hand over the reins.”  **Pediatrician 3:** “yet, psychologists that’s not reimbursed, so […] there is really the money-related issue of accessing care.”  **Psychologist 3 [female, 60 years old]**: “all that lack of psychologists, […] we’re limited by the number of sessions […] will these parents be able to take over the psychologist’s fee?”  **C&A psychiatrist 3:** “**that needs some thinking, a little revolution of the systems. Some are a bit limited, too delimited, so that needs that we think about reorganizing care in a broader territorial fashion**.”  **Psychiatrist 2:** “Still, there is a difficulty in getting out of the logic of division into sectors, which is, I think, that the need to stay in the catchment area is very important. And the sector is based on that, on the idea that we stay in a catchment area, etc. […] Except that in fact, there should probably be a service provision much more extended on a geographical level to meet a different population need.”  **Psychiatrist 2**: “that's really a change of paradigm”  **C&A psychiatrist 3:** “that's getting out of the logic of division into sectors [a unit of organization of psychiatric care in France depending on the person’s address]”.  **GP2:** "**Having something reactive, and having the possibility that these mums could... when they know they would be able to see someone rapidly, that calms them down much.** [...] If they don’t already have a psychiatrist I usually contact NEBE [association of private practice psychologists specialized in peripartum mental health].”  **Psychologist 1:** "What also works is when I can rapidly, when I need to, have a doctor available if medication is needed.”  **Psychologist 2:** “crisis services at the university hospital that are reactive for our patients. [...] calling back persons who are suicidal”  **Psychologist 4 [female, 38 years old]:** "the links we also have with the PMI [...] at the end places in a daycare were found quickly"  **Mother 4:** “Childcare nurse of the PMI when she came, […] she told me, “by the way if at some point you see you’re not going well, you know we have a psychiatric nurse at the PMI who can come to see you”, and that remained in my mind. So when really I wasn’t well anymore, I told myself, “ok, I see the psychologist, my usual psychologist but that would be good to have someone specialized in postpartum”. […] So I called her and she came. […] And she told me about the mother baby unit.”  **Midwife 18:** "Perinatal psychiatrists. [...] Appointments are more or less rapid when the orientation is motivated. [...] depending on the emergency, you have an appointment within 15 days max."  **Midwife 10:** “For emergency hospitalizations, we’re lucky enough to have a parent-baby facility”  **Obstetrician 3 [female, 34 years old]:** "we needed something stronger [...] why not immediately a psychiatrist? [...] Someone used to treat postpartum depression."  **Obstetrician 3:** “I oriented her to the “Naitre et Bien-Être” association [association of private practice psychologists specialized in PMH] and I gave her the contact details of a few psychiatrists [...] But I was still quite worried. And in fact she didn’t find a psychiatrist receiving her rapidly which put me into trouble. [...] the week after when I saw her, she still hadn’t contacted one. [...] That worried me.”  **GP2:** “I have never had to send a mother to psychiatric emergencies because in cases when that occurred, there was a referent psychiatrist and solutions could be found.”  **GP1:** “to refer to NEBE among others, which is our main partner. We know that it would be very reactive, that it would be quick”  **C&A psychiatrist 2**: "the key point was reactivity, the fact that she already had a trustworthy relationship with the psycho-perinatal team [...] and also the fact that we were able to build up a network quite quickly [...]she had the psychologist, the psychiatrist, who could prescribe medication and the psycho-corporal approach"  **Pediatrician 4:** “So we are very helped because we have resources. And in particular I find that having a child psychiatrist in the team [...] that helps us, because we can see that there are cases [...] where the mother [...] doesn’t bond enough with her baby, and then, we rapidly refer to her if we can, if the mother is willing to, for a consultation. She’s the one making the diagnosis [...] She sometimes treats herself the mums. [...] Anyway, that’s a help, the diagnosis occurs quite early.”  **Mother 2 [36 years old]:** “**and so I found out that there was a lot of resources in our area, including in our city, that already existed when I was struggling. So that in fact, nobody knows. Well, that the providers don’t know, except those who are at the core of the target. But that’s not necessarily them that the women will meet.”**  **Father 1:** “The midwife was a great help, even if that’s by talking a bit with her that she told us, “well, my advice is to consult such and such providers”. [...] we felt lucky to come across the right persons.”  **Midwife 2:** "so I had her general practitioner in between, I had the maternity, I had the liaison psychiatrist."  **Psychologist 3:** "referrals are made [...] either by the PMI, or by the GP, sometimes already by the internal services: the obstetrician, the gynecologist who detect things [...] the midwives, all the providers"  **Father 1**: “My partner thought that we could ask the midwife who cared for us before childbirth and she oriented me to… [...] above all a person who was good at listening. And that did much good."  **Mother4**: "An aunt of mine who is a psychoanalyst (...) told me 'listen, I think that the solution for you is a mother-baby unit'. [...] She staid a few days and on Monday, the mother-baby unit in Alsace called me and offered me a direct admission, without an appointment.”  **Father1**: "For me that was the private practice midwife. She used to work at the hospital, with which she had always kept in touch, and so for her it was obvious to refer me to this center. Yes, yes. So a good territorial network"  **Psychiatrist 5:** “Recently, a melancholic woman hospitalized in our own hospital, followed-up for her pregnancy in a hospital with which we work regularly, well, nobody told us before childbirth. Never. Everything was to be built. She lived 100 meters from us. They never thought to contact perinatal psychiatry. Why not? That’s repeated, that's regular”.  **Psychiatrist 2:** "Anyway, I find that it's really an element of response that really goes in the direction of what you were saying Psychiatrist 5 to initiate the response, that’s that indeed **that should be how to give access to care and to whom to turn, that’s a fairly central element of that.** I think that one of the key elements is information: finding information. So well, information on what is it? What is its prevalence? What are its symptoms? What are its complications? What is the management? Etc."  **Pediatrician 4:** "I refer them rather quickly and the whole team is quite attentive, [...] if there is a situation outside the norm, well refer very quickly to the psychologist or the psychiatrist."  **Mother 4:** “I felt very unwell, and I told her, “well, I feel like being hospitalized. I have to be taken in charge, I can’t cope anymore”. […] I went to my doctor at that moment who was desperate too, who didn’t know about mother-baby units, who tried to contact a local hospital. […] She managed to tell me, “I leave you a message. That’s not a psychologist that you need, that’s a psychiatrist, maybe your treatment should be adjusted”, well the poor was a bit panicking. She left a message to a psychiatrist who called me back the day after.”  **C&A psychiatrist 3:** “Recently, a melancholic woman hospitalized in our own hospital, followed-up for her pregnancy in a hospital with which we work regularly, well, nobody told us before childbirth. Never. Everything was to be built. She lived 100 meters from us. They never thought to contact perinatal psychiatry. Why not? That’s repeated, that's regular”.  **Midwife 13:** "people who are already in a situation of depression or a psychiatric condition, I find that’s quite well framed from the start. For me, the difficulty is more for patients who haven’t slipped into their pregnancy state, as the pregnancy progresses. Either because of a rather heavy history, or because of marital problems, or because of worries about the pregnancy itself, and I don't feel that I have enough arguments to refer them to the psychologist at that time. [...] I don't feel legitimate either [...] in fact, my voice is not necessarily going to be sufficiently heard."  **Midwife 12: "**We also have people who are treated for depression during pregnancy. And on these patients, I think we don't hesitate at all to ask them questions, in fact. To find out how they feel, if they have been able to review with their psychiatrist or GPs"  **Midwife 12:** “For those without prior history, we still will ask ourselves when we’ve patients crying a lot, who are sad, who can’t take care of their baby, when we feel that’s complicated, or conversely who are very, very demanding. […] We see that’s complicated with their baby, who ring very often, who always need to have a provider alongside them, supporting them.”  **Obstetrician 2:** “I found it simpler as anyway it was mothers with risk factors, where there was previous pregnancies that were very complicated or a specific disorder during pregnancy that makes easier to figure these women out. I haven’t got any women, like the postpartum depression which can really be the woman who hasn’t any risk factor and decompensate for you don’t know really why. During the antenatal period, I didn’t have any.”  **Midwife 12:** "We have a lot of peripartum healthcare meetings, where we have patients where we know of previous depression or depression during pregnancy and on these patients, we will be particularly vigilant. The teams will be attentive to the symptoms and how the mother behaves... and already her, how she... her experience of childbirth [...] be interested in the mother-child relationship, [...] if this mother manages to take care of her baby, if she manages to sleep, if she is sad, if she cries"  **Midwife 26:** "families with big problems already clearly identified, who arrive with already PMI relays that have been established or with private practice midwives already established in antenatal."  **Midwife 26**: "You see that some things occurred in the pathway, things that will make them fragile or, during childbirth that a big postpartum hemorrhage or a very poor childbirth experience would make them fragile [...]. You realize that some fragility is installing and you have very little time to evaluate it. [...] You see they are extremely fragile [...] but you don't have yet clearly identified things to pass on"  **Psychologist 4:** “there are those mums […] despite all we already evoked, the feelings of guilt, shame and all that, who can rapidly tell it and alert. […] that’s also a depression that can stay very unnoticed in other mums”  **Pediatrician 1:** "And then fear for mums who indeed disappear. Typically, mums come every day or with a regular scheduled rhythm, and the suddenly disappear from the screen. So you call them, you try to contact the families, the mum, and that usually creates a lot of anxiety."  **Pediatrician 2:** “maybe I lacked punch to go further, because that’s often difficult. […] But I think that probably to grasp and say, “well maybe there is something to do, to take care of”, that’s not always easy, to my mind, as clinician, because maternal depression by definition that’s not the baby anymore. […] I have a memory a bit painful […] of a pediatrician that had been prosecuted by a mother because she told the mother, “well, listen, you should consult a psychologist”. And the mother filed a complaint to the medical board saying, “what business is it of her?” Sometimes the role of the pediatrician is not always easy.”  **Pediatrician 3:** “I stay in my role of pediatrician because you can’t be at all places too, well, I think that’s not easy.”  **Pediatrician 2:** “that’s mainly through the child’s eyes that I have the impression to find this situation. Additionally, I was always very interested in all what is non-verbal in the child and in particular analyzing the crying. […] I find that one of the best actions, that’s indeed prevention.”  **Pediatrician 3:** “I don’t know if I know well how to detect maternal depression. What I detect more, that’s suffering peripartum situations, […] either coming from the child’s behavior or from the complaints of the parents around that behavior. […] Babies crying a lot, who are inconsolable, who don’t sleep…”  **GP 3:** “Maybe that’s easier for me because I also wear a GP hat, so well, I’ve the two hats so anyway, that’s simpler to detect early depressive signs”  **GP 3:** “I see them much later, and then I’m not at all connected with the mother, first, because you’ve a lot of questionnaires to answer for the child, so I confess that I don’t care at all of the parents…”  **Pediatrician 3:** “sometimes you learn one year after that they’re separated […] There is the visit at nine months, “did something important happened in the child’s life?”, and well, you may well ask this question, they don’t always answer, as if we weren’t authorized to be informed on that.” (p9)  **Pediatrician 3:** “what’s important is to be there, in the continuity. But you can’t always be at all the places. And I find that sometimes when you ask too much the mothers, at the end, you’re a bit intrusive. […] I had several unfortunate experiences during which I think I went too far in my investigation and when, actually, that even generated some kind of rejection.”  **Pediatrician 3:** “I’m relatively surprised of what they’re willing to tell us and what they don’t want to tell us. Because maybe you don’t dare to ask the questions, but sometimes too you’re assigned to a role, and they don’t necessarily want us to leave it. And I find that sometimes that’s necessary to respect it. Because I tell myself that if I asked some questions that are too intrusive, in a way I wouldn’t respect the role they give to me.”  **Pediatrician 3:** “I wouldn’t know to define if that’s maternal depression, if that’s paternal depression, but anyway that’s something which often appears as not very harmonious in bonding and thus that’s not always simple to take things by the right angle.”  **GP2:** “when in half an hour, you have to know how things are going at home, how he sleeps, how he eats, how much he took, to examine him from head to toe, well sometimes we just don’t have the time to know how the mum is going.”  **Pediatrician 2:** "So that perspective is crucial to me, and I believe I’m willing to share this knowledge [...] and this observation, what we call informal knowledge. And this transmission of informal knowledge is colossal and that's why this finesse of observation, and then of course of examination, is very, very instructive for me. And I can tell you that every day, i can feel if a mother, by her attitude is rather zen, etc."  **GP3:** “I see them much later, and then I’m not at all on the mother, again, because we have a lot of questionnaires to answer for the child, so I confess I don’t care at all of the parents…”  **GP3:** “You were talking about pathways, I think that’s to detect and get people back, but **what do we do for referral after? […]** They ask us to do much detection, I fully agree, but we don’t have a 2^nd^ step afterwards**. That’s the problem. The more tools you have, the more you detect but after there is nothing behind. […] that’s currently the problem of my job,** you detect and then you can’t do anything with that.”  **GP2: “**That’s to have an answer to provide to families”  **C&A psychiatrist 3:** "If you build that from the scratch, in a maternity without any care, liaison psychiatry, you’ll just create unsolvable problems for providers. Well, I think that there should first be a well-established liaison psychiatry, a collaboration and that will allow to reinforce something. I’m not sure that could be the 1^st^ way to start a collaboration between psychiatry and the maternity.”  **Psychiatrist 2:** "Either by lack of resources [...], “Your screening system is great, but we don’t have anything behind, so, in fact, we can see that some are suffering, but we don't know very well to whom to refer them, etc.", so there is also that difficulty, that finally they only come when they’re really in extremely severe cases and so that’s a bit late… too late."  **Psychologist 8**: "I think that’s done, but, well that’s a little a bit on an individual basis. Meaning that some maternities will do it in a more systematic fashion but others won’t. In short that’s rather uneven in fact, and I would find it interesting if there would be something more important."  **Psychologist 3:** "when patients arrive they complete an EPDS [Edinburgh Postpartum Depression Scale]"  **GP3:** "Despite being in private practice, I’m lucky enough to have a psychologist in the cabinet who has experience in perinatal care [...]. I have this support with the psychologist and in Valence we are lucky enough to have a service specialized in perinatal care in the CMP and a whole team that come see them at home [...] and an inpatient unit just for mums at the TEPPE [psychiatric hospital].”  **Midwife 1:** “The absence of child & adolescent psychiatrist or psychologist at the maternity. So that should be a little more generalized. To enroll a patient at the maternity when a psychiatric vulnerability happens without having solutions, I find that very frustrating.”  **Midwife 10:** "We are lucky to be very, very well equipped with an outpatient network including the LAPS. It's a mental health service that offers care during the entire peripartum, for both mothers and fathers. So screen during pregnancy, we refer them directly, so that already allows to anticipate postnatal depression well."  **Midwife 18:** "there is much positive in screening these peripartum depressions, referring them either to networks, to psychologists"  **Midwife 8:** “in term of difficulties, that’s true we don’t have C&A psychiatrists or psychiatrists at the clinic, so that’s complicated to take an advice.”  **Mother 1:** “Well I think I wouldn’t have told it! Maybe I would have told my mum at one point, and even I’m not sure. It was even easier to tell it to people from the outside”  **GP2:** “**I** think that very often the mums just don’t consult. [...] I rarely have mums coming to tell me, “I’m not well”. They don’t come on their own”.  **Mother 4:** “Childcare nurse of the PMI when she came, […] she told me, “by the way if at some point you see you’re not going well, you know we have a psychiatric nurse at the PMI who can come to see you”, and that remained in my mind. So when really I wasn’t well anymore, I told myself, “ok, I see the psychologist, my usual psychologist but that would be good to have someone specialized in postpartum”. […] So I called her and she came. […] And she told me about the mother baby unit.”  **Mother 1**: "I didn't necessarily see it coming. My delivery was great, well, a great discharge from the maternity. [...] I even was too dynamic. [...] we noticed it after 15 days. [...] And at that point, I started to get tired. My daughter had colic”  **Mother 4:** "In fact, the depression appeared when I returned to work. [...] I started, not to fall down, but to be less and less well. On the other hand, at that time I didn't know at all about postpartum depression, so I didn't understand what was going on, and I told myself, 'that's work'."  **Father 1:** “I often say, “I didn’t see it coming” and at the same time, when it happened, I immediately felt that there was something wrong. I didn’t keep it inside and actually I think that it helped. But I thought… Well, […] that’s just that maybe it wasn’t anchored in my software”  **Midwife 1:** "And then lastly there are patients who really have a problem but aren’t aware of it."  **Obstetrician 2:** “it has to be them who know, who recognize at which point that’s not normal anymore. Well, that they feel free to come to us saying, “now, I’m not well”.”  **Pediatrician 4**: “that’s rare that mums would like to say, “well, I’m unwell”.”  **Psychologist 4:** “one of these mums who had a depression that remained unnoticed because she was doing all that needed to be done for her baby but in the inside, it was a disaster” (p8)  **Midwife 5:** “because in private practice, we’re a little, well… [swallows] alone facing ourselves and the patient… and the couple. […] I find that we have many situations of depression”  **Psychologist 1:** “situations I receive during the postpartum are women […] who weren’t at all diagnosed or screened before […] and who will have the resource to look at their maternity leaflet where the name of a psychologist is written and who’ll arrive that way.”  **GP3:** “I was GP at the beginning, and mothers tell me… first, I ask them, if no one asks them the question, they won’t tell. And if you don't go looking for them, they won’t tell. […] I haven’t that problem or that reluctance to talk with parents.”  **Midwife 14**: “And that’s true that if we don’t ask the questions […], they will not necessarily tell us”  **Nurse 1** [female, 46 years old]: “That’s true a lot of mums tell us they don’t dare to speak up because they feel completely judged and that they’re ashamed”  **Mother 3:** “In fact, during that week**, a night when I could not fell asleep, I’m lying in bed with my phone and I type, “desperate mum, wishing to be dead”. And I come to “Maman Blues Association”. And I tell myself, “well, what’s this?” And I open the link and read and tell myself, “in fact, there are people to help me, I’ll hurry to contact them”, and I send a message, it was like 2 a.m., saying that I had twins, I’m going really bad, that I need help and I don’t know towards whom turning myself”.** […] **And there, they [Maman Blues] tell me, “listen, close by your town there is a CMP [medico-psychological center]”, and I don’t even know what it is, “and there is a parent-baby unit. You have to come see them**”.  **Father 1:** “I arrived through the forum of the association. […] The association that was via the website “1000 days”, Well you see I still have it, I receive my reminders from time to time. But “1000 days”, that’s how EM [president of Maman Blues] contacted me very rapidly.”  **Mother 3: "**The associations are still... I found that magical because it was the first help I could get my hands on (...) They gave me the contacts of all the others. “  **Obstetrician 3**: “sometimes they get the prompt thanks to the relatives. And there are fathers, or relatives, brothers and sisters, I don’t know, that will be very helpful […] the husband starts saying, “now, you’re not ok, you have to get help”, and the mums listen to their husband. Immediately, they feel that… They trust them and if their partner tells it, that means that they’re really unwell.”  **Midwife 21:** “so that fathers would be more aware, so that the relatives… That they wouldn’t be isolated with their diagnosis of peripartum depression”.  **GP2:** “because dads who bring the babies with a mum who is unwell, that’s dads who are going well. Dads on whom mums can rely.”  **Nurse 2 [female, 50 years old]:** “that’s the child’s grand-mother who contacted us directly to alert us on the mum’s condition. At this moment, the partner wasn’t present enough”  **Father 1:** “that’s my mother who advised me to install “1000 days”. […] she probably searched why I wasn’t going well.”  **GP 2:** “who bring the mum or the child and say their wife is unwell”  **Psychologist 6:** “there is all an awareness work to conduct […] that’s not mothers who consult on their own. It has to be someone pushing them to, either relatives or providers”  **Mother 2:** “I told it to my sister […] who is someone good at listening, and she told me, “Listen, I don’t know whether that’s normal or not, but anyway, you’re not going well, maybe you should consult a psychologist”. So I said: “well, I don’t know”. […] the energy that just coming to see a psychologist, to take my phone, to take an appointment, to express my milk represented… […] Pff! Coming tell my life… just that, that seemed insurmountable to me. But I thought about it all night and I told myself, “she’s right, I have to give a try and I can’t stay like that”. ”  **Woman SMI 5 [30 years old]:"a** chat to be connected with the psychiatrist and the maternity that follows you up. And possibly a forum for mums."  **Psychologist 1: "**There could be a space where women could ask questions [...] So maybe some kind of chat or a question forum..."  **Autistic woman 7 [33 years old]:** "that we could exchange on a dedicated forum. (...) I know there are a lot of people who like going on forums."  **Psychologist 6**: "I thought about chat too."  **Psychologist 2:** "And chats"  **Mother 1:** "why not little online chats, to have someone who can always answer to your question. (...) f I need help at some point: "I don't feel well, who can I contact?" Without being visual, not necessarily something on video, so that remains a bit anonymous. But someone who can answer you instantly."  **Midwife 17:** "they can benefit from discussing between women"  **Pediatrician 1:** "Well, I think that it is very, very important the testimony side, lived by the families: "I don't like my baby, he doesn't like me"."  **Midwife 12:** “**Maybe support groups. Because being able to discuss with other mums who’re living the same thing and have difficulties too, that avoids stigma. And to say, well, I’m not alone. […] I don’t have to be ashamed, I don’t have to feel guilt**”  **Mother SMI 5:** “Maybe to meet other parents. […] Why not a coffee meeting at the maternity ward, once monthly or something like that. During pregnancy, you have childbirth classes, but after childbirth you’re left on your own, so sometimes that’s not easy”.  **Psychologist 1:** "groups of mothers who had postpartum depression [...] I think they also need to know that they are not alone in this situation"  **Midwife 21:** "patients will perhaps more quickly... will sometimes more quickly get help on social networks [...] through #mypostpartum or videos like "matrescence" [...] Through experience... through "companionship" in quotes. [...] support groups facilitated by a provider"  **Midwife 26:** "hearing words, like that, from other mums or parents who have been there, or from providers who explain what that is, what the signs of depression are, what the warning signs are and what solutions are available."  **Pediatrician 2:** “I will speak as a man, but that’s anyway a seism to have children. […] But there is an experience that’s called a “group of fathers”. […] we had a feedback on these groups at the French Association for Ambulatory Pediatrics and that was absolutely fascinating.”  **Mother 4:** "Yes, and then more associations, or support groups or things like that. You see, I missed that at the mother-baby unit (...) We didn't have a support group with other mums."  **Mother 4:** “support groups for mums, that can be that too. Well, it makes the mum go out with her baby, which is very well and to discuss with mums who share the same experience. […] Also perineal reeducation, that’s very good because it makes the mums go out, and to me it was doing me good. By the way, it started going worse when I had no longer outside appointments. […] Because at the beginning you’re so on the baby that you forget to do things, and you don’t feel like doing things, but at the same time you have to force yourself to do some things only for yourself."  **Mother 4:** "a kind of manual saying “there are several possible care options”. (...) that there are many ways of dealing with the problem, not only medical ones, but also support groups for mothers. First of all, that forces the mother to go out with her baby, which is very good, and then to discuss with other mothers who are going through the same thing."  **Mother 3:** "Then in a mobile application, maybe a possibility to organize support groups, because frankly, that’s very rich and nourishing and I find that it allows to... on the one hand to remove taboos if you feel confident in the group, and also to concretize the discussion."  **C&A psychiatrist 1:** "peer-support groups." |
| **Facilitating access to SPMHC for fathers**  Patient level:  Access through the mother, the baby or via health providers  Resources for fathers | **Psychologist 4:** “it’s harder to catch the fathers”  **C&A psychiatrist 2**: “mothers prescribe fathers […] that’s the mother who realize the father isn’t very well or not as usual and sends him to care.”  **Psychologist 1:** “what they ask is to be supported in their parenting through the baby more than to be mentally supported by a therapist.”  **Pediatrician 3:** “there are more and more fathers who are in the race, they’re much more present than before. Sometimes there is a father who isn’t well and what is complicated that’s when the father is anxious. […] We go into things that are difficult to contain. Or anyway, I have no ease for that.”  **Father 1**: “I immediately told my partner. […] And we relatively rapidly reacted. My partner told herself that we could ask the midwife who cared for us before childbirth and she oriented me to…”  **Psychologist 8:** “regarding fathers, even if things have much evolved, there is still a long way to go. **We recently had a father who was hospitalized at full time. That was actually a first. […] her partner died one month after childbirth. […] there was all an orientation worked with a maternal center that was ready to receive that father and his baby. The answer of the Department on the funding was, “that father has just to return to work. He has better confide his baby, to place him and to return to work. That’s not his place to be with his baby.” […] That was really surprising.** […] Finally they were hospitalized in our service, and he actually told me to which point, well, first, for a man to arrive in a service where almost everyone was a woman, not simple! And he told about the shame he was feeling as a man to be in that process to get hospitalized, to seek help, and yet he told, “I have to admit I really need that help”.  **Father 1:** "**something that could work well, well that worked for me, [...] that’s indeed testimonies. Or people who could be listening [...] but anyway testimonies, maybe before, to realize that that does exist, that there are some people experiencing it, that we won’t all experience it in the same way, uh... I think that's never hidden, that fathers can be affected, but that would be nice if they were more represented. [...] That’s very often associated with the mum, and that would be nice hear some testimonies from fathers too. So testimonies for sure."**  **Pediatrician 2:** “I will speak as a man, but that’s anyway a seism to have children. […] But there is an experience that’s called a “group of fathers”. […] we had a feedback on these groups at the French Association for Ambulatory Pediatrics and that was absolutely fascinating.”  **Mother 4:** “I know that now mother-baby units receive parents too, fathers too, and that’s very good because my partner, was not affected, well he wasn’t affected, he did not have a depression even if he had a little backlash when I came back from the mother baby unit, he was quite exhausted. But he staid the course, let’s say. But I think that in some situations, that’s the whole family that should be taken care of. Sometimes when that’s a first child, the couple is a little shaken, the father can be completely off-balance too”. |
| **Women with SMI and autistic women specific needs and expectations**  Attending to a dual set of needs (oneself and child)  Additional personalized support  Communication between providers and a referent provider /sharing information to avoid repetitions  Psychoeducation  Joint crisis plans*  Peer support / support groups  Additional training for health providers (knowledge) | **Woman SMI 9 [31 years old]: "I mean, how can I balance my life? For example, take a typical day, how can I take care of myself while taking care of my child?** And to take time especially for myself, that’s important. Especially for myself, I think, to prevent some depressions or things like that, not to feel guilty if I don't manage to do certain things. Well, it's general, I can't be more specific, not feeling guilty if, for example, I don't manage to put the laundry away and that's lying around, will I... is that necessarily a negative thing? Well, handling daily life a little bit"  **Woman SMI 1 [30 years old]:** “Well, then, that’s coping as a mum. That’s also stressful. Not having only to think about yourself, but also to have the responsibility of another small human being. That would be also some things to learn.”  **Woman SMI 2 [22 years old]:** “That’s more impatience, more to not managing to find some time for myself. I personally need much to be alone, and when I can’t, that’s complicated. And I know that with a child that’s complicated to… to not manage to handle my need for being alone because I’m in a low mood period and that… yes, a child takes so much time and energy…”  **Woman SMI 9: "**Daily life activities: housework, meals, food, care, everything that is... related to care... medication. How could someone help me to take my medication properly? Taking medication is important, all that. So how do you deal with mood swings, times when you’re not going so well, how do you find moments when you could take the pressure off and get back on track? Food, daily life, medication, uh... and knowledge about the baby, the infant a little. And relaxation too, wellness. Everything that is yoga. That could be it too, wellness. Yes, that's it. And specialized tools, documentation, to get information. Finding documentation."  **Autistic mother 10 [37 years old]: “**To have less… all that is mental load, organization, all that, that’s very hard. While working. Well, everything is doable but doing everything at the same time, that’s very hard for me. I think for everyone, but that’s true that given my condition, that’s complicated”.  **Woman SMI 9**: “there is always that fear of being a bad mother, being an abusive mother too… There is that fear of being an abusive mother when the child arrives. The fear of not being up to, the fear of… There is the fear that if I’m too tired, if I don’t get support and if I’m a bit lost, during crisis periods could I become abusive? I know I won’t always be perfect, but could I become an abusive mother? […] How to manage to get some support and to get moments for me too? Moments during which I could take care of myself to take better care of the child?”  **Woman SMI 2:** "**And maybe to have a follow-up also maybe more important than a person who doesn't have bipolar disorder.** So to see more people. To be more secure. Because I also have a big fear of decompensating, I tell myself, “that’s possible that it could get worse, that I could decompensate.” Seeing more providers and seeing them more often, that's... **that's reassuring."**  **Woman SMI 9: "**to understand the first months of an infant. Meaning... uh... maybe know all the little tools to get organized when you have attention problems, when you tend to be a little more disorganized than organized. Having techniques. Getting organized in your daily life. Where to find... where to go? Do I need to go to a psychomotor therapist if the child has this, or do I need to go**."**  **Woman SMI 4 [37 years old]**: **"**I would maybe need to have different types of providers in front of me. (...) Because depending on the questions you have, you won't necessarily have the same questions depending on which doctor you see. I had questions for my psychiatrist about my treatment. And I'll have questions about pregnancy, so rather with [a specialized midwife in perinatal psychiatry]. Because maybe my psychiatrist won’t know how to answer to all questions related to pregnancy**.”**  **Mother SMI 7 [31 years old]**: “Well, I know myself pretty well, but maybe when you’ve got a specific psychiatric disorder, to be told again about the indicators, to be told, “well, red flag. Maybe that’s not ok. You should call the psychiatrist right now”. Well I know myself so I know when I’m not ok, but, well, making a review with a psychiatrist specialized in pregnancy.”  **Mother SMI 8:** "I was followed by a psychiatrist. And that's good to have a good follow-up and to ask for help. We had a TISF a little later, as soon as we could take the necessary steps, as quickly as possible. What is good is to organize beforehand (...) And the TISF was really a great help. She could come during the day, 3 hours, 2 hours to take care of the baby, do the housework. And there are also (...) household helpers. (...) they're really nice people, and I know that when you're psychologically vulnerable that's hard to ask for help, but that's important to ask for help."  **Woman SMI 4:** "a follow-up of the interrogations Because you indeed have questions before. And surely we have others that come up during the pregnancy, and others after. And to me, having three different time points allows a real follow-up of the evolution of the patient’s interrogations”.  **Mother SMI 7:** "I changed psychiatrists during my pregnancy, so it was a bit complicated. I had trouble with the medication aspect, but I had a reference psychiatrist who dosed me well with lamotrigine so that I didn't have too much in my blood either, because I almost had an overdose with the other psychiatrist. So he followed me every month, even by phone when I couldn't come. (...) when I wasn’t going well, I called, I could make an appointment. So that's very, very important."  **Woman SMI 1:** “I know I have doctors following me up and knowing my story. So that’s a real support for me, because I know that if one day, there are trigger signs, I’ll know to who orient myself and for me that’s important too that there would be persons, in fact providers who could help me and that’s even better if they know me and my story.”  **Women SMI 6 [36 years old]:** “yet, I have resources from the medical staff, in a broad sense, that allow me to quickly ring the alarm bell if there is a problem. So the fact to have a nurse at the CMP, a psychologist at the CMP, a psychiatrist […] the possibility to alert them, meaning phoning, leaving a message or having the person directly, if something is going wrong, that’s a huge resource. […] Because actually, you know that if there would be a problem, you’ll be able to call and be received”.  **Mother SMI 5:** “my psychiatrist monitored me closely, I saw him every week. I was also supported by the SAMSAH [medico-social service for adults with disabilities]. And a few months after childbirth, I asked for a follow-up by a TISF. Well, I already knew the SAMSAH. Obviously I knew the psychiatrist. That was the PMI who proposed the TISF, because they monitored my daughter medically, so I asked to the childcare assistant for help at home so that I don’t stay alone handling the house, the animals, and the kid.”  **Mother SMI 8 [40 years old]**: “the lack of care. I found it hard to be abandoned […] after childbirth, that I didn’t have a doctor anymore and all that. I find the follow-up important, and I believe I wouldn’t like do it again with such a precarious situation […]. I would not like to be abandoned. Actually, the part on maternal healthcare is important. […] I prefer to be cared for. […] Obviously, I would not like my child to be put in children care home after childbirth. I have the impression that that’s the consequences of a lack of care before. […] that’s true you can become pregnant without having planned it […]. But rapidly, you can do something before it becomes too catastrophic and there is a need to place the child in foster care because the mother is unwell. And I have the impression that as soon as you become pregnant, you have to organize yourself so that could go well”  **Woman SMI 9:** "For me that would be more something that could balance my life. That would more be a tool that could balance my life, whether it's a balance between my health and daily life (...) maybe some tips (...) what can I do when the child cries too much? What can I do if I haven't slept for a few days because that has an impact on my health? Things like that."  **Woman SMI 9:** "I tell myself... I don't want to be too stressed or anxious and that it could have a negative impact on the baby. I don't want to be too anxious and start to enter a parallel life, like... actually, I'm afraid that this pregnancy will bring me so many emotions, that I could be afraid to drop out a little bit. It's something I'm waiting for and I'll be afraid that it would bring me too many emotions that could in fact promote a crisis."  **Woman SMI 9:** “not reading, but a small form for persons who have this health issue. And that would at the same time ask you little questions on your daily life and how you could do better. Well, a small roadside companion in these difficult times. A little guide like that."  **Autistic woman 5:** "I hope that for people like me in particular, that there would be a reinforced psychological follow-up after pregnancy."  **Woman SMI 6**: "Home visits (...) Well, home visit that’s if that's really complicated and that, well, that’s to avoid the hospital. I would say that you have to avoid the hospital at all costs. Because being in the hospital when pregnant, I think that's really horrible, right? That’s already horrible when you're not pregnant, but now, pregnant, it's unmanageable.”  **Woman SMI 4: "**That means a time with a health provider, uh... a time to talk about that, a time dedicated to that, during which in any case, I would have the opportunity to ask questions to the person supporting me, to the health provider. This means that I am not left alone with my questions without having the possibility of discussing them with a doctor or a health provider. So, well, already that means that these questions are taken into account and that this space exists."  **Mother SMI 7:** "that’s a consensus between a psychiatrist, a midwife and then potentially a psychologist because with medication changes, some support is needed, well that can quickly turn bad. That can quickly become quite serious (...). Yes, I would put a trio of psychiatrist, midwife and psychologist so that throughout the pregnancy [...] they would be aware of the progress and the problems you can have. That each one could contact the other and then that tripartite decisions could be taken."  **Woman SMI 3 [28 years old]:** "Because each time you have to re-explain and that's very heavy to re-explain each time your problems to a different person. So put as few interlocutors as possible and make syntheses."  **Woman SMI 6:** "I would even say that having even one phone call a week, to say if you’re going well or not. One phone call per week could very good during pregnancy (...) I see it as a space for talking and as a space for containing, well, for containing what could be a little overflowing in the illness. So at the same time, maybe we should have a crisis plan, meaning, anticipating a protocol that would tell, “well, we should call that person and not too much that other person. (...) that hospital and not that one, that clinic and not that one.”  **Mother SMI 7: “**a referent who can refer us to a midwife who knows about psychiatric illnesses, who can come with us at the maternity to see a referent. I had to grab information where I could, that was complicated. Maybe something like a contact that could help you set up the network that will support you during pregnancy. At the limit, someone who does every month a little psychological review, like the midwife following you up. Because you’re in that situation [having a mental illness], that we would have this support, even by phone or whatever, really that everything would be organized.”  **Mother SMI 7**: "if the midwife sees that the mum is a bit fragile, that she could refer her to a psychiatrist or a psychologist. [...] I was able to see when I was unwell. But someone who doesn't know herself well, that the midwife would say, “oh, there's a problem", that the psychiatrist and the psychologist would also be aware of it, and maybe would think about another medication, (...) yes, that there would always be communication between the three of them so that, when the young mum has an appointment, well, that there would be someone who could alert if she finds her unwell. Because midwives are as capable as psychiatrists of alerting when a mum is unwell.”  **Mother SMI 7:** "Well, actually, more than a tool, that would allow to put all the doctors who are involved in the follow-up around a table and that there would be a sort of… let’s say, only one interlocutor, that the mum would not have anymore to… I would say to worry too much, because that’s true, to worry too much to look for the right person to contact… Well, that there would be her small network that would automatically build itself because that’s super heavy"  **Woman SMI 6:** "It should be seen how we work in relation to confidentiality, I would say by making the link between (...) the parties monitoring the pregnancy. [...] There could be a phone call made to [a specialist midwife in perinatal psychiatry] by the hospital midwife well, some sort of link between the institutions."  **Mother SMI 5:** "To be able to communicate with the medical team that will follow you in the maternity ward. A tool like that could be good tool, if it also involves the usual psychiatrist. That would allow you to have the psychiatrist, the obstetrical team and the person concerned in fact. I think that could be quite good. (...) Well, there is no link between psychiatrists and maternities, so it that’s very delicate in fact. So I think that could be... just creating a link and that communication would be easier."  **Mother SMI 7:** "A platform where doctors, well, health providers, have access to a file recording all the appointments and that everyone could access to it. And maybe put some kind of alerts on specific appointments. (...) that everyone would be aware, that everyone would see the decisions. So that the mother wouldn’t have to repeat to her psychiatrist if that's not going well. Maybe there could be a connection with the midwife. Because always repeating when that's not going well, it's... sometimes it's a little hard."  **Autistic woman 5:** "Well, I know that's possible to have some kind of platforms, well, after there are all the security and data protection issues that I don't master, but if we could have shared platforms. (...) That each provider would put notes for the others, and that it would make a basket that everyone could consult."  **Woman SMI 2:** "that all the doctors caring for me could have the same level of information. So that they could know how to react, the impact that could have. (...) In fact, the fact that the doctors would all be informed at the same level and could know a bit how you’re doing, how things are going, that’s reassuring. And then I think if my doctor could be in the loop, that would be a good thing, because he told me that regarding bipolar disorder he wasn’t… well, he doesn't really know. But, well, what I need above all, that’s to have, to know that when I come to see the providers that will care for me, that it could… that they could really be informed about that illness. And that I could feel secure because I know that they know what that involves, what impacts that will have."  **Autistic woman 5:** “Something that could be good would be to be able to contact providers by email for example. That doesn't mean an instantaneous answer, but would allow me to pose the question I’m asking myself right away and then the answer comes at the appropriate time, but at least it relieves me of that burden.  **Woman SMI 6:** **"**And I really need these places of listening where we can talk about the treatment during the pregnancy, about what it means to be a parent, what values we have when we are a parent, will the child be affected by the disorder, as it is hereditary... are we taking a big risk?"  **Woman SMI 4:** “To me that allowed getting out beliefs. Meaning that we have a very, very big imagination. So when you don’t know, you invent answers. So, the fact of being able to talk with a doctor, that allows reorienting and making things objective. That’s how I think about science. Meaning you have a question. You could have an objective answer with probabilities. And once you have the probabilities, the decision belongs to you. You take the risk or not, but anyway you take the risk knowing the probabilities.”  **Mother SMI 5:** "Well, for example, the CRAT website [center of reference for teratogenic agents]. To know more easily the compatibility of medications with pregnancy and breastfeeding. To have small information sheets on withdrawal syndrome, on general pathologies of pregnancy, thus on gestational diabetes, etc. To have information on the progress of a delivery. To know a little bit... likewise, the compatibility of the epidural with the medication. That's it, things like that.  **Woman SMI 9:** "I heard that neuroleptics induce tremors... a withdrawal effect, or a... and that worries me. (...) There may be a withdrawal effect due to medication I’m taking during pregnancy. Will be any impact for the child at birth?"  **Woman SMI 4:** "Data for example also on the impact on relatives. Meaning if I ever have a major relapse at childbirth, when the baby arrives, what impact will that have on my partner? So well it, how things could be experienced by the partner too."  **Woman SMI 2:** "What missed me was to someone specialized in mood disorders helping me to understand everything that happens in bipolar disorder. (...) What also miss me is that… thanks to my friends, I know (...) the problems that pregnancy can have (...). But when it comes to bipolar disorder well, there's no one, just like in marital life, who tells you what it could induce. And I need to understand, I need to... that reassures me because when it happens, well, you don't feel guilty either, you tell yourself that it's just the way it is, that it's the illness."  **Woman SMI 1: “a document that could allow thinking and analyzing my worries and my fears and on which I could then write what I would like to put in place. […] if we find ourselves in that situation, what would we do, which actions would we put in place.”**  **Woman SMI 1:** "Well, that’s a document so for the couple, and for me that’s also to be shared with the doctor, or the person who could follow me, so that there's a good understanding of the person, her history, her worries. Well, I think that it will also be up to the doctor [...] to fill in a part of the information, perhaps. Saying... The doctor could say: "Well, I know X, she has had this or that situation, today she is in such and such condition". And the day when, for example, there would be something that happens, for examples symptoms that reappear, if [...] there would be a new doctor following me up, that he would have all the elements inside that document.”  **Woman SMI 1:** “describe the fears too… […] that the person could describe her fears. And to envisage things. To tell herself that in this or that situation, what should absolutely be done or what should absolutely not be done. That could be important we could mention it.”  **Woman SMI 6:** "a space for containing, well, for containing what could be a little overflowing in the illness. So at the same time, maybe we should have a crisis plan, meaning, anticipating a protocol that would tell, “well, we should call that person and not too much that other person. (...) that hospital and not that one, that clinic and not that one.”  **Mother SMI 7:** "Well I would have liked to have some sort of support group... well on bipolar disorder or on other disorders, because that looks like anyway. But really a group of future mums, or to include mums who have already had a baby, to really discuss on how it went, how they handled, because one feels super alone already when one has a psychiatric disorder, but there, I... I feel super, super alone. And to discuss with other people sharing the same problems, or who went through this stage and are doing well, because you always have the negative examples when you need the positive ones. There are some! Well, I missed that a lot."  **Autistic woman 6 [36 years old]:** “Maybe possibilities to meet others, or at least support groups or the support of persons who already went through this, even if that’s delicate because no one is exactly the same when this happens. But I find that could be very helpful and reassuring…”  **Mother SMI 5:** "for the postpartum, why not being able to get in touch with mums who could also have a disorder and who just had a child. It doesn't have to be by name, but there could be a forum for young mums, for example, to discuss life with a baby when you have a disorder. Or that there could be the possibility of having links, or I don't know. Something like that."  **Mother SMI 7**: "I needed some people who experienced the same thing, and that's super complicated, because I was going to Argos 2001, that’s an association where bipolar people and caregivers meet (...) I don't like much to go on Facebook, but I found a group when I asked my question, and that's true that I had quite a few mums who answered. So I saw that there were mums with bipolar disorders, who even had several children, and in relation to sleep, because that was my biggest fear, who told me "be careful, you really need to rest. You really have to rest when the baby is sleeping, that's what we tell all the mums, but you really have to do it." That's it, so I had some advice like that, and that was good for me to tell myself "I'm not alone. There are other people". (...) So, well, that reassured me that Facebook aspect, except that I'm not a fan of going on these social networks, but it was the only place where I found a diversity of people with bipolar disorder who are parents."  **Mother SMI 5:** “Well, at that moment**, my fear was mainly about the medication. But I was lucky enough to have a very good psychiatrist who asked […] a psychiatrist trained in peripartum to have a treatment compatible with pregnancy and to have the less withdrawal syndrome as possible after childbirth**.”  **Mother SMI 5: “**I told several providers about the withdrawal syndrome, they all looked at me with bulging eyes. […] They never heard the word “withdrawal syndrome” during their studies, so that’s very French”  **Psychologist 3:** “I don’t know if you can say postpartum depression in a psychotic patient who has a baby.” |

*Theme identified only by non-parents
