# Supplementary material for: Toward recovery-oriented perinatal healthcare: A participatory qualitative exploration of persons with lived experience and health providers’ views and experiences
Source: Eur Psychiatry. 2023 Oct 20;66(1):e86. doi: 10.1192/j.eurpsy.2023.2464 (PMC10964275; doi:10.1192/j.eurpsy.2023.2464)
Supplement: Dubreucq et al. supplementary material 7 — Dubreucq et al. supplementary material [file S0924933823024641sup007.docx]

**Supplementary table 7. List of abbreviations**

C&A psychiatrists: child and adolescents psychiatrists

CHPs: childcare health providers

HPs: health providers

MHPs: mental health providers

OPs: obstetric providers

PLEs: persons with lived experience

PMH: peripartum mental health

PMHC: peripartum mental health care

PMHD: Peripartum mental health disorders

PPD: peripartum depression

SDM: shared decision-making

SMI: serious mental illness

SPMHC: specialist peripartum mental health care

SPMHS: specialist peripartum mental health services

UK: United Kingdom
